# The N-terminal Helical Region of the Hepatitis C Virus p7 Ion Channel Protein Is Critical for Infectious Virus Production

**DOI:** 10.1371/journal.ppat.1005297

**Published:** 2015-11-20

**Authors:** Margaret A. Scull, William M. Schneider, Brenna R. Flatley, Robert Hayden, Canny Fung, Christopher T. Jones, Marieke van de Belt, François Penin, Charles M. Rice

**Affiliations:** 1 Center for the Study of Hepatitis C, The Rockefeller University, New York, New York, United States of America; 2 Bases Moléculaires et Structurales des Systèmes Infectieux, IBCP, Labex Ecofect, Université Lyon 1, France; CNRS, UMR 5086, Lyon, France; The University of Chicago, UNITED STATES

## Abstract

The hepatitis C virus (HCV) p7 protein is required for infectious virus production via its role in assembly and ion channel activity. Although NMR structures of p7 have been reported, the location of secondary structural elements and orientation of the p7 transmembrane domains differ among models. Furthermore, the p7 structure-function relationship remains unclear. Here, extensive mutagenesis, coupled with infectious virus production phenotyping and molecular modeling, demonstrates that the N-terminal helical region plays a previously underappreciated yet critical functional role, especially with respect to E2/p7 cleavage efficiency. Interrogation of specific N-terminal helix residues identified as having p7-specific defects and predicted to point toward the channel pore, in a context of independent E2/p7 cleavage, further supports p7 as a structurally plastic, minimalist ion channel. Together, our findings indicate that the p7 N-terminal helical region is critical for E2/p7 processing, protein-protein interactions, ion channel activity, and infectious HCV production.

## Author Summary

Hepatitis C virus (HCV) infection can lead to significant liver disease and, without a vaccine, continues to pose a significant public health threat. The viral p7 protein is a multifunctional protein that is required for infectious virus production via its role in orchestrating virion assembly and its activity as an ion channel. However, while there is accumulating structural information on p7, there is no consensus on which conformation(s) exist during a natural infection or how structural elements relate to p7 functions. By comparing two prominent, yet highly divergent models of p7, we identified one region of structural similarity–the N-terminal helical region. While mutagenesis screening of other regions of the protein are in keeping with p7 conformational flexibility, mutations within the N-terminal helical region had a significant impact on infectious virus production, due in part to a loss of efficient E2/p7 cleavage. We further postulated the precise functional impact of mutations throughout p7 by homology modeling and demonstrated tolerance for diverse amino acid substitutions for specific N-terminal helix residues with putative ion channel defects. Together, these data not only support p7 as a structurally plastic, minimalistic ion channel, but also provide extensive insight into the p7 structure-function relationship and highlight the importance of the N-terminal helical region in E2/p7 processing, protein-protein interactions, ion channel activity, and infectious HCV production.

## Introduction

Over 130 million people worldwide are at risk for liver fibrosis, cirrhosis, hepatocellular carcinoma, and end stage liver disease as a result of hepatitis C virus (HCV) infection [1]. These complications of infection have made hepatitis C the most common indication for liver transplantation [2]. Further, while novel direct-acting antivirals targeting HCV have dramatically improved clinical outcomes, no vaccine exists to date, and the disease burden is expected to increase over the next decade [3].

HCV is a hepatotropic, plus-strand RNA virus of the *Hepacivirus* genus and *Flaviviridae* family [4,5]. IRES-mediated translation of the 9.6 kb HCV genome yields a single polyprotein that is proteolytically cleaved to produce 10 mature viral proteins that participate in viral replication and assembly of nascent virions [6]. The p7 protein, located at the junction between the structural and non-structural proteins [7], is a small, 63 amino acid integral membrane protein [8], predominantly localized to the endoplasmic reticulum (ER) [9].

In the context of the HCV life cycle, p7 is dispensable for viral RNA replication [10] but required for infectious virus production [11,12], although it does not appear to be a structural component of the virion nor is it required for HCV glycoprotein-mediated entry [9,13,14]. Accumulating evidence suggests that p7 orchestrates intracellular viral protein distribution [15–17], at least in part, via an (direct or indirect) interaction with the viral NS2 protein [16,18–22]. Additional interactions have been suggested with core at the genetic level [23] and with E2 by immunofluorescence-based colocalization and FACS-FRET methods, although coimmunoprecipitation of p7 with HCV glycoproteins in HCV-replicating cells has yielded disparate results [9,24]. Further, yeast two-hybrid and bioinformatically-predicted cellular binding partners have not been further validated [25–28].

Based on the ability of p7 to alter membrane permeability, it has been classified as a viroporin along with HIV-1 vpu and influenza virus M2, among others (reviewed in [29]). p7 ion channels are sensitive to hexamethylene amiloride [30], long-alkyl-chain iminosugar derivatives [31], and–depending on genotype [32,33]–amantidine [34], all of which inhibit cation channel activity in artificial membranes [34,35]. The importance of p7 ion channel function for HCV has been demonstrated by correlation of intravesicular pH modulation and infectious virus production in cell culture [36]. This activity has been hypothesized to enable proper glycoprotein folding, protect against premature degradation [37], or guard against acid-induced conformational changes [14,36,38,39].

Structurally, initial computational modeling predictions [18,40], refined by NMR experiments [22,41,42], indicate that p7 monomers adopt a “hairpin-like” topology consisting of an N-terminal helix and “turn” sequence upstream of two transmembrane segments that are connected by a hydrophilic, positively-charged cytosolic loop containing two highly conserved basic resides. The N- and C-termini are oriented towards the ER lumen and may provide a platform for interactions with viral or host proteins [18,43].

The intricacy of p7 structure is further complicated by p7 homo-oligomerization. Based on the typical oligomeric structures of viroporins, p7 subunits reside side-by-side in classical hexameric and heptameric models [40,42,44,45]. Molecular dynamic simulation of p7 oligomers, based on the monomeric model put forth by Montserret et al. [41], suggest that multiple oligomeric states are feasible and that p7 is structurally plastic and may adopt multiple conformations during oligomerization and/or as a function of its lipid environment [44,46]. In contrast, the recent NMR structure of hexameric p7 [47] exhibits an unusual architecture where part of each p7 subunit crosses over to interact with all the five other p7 subunits. The resulting rigid structure is reminiscent–albeit comparatively inverted–of single-particle electron micrographs of p7 that depicted a “flower-shaped” architecture [43].

Despite the increasing amount of structural data on p7, there is no consensus on which conformation(s) exist during a natural infection or how structural elements relate to p7 protein-protein interactions, cation selectivity, and ion channel gating. Influenza virus M2 and HCV p7 can partially functionally complement each other [36,48], yet analogy to HIV-1 vpu or influenza virus M2 provides limited mechanistic insight given the divergent structural features and diverse functions described [29]. Modeling of homo-oligomeric assembly [35,40,44] and electrophysiology experiments [49] indicate that the first transmembrane helix of p7 lines the pore, and the C-terminus (including TMD2 and unstructured termini) has been proposed to interact with other proteins [41]. Further, residues potentially involved in cation selectively and gating or intra/intermolecular stability have been postulated [12,41,47]. However, while mutation of two basic residues, K33 and R35, within the cytosolic loop, supported their role in ion channel function [36], none of the putative pore-lining residues studied to date by mutagenesis are essential for p7 ion channeling *in vitro* [12,41,49–51].

A comprehensive analysis of residues in key structural regions has not been performed. While the amino acid sequence of p7 is not highly conserved, extensive physico-chemical conservation [41] suggests that the overall p7 structure is similar across genotypes despite variability among individual amino acids. Here, we aimed to probe p7 plasticity and functionality using a combination of mutagenesis and molecular modeling approaches. Our data indicate a critical role for the N-terminal helix region of p7 in modulating E2/p7 cleavage and further support p7 as a structurally plastic, minimalist ion channel through interrogation of specific N-terminal helix residues predicted to point toward the channel pore.

## Results

### Mutagenesis of p7 supports a model of structural plasticity

**Fig 1:**
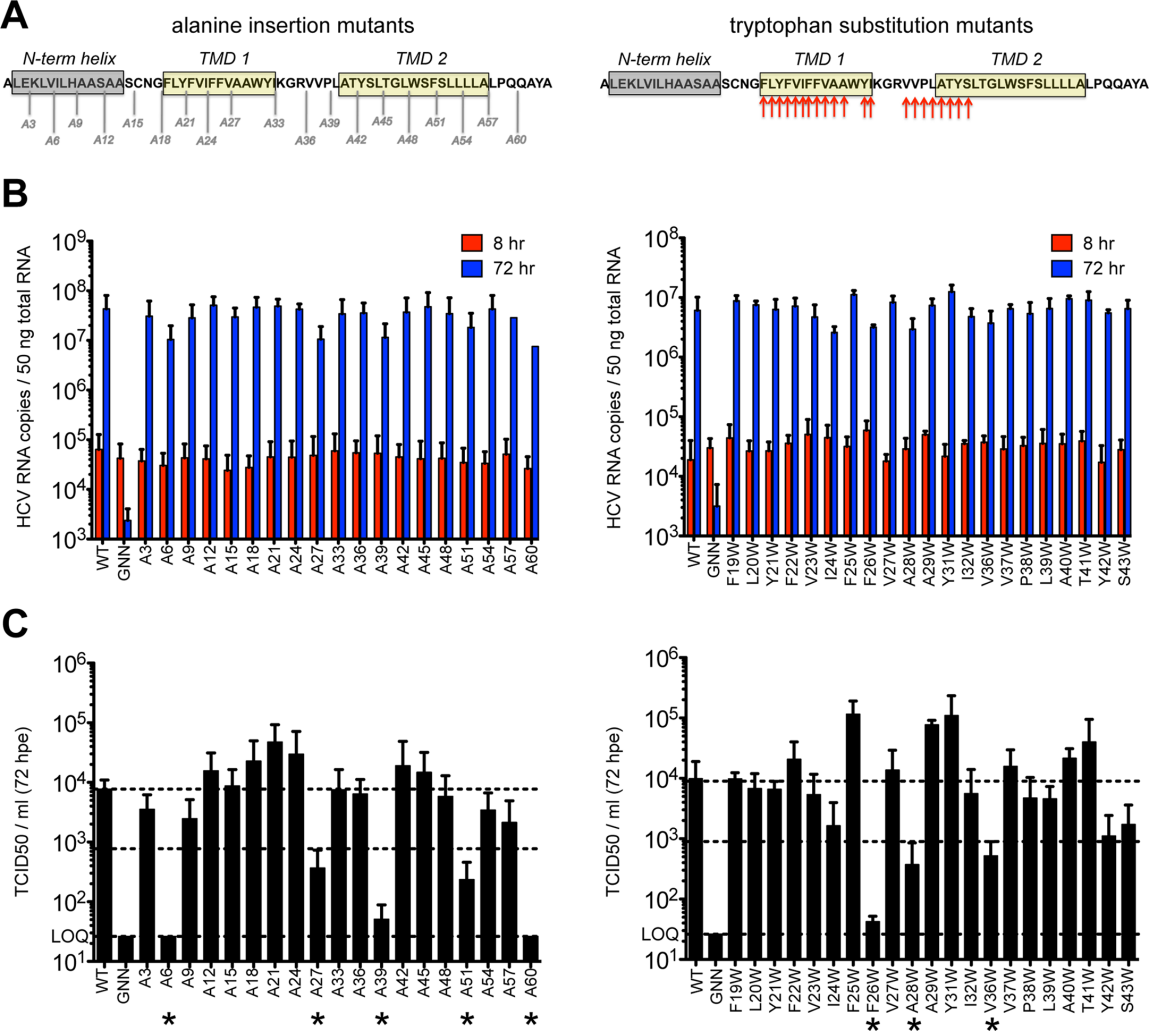
Large-scale mutagenesis of p7 has little impact on infectious virus production. **A)** Cartoon of the alanine insertions and tryptophan substitutions generated in J6/JFH p7. The secondary structure boundaries shown were previously deduced from p7 NMR data using HCV-J (genotype 1b) in 50% TFE [41]. **B)** HCV RNA levels in Huh-7.5 cells determined 8 and 72 hours post-electroporation (hpe). GNN is a replication-defective genome containing a double mutation within the RdRp motif of NS5B (GDD to GNN). **C)** Infectious virus in the supernatants of electroporated Huh-7.5 cells quantified by limiting dilution assay. Asterisk (*) indicates p7 mutant genomes that yielded >1 log decrease in TCID50 / ml compared to WT (compare middle and upper dashed lines). LOQ: lower limit of the limiting dilution assay. Bar graphs in all figures depict mean, +/- standard deviation across a minimum of 3 independent electroporations. Results were confirmed using RNA generated from independent transcription reactions and replicate experiments.

Previous reports indicate that p7 is not required for viral RNA replication but is required for infectious virus production. Modeling data indicate that in addition to the hexameric and heptameric forms of p7 demonstrated experimentally, tetrameric and pentameric oligomers may also exist, at least transiently [44]. To provide biological evidence of p7 structural features and define regions important for functionality, we generated two p7 mutant panels in the context of the J6/JFH infectious clone–one in which an alanine was inserted after every third amino acid throughout the entire length of the protein to perturb p7 structure and a second in which tryptophan substitutions were made throughout the transmembrane domain regions at residues 19–29, 31–32, and 36–43 to probe intra- and intermolecular interactions as well as amino acid hydrophilic pore- vs. hydrophobic bilayer-facing orientation (**Fig 1A**). Mutation of conserved basic residues K33 and R35 in the cytosolic loop was previously shown to impede ion channel activity and block infectious virus production both *in vitro* and *in vivo* [11,12,17,36,48,52]; thus, we excluded these from our analysis. Quantification of cell-associated HCV RNA at 8 and 72 hours post-electroporation indicated that over this time frame all mutants replicated with wild-type (WT) efficiency (**Fig 1B**), exhibiting a mean 495-fold increase in RNA copies per 50 ng of total RNA. Several mutants exhibited marked reductions (>1 log decrease vs. WT) in extracellular infectious titers (A6, A27, A39, A51, A60 and F26W, A28W, V36W) (**Fig 1C**), and these data correlated with slightly lower levels of HCV RNA at 72 hours post-electroporation (**Fig 1B**), likely due to reduced virus spread within the culture. Of these, A6 and A60, located near the N- and C-termini, failed to produce any infectious virus. Surprisingly, the majority of p7 mutants were competent for infectious virus production, including mutants with alanine insertions within the first transmembrane domain, a region considered important for ion channeling. Further, several mutations (e.g. A21, F25W and Y31W) even yielded titers above those obtained for WT virus indicating that p7 can accommodate these genetic changes (**S1 Fig**). These results support a model of p7 structural plasticity in human hepatoma cells replicating full-length HCV genomes.

### p7 hexamer models predict that the N-terminus modulates p7 function

**Fig 2:**
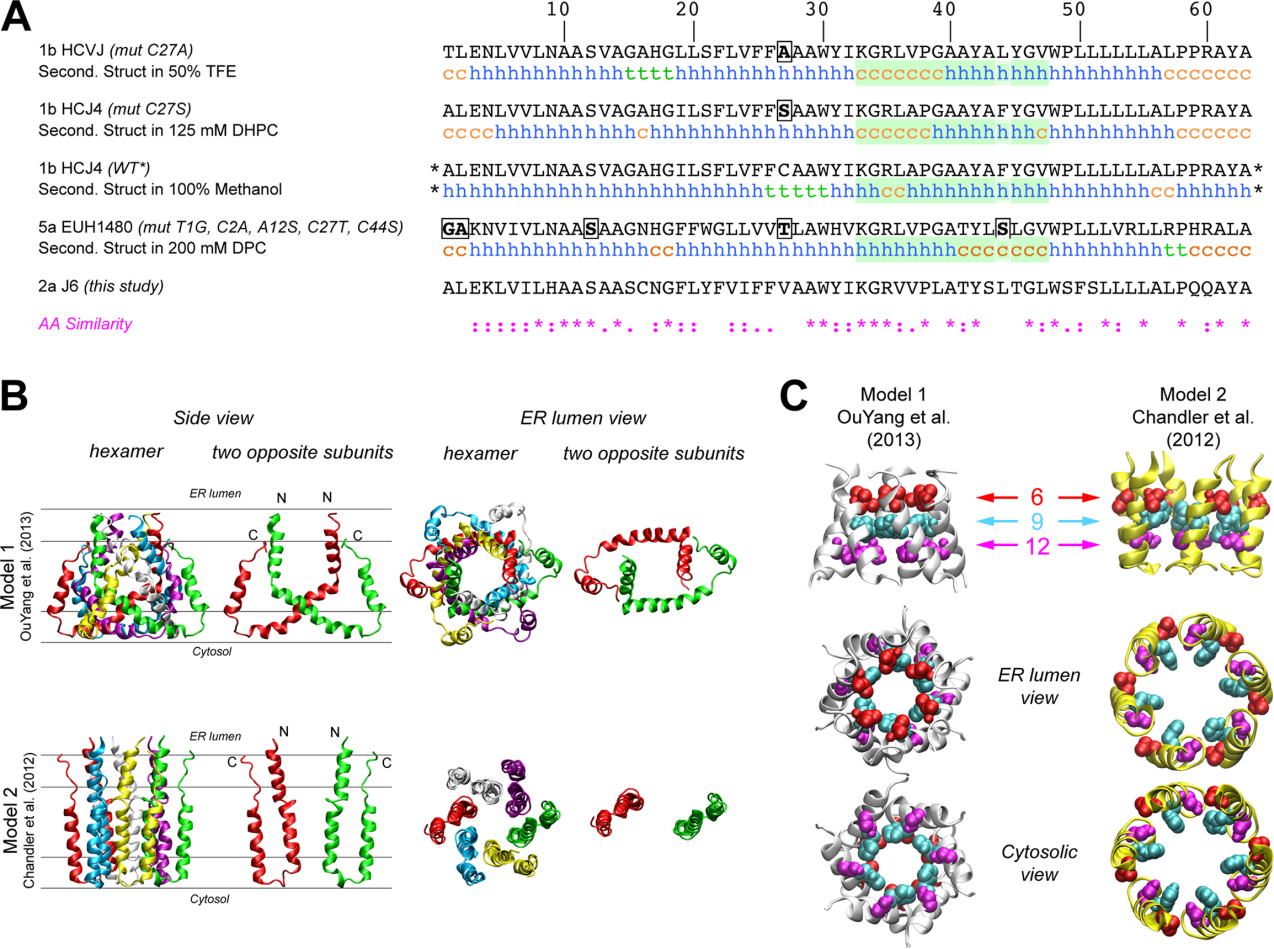
Comparison of p7 structure models identifies the N-terminal helical region as a potential key modulator of p7 function. **A)** Comparison of amino acid sequences and NMR secondary structural elements of p7 as determined by NMR in 50% TFE [41] (PDB entry, 2K8J), 125 mM DHPC [22] (PDB entry, 2MTS), 100% MeOH [42] (PDB entry, 3ZD0) and 200 mM DPC [47] (PDB entry, 2M6X). The stars (*) indicate that additional amino acids (N-terminal FLAG tag and C-terminal polylinker) were fused to this construct. The green box highlights a region where secondary structural elements are quite divergent across models, especially between monomeric (first three sequences) and hexameric (bottom sequence) NMR-based models. h, helix; c, coil; t, turn. The sequence of the p7 (J6) used in this study is also shown for comparison. An amino acid similarity index is used where *asterisk* indicates invariant, *colon*, highly similar and *dot*, similar. **B)** Comparison of the NMR model in DPC [47] and NMR/MD model in POPC [44] showing the hexameric form and two opposing subunits in the hexamer. Lines shown in the left hand panels represent the membrane interfaces and hydrophobic core (between the middle two lines). The positions of both models relative to the membrane bilayer were deduced from MD simulations in a POPC bilayer as previously reported for model 1 [53] and model 2 [44]. N- and C-termini are noted by “N” and “C”, respectively. **C)** N-terminal helical packing in both models demonstrates similar packing and residues 9 and 12 in both models point towards the pore. Figures were generated from structure coordinates by using VMD (http://www.ks.uiuc.edu/Research/vmd/ [54]) and rendered with POV-Ray (http://www.povray.org/).

The first structure of monomeric p7 was obtained by combining NMR experiments performed in a 2,2,2-trifluoroethanol (TFE) / water mixture with molecular dynamics (MD) simulations [41]. A full-length, FLAG-tagged monomeric p7 structure was later determined in methanol [42], and recently, structures of p7 from two different genotypes were determined in 1,2-Dihexanoyl-*sn*-glycero-3-phosphocholine (DHPC) micelles [22] and dodecylphosphocholine (DPC) [47], illustrating the monomeric and hexameric p7 forms, respectively. Importantly, these p7 NMR structure models differ on the location of secondary structural elements and orientation of p7 transmembrane domain regions (**Fig 2A**), most notably for segment 33–47. These discrepancies may be due in part to differences in the HCV genotype tested (1b vs. 5a) and/or the lipid-mimicking environment used (TFE, DHPC, DPC, or methanol), the latter of which has been shown to impact p7 activity [46]. To better visualize reported p7 structural elements, we used hexameric p7 models in DPC as described by OuYang and colleagues [47] (model 1; **Fig 2B**) and in 1-palmitoyl-2-oleoyl-sn-glycero-3-phosphocholine (POPC) as described by Chandler et al. [44] that employs the monomeric p7 structure put forth by Montserret et al. [41] (model 2; **Fig 2B**). These two models were selected based on the availability of hexamer structure coordinates and our effort to compare divergent models. Interestingly, in model 1, the p7 subunits are crossed such that part of each monomer interacts with all other subunits, while in model 2, they reside side-by-side, illustrating the topology typical of two transmembrane helical proteins (**Fig 2B**). However, despite the huge differences in the organization of the central part of p7 subunits between these two models, the N-terminal helix (1–18) is close to the C-terminus segment of p7 subunits in both models and forms a hexameric helix bundle with a similar organization of residues; notably, the side chains of amino acids 9 and 12 in both models point to the pore lumen (**Fig 2C**). Importantly, this organization is also observed in other theoretical and NMR-based models [40,42].

### Mutagenesis of the p7 N-terminus is deleterious for infectious HCV production

**Fig 3:**
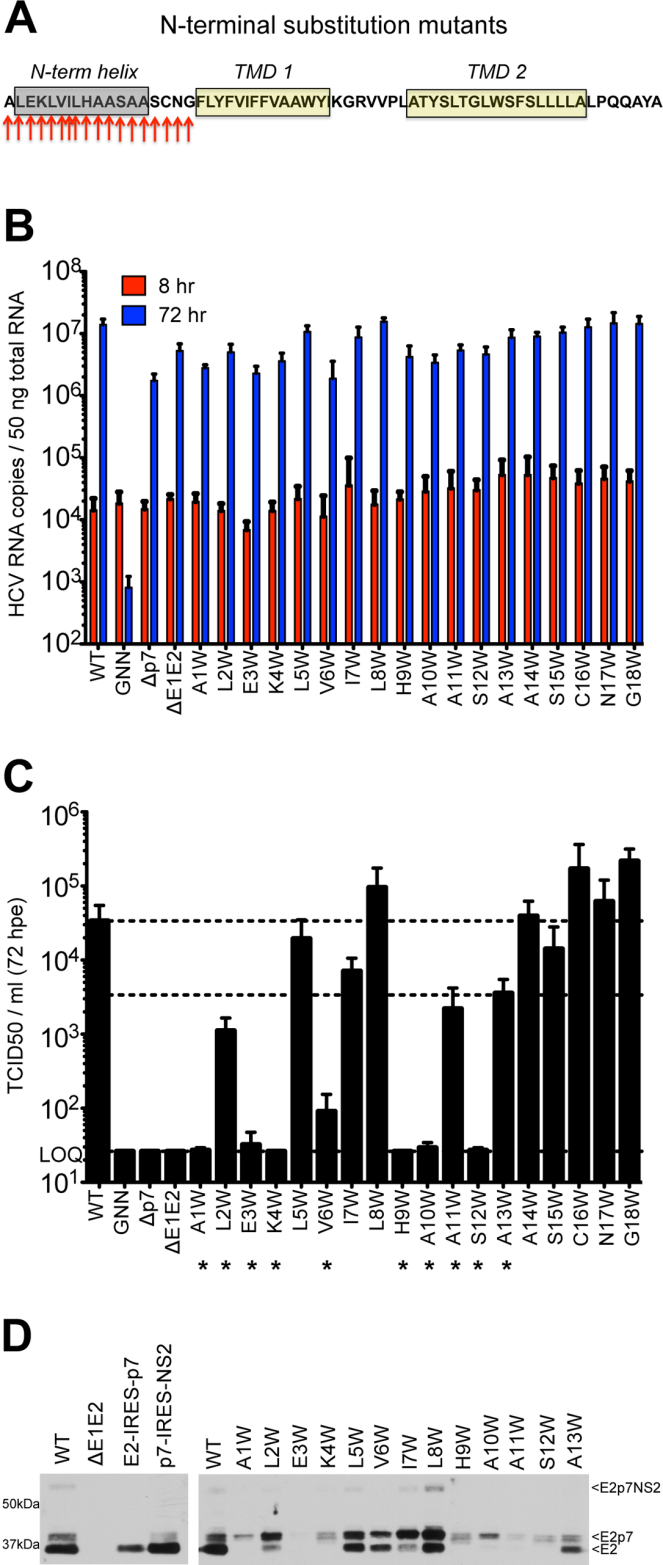
Mutation of the N-terminal helix is deleterious for infectious virus production *in vitro*. **A)** Cartoon of the N-terminal tryptophan substitutions generated in J6/JFH p7. As in Fig 1, the secondary structure boundaries shown were previously deduced from p7 NMR data using HCV-J (genotype 1b) in 50% TFE [41]. **B)** HCV RNA levels in Huh-7.5 cells 8 and 72 hpe demonstrating that all p7 mutants replicate efficiently. J6/JFH lacking either p7 (Δp7) or the HCV glycoproteins (ΔE1E2) were used as additional assembly-defective controls. **C)** Infectious virus production quantified by limiting dilution assay on naïve Huh-7.5 cells shows that mutation of various residues in this region preclude generation of infectious HCV particles. **D)** Western blot analyses detecting HCV E2 antigen. Left panel: J6/JFH WT-, ΔE1E2-, E2-IRES-p7-, or p7-IRES-NS2-replicating Huh-7.5 cell lysates, used here to provide markers for E2p7NS2, E2p7, and E2 protein species. Right panel: Parallel western blot analysis comparing amounts of E2 relative to E2p7 between J6/JFH WT and N-terminal helix p7 mutants (positions 1–13).

Given these similarities in N-terminal helical packing and the conserved hydropathic pattern in this region, we extended our tryptophan mutagenesis of p7 to screen positions 1–18 (**Fig 3A**). Similar to the mutants tested in Fig 1, all of the N-terminal mutants replicated to similar wild-type levels (**Fig 3B**), yet strikingly, mutagenesis in this region had a more profound impact on infectious virus production illustrated by a greater than 1 log decrease in titer for half of the mutants tested including A1W, L2W, E3W, K4W, V6W, H9W, A10W, A11W, and S12W. While varying levels of infectious HCV were detected for genomes harboring mutations at positions 5, 7, 8, 11, and 13, six of the viruses tested (A1W, E3W, K4W, H9W, A10W, and S12W) failed to produce any detectible virus by 72 hours post-electroporation (**Fig 3C**).

Previous studies have shown that mutations in the N-terminal region of p7 can modulate the partial cleavage at the E2/p7 and p7/NS2 junctions [55]. To assess a potential impact of Trp substitution on host signal peptidase cleavage efficiency, we probed for E2 antibody-reactive proteins in Huh-7.5 cells replicating WT or p7 mutant genomes by western blot. To demonstrate E2 antibody specificity and distinguish between incompletely processed E2-containing protein species (E2p7NS2, E2p7, and E2), we utilized monocistronic, wild-type J6/JFH1 and ΔE1E2 genomes along with bicistronic genomes that contain an IRES between E2 and p7 or p7 and NS2 to remove the requirement for protein processing at these junctions. Parallel analysis of N-terminal helix mutants illustrated an E2/p7 processing defect for A1W, E3W, and K4W. Surprisingly, these data also suggested a similar defect may contribute to the deleterious phenotypes of other downstream N-terminal helix mutants, most notably for H9W through S12W (**Fig 3D**).

### Passage of defective N-terminal mutants reveals compensatory mutations

**Fig 4:**
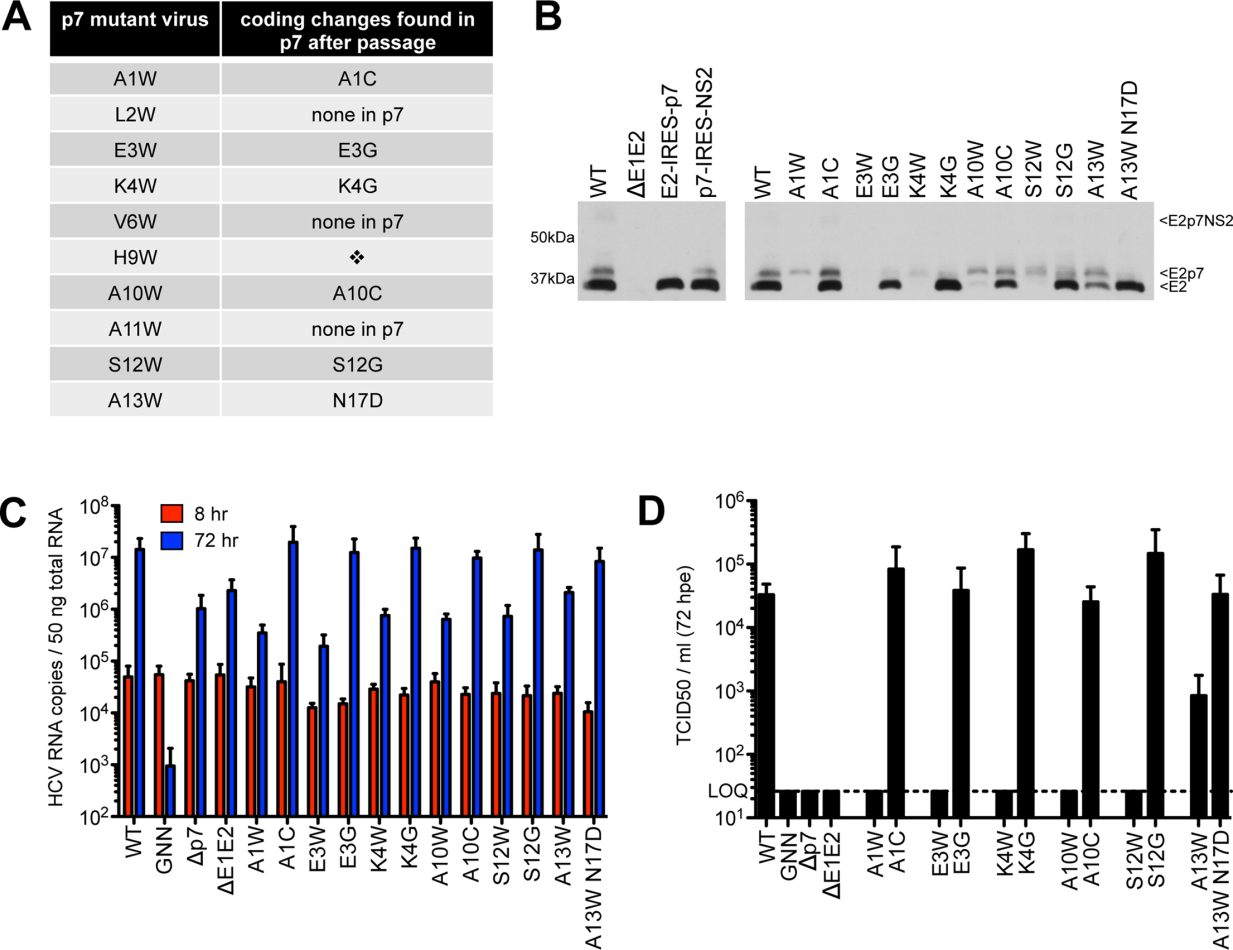
Passage of deleterious N-terminal mutants identifies second site pseudo-revertants. **A)** Coding changes identified within p7 by sequencing after passage of original p7 mutant genomes in Huh-7.5 cells. No virus was obtained after passage of H9W (indicated by the diamond). **B)** Western blot analyses detecting HCV E2 antigen. Left panel: J6/JFH WT-, ΔE1E2-, E2-IRES-p7- or p7-IRES-NS2-replicating Huh-7.5 cell lysates, used here to provide markers for E2p7NS2, E2p7, and E2 protein species. Right panel: Parallel western blot analysis comparing amounts of E2 relative to E2p7 between J6/JFH WT, original p7 mutant genomes and genomes harboring mutations identified after passage. **C)** HCV RNA levels in Huh-7.5 cells determined 8 and 72 hpe showing all p7 mutants (original and those harboring mutations identified after passage re-engineered into J6/JFH p7) replicate efficiently. **D)** Infectious virus in the supernatants quantified by limiting dilution assay on naïve Huh-7.5 cells demonstrating rescue of infectivity by mutations identified after passage re-engineered into the J6/JFH backbone.

To identify second site amino acid changes that could compensate for E2/p7 cleavage or p7-specific defects introduced by tryptophan substitutions, we serially passaged Huh-7.5 cells harboring deleterious N-terminal mutant genomes to allow for the emergence of variants that are competent for infectious virus production (**Fig 4**). After three to seven passages, virus was detected in the supernatant for all genomes except H9W. Despite several attempts at electroporating hepatoma cells with this mutant genome, we were unable to select for a virus capable of spread. This was not due to a high genetic barrier (i.e. the requirement of multiple nucleotide changes to obtain a viable virus), as both serine and glycine (amino acids that are one nucleotide change away from tryptophan) function in this position (**Fig 7; see also S1 Fig and S6 Fig for models**). Further analysis by titrating WT RNA into a constant amount of either control (Δp7) or H9W RNA at the time of electroporation resulted in 3.7-fold less infectious virus production at a WT:H9W RNA ratio of 1:2 (compared to WT:Δp7 at the same ratio). While these data hinted that the mutant p7 might act via a dominant negative mechanism to suppress WT p7 and the production of infectious virus (**S2 Fig**), the effect was less dramatic when the ratio was further increased in favor of the mutant.

We next sequenced the p7 region of HCV RNA extracted from naïve Huh-7.5 cells inoculated with supernatant from passaged cells replicating mutant HCV genomes and identified conservative same-site changes in five of the ten viruses analyzed (**Fig 4A).** These viruses–with mutations at positions 1, 3, 4, 10, and 12 –failed to produce any detectable infectious virus in our original characterization, and together, these data suggest that certain physico-chemical characteristics of the amino acid side chain at these positions are critical. Replacement of tryptophan at positions 1 and 10 with cysteine represents a reversion to a small residue, while glycine at polar positions 3, 4, and 12 likely represents a release of hydrophobic steric constrains (**S3 Fig**). Re-engineering of these amino acid changes into the original mutant genome confirmed their ability to rescue WT-levels of virus production (**Fig 4C and 4D**). L2W, V6W, and A11W revealed putative second-site mutations in E2; however, as we chose to focus our analysis on p7, whether these mutations are responsible for rescuing infectious virus production remains to be determined.

To investigate whether rescue of infectious virus production correlated with enhanced E2/p7 cleavage, we probed for E2 by western blot, comparing original mutant genomes with those re-engineered to contain p7 mutations identified after passage. Strikingly, all viruses harboring p7 mutations identified after passage yielded a marked increase in the amount of ‘free’ E2 relative to E2p7 compared to their original mutation counterparts (**Fig 4B**). These data suggest that diminished cleavage at this junction contributed to our original deleterious phenotypes for these N-terminal helix mutant viruses and consequently impede our ability to evaluate their impact with respect to p7-specific functions.

### Mutagenesis of the p7 N-terminal helix in a bicistronic genome reveals p7-specific defects

**Fig 5:**
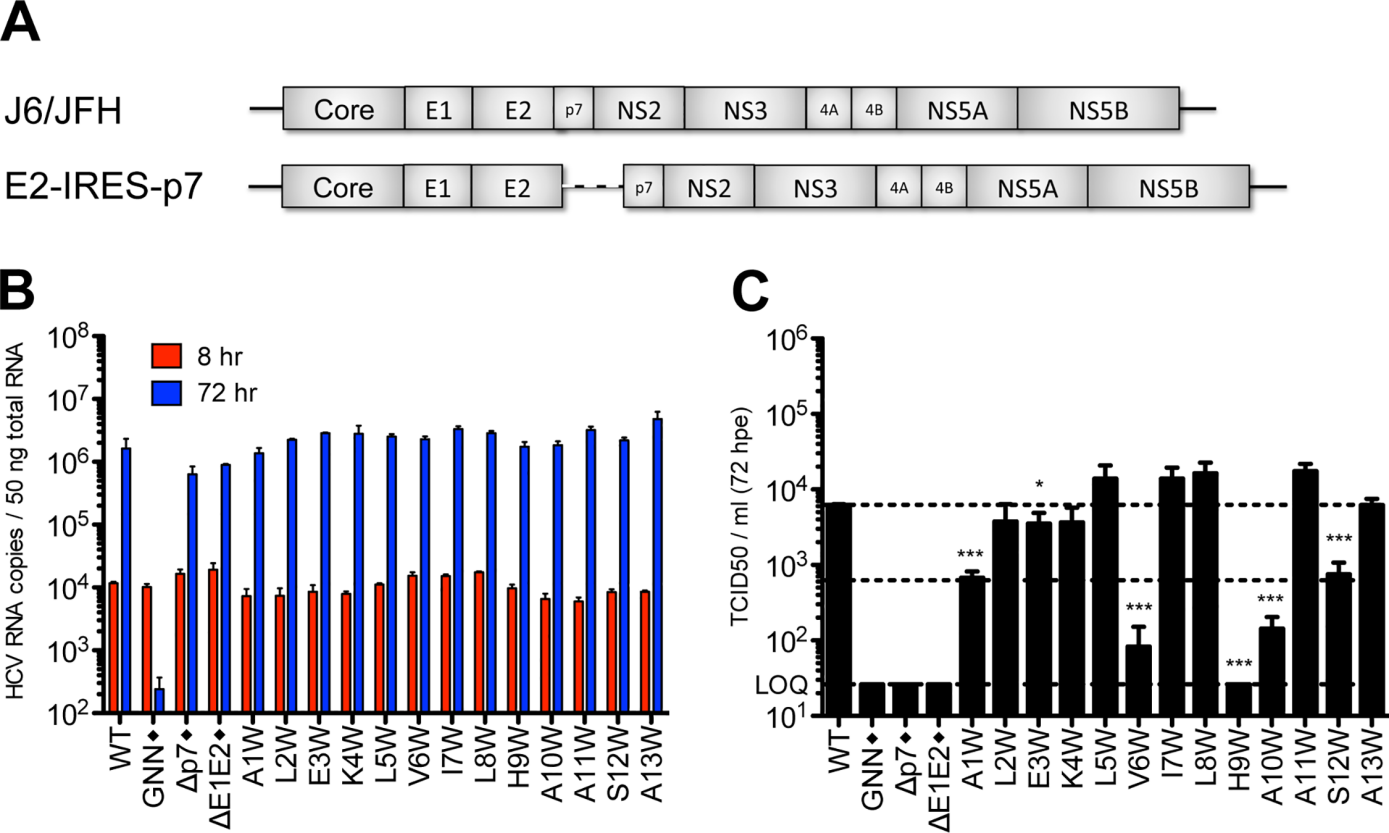
Analysis of N-terminal helix tryptophan substitutions in a bicistronic context. **A)** Schematic showing the full-length monocistronic and bicistronic (E2-IRES-p7) J6/JFH genomes. **B)** HCV RNA levels in Huh-7.5 cells 8 and 72 hpe demonstrating that all bicistronic p7 mutants replicate efficiently. **C)** Infectious virus production quantified by limiting dilution assay on naïve Huh-7.5 cells. Mutant viruses yielding significantly less infectious virus as compared with wild-type were identified using unpaired t-tests. Statistical results are indicated as follows: * p<0.05 and *** p<0.0001. Dashed lines show the mean wild-type titer (upper line) and a 1 log decrease in titer from wild-type (lower line). Black diamond (◆) indicates that these control viruses are monocistronic (see GNN, Δp7, and ΔE1E2 in panels b and c).

To evaluate the impact of tryptophan substitutions in the N-terminal helix independent of E2/p7 cleavage, we engineered these mutations at positions 1–13 into a bicistronic genome containing the EMCV IRES between E2 and p7 (J6/JFH E2-IRES-p7 [11]; **Fig 5A**), thus eliminating the need for polyprotein processing at this junction. We then phenotyped these bicistronic N-terminal tryptophan substitution mutants with respect to replication and infectious virus production after electroporation into Huh-7.5 cells. As expected, all mutant viruses replicated to similar wild-type levels (**Fig 5B**). Furthermore, as predicted by our western blot data indicating defects in E2/p7 cleavage were at least partially responsible for abrogation of infectious virus production, the majority of bicistronic N-terminal helix mutants now yielded infectious virus titers that were comparable to wild-type (**Fig 5C**). Nonetheless, several mutants, including A1W, V6W, H9W, A10W and S12W, remained impaired, suggesting a cleavage-independent, p7-specific defect also impacts infectious particle production for these viruses.

### Homology modeling provides insight into p7-specific functional consequences of tryptophan mutagenesis

**Fig 6:**
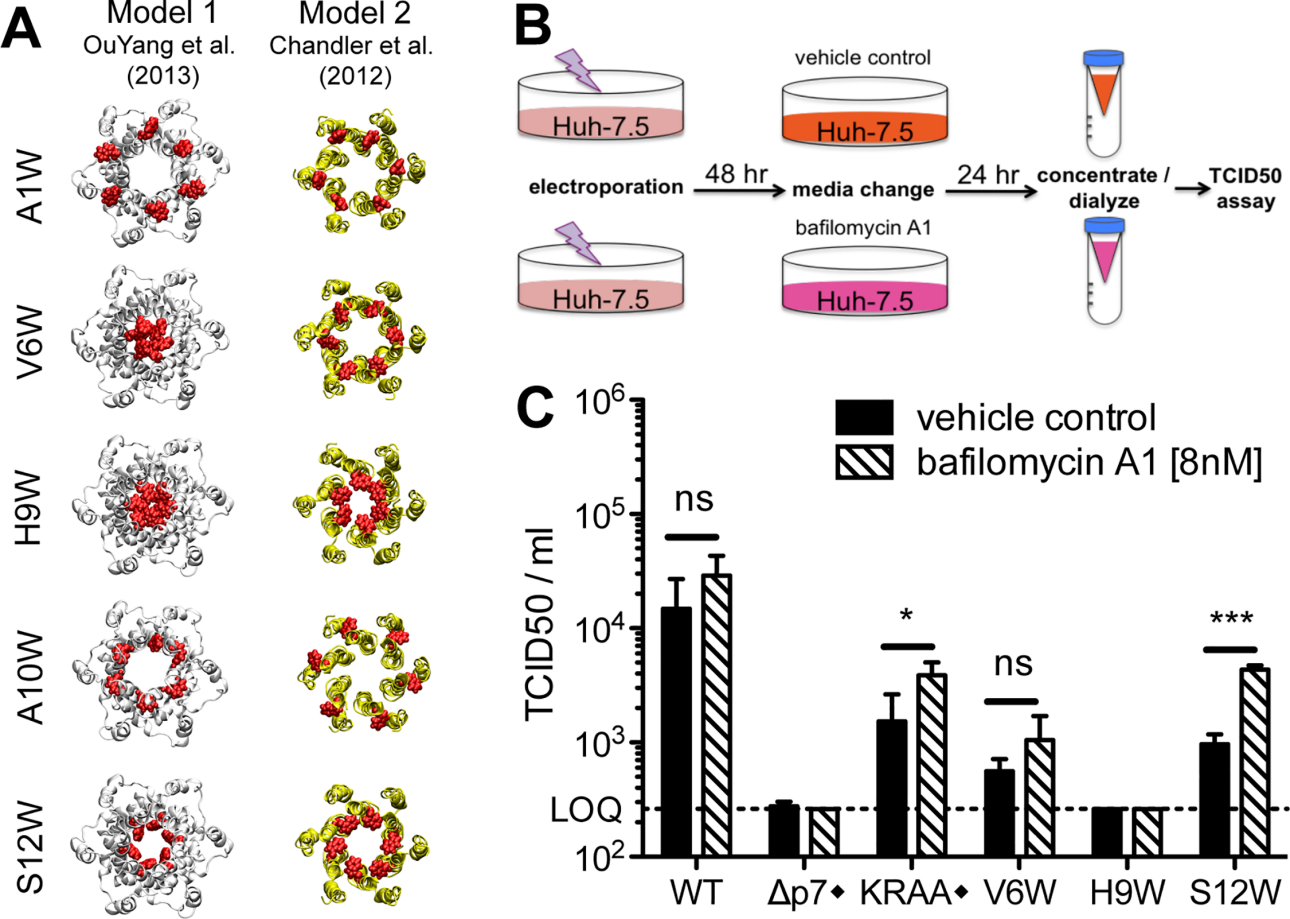
Identification of putative ion channel defective mutants by homology modeling and bafilomycin A1 rescue. **A)** Molecular models of N-terminal region mutants that yielded >1 log reduction in infectious virus production compared to wild-type in a bicistronic context. The mutated residue Trp side chains are shown in red. Models provide insight into whether the mutation is likely to block the pore (e.g. H9W and S12W), disturb p7 intramolecular interactions (e.g. A10W), or interrupt p7 interactions with binding partners (e.g. A1W, A10W). **B)** Bafilomycin A1 rescue experiment schematic. Forty-eight hours post-electroporation, Huh-7.5 cells replicating control or p7 mutant viruses were supplied with cell culture medium containing bafilomycin A1 [8nM] or DMSO. Supernatants were collected 24 hours post-treatment, concentrated and dialyzed to remove excess bafilomycin A1, and then tittered on naïve Huh-7.5 cells to quantify infectious virus production. **C)** Resulting infectious virus titers from the experiment outlined in panel b. Mutant viruses yielding significantly more infectious virus production under bafilomycin A1 conditions compared with DMSO were identified using unpaired t-tests. Statistical results are indicated as follows: ns = not significant, * p<0.05, and *** p<0.0001. LOQ: lower limit of the limiting dilution assay. Black diamond (◆) indicates that these control viruses are monocistronic; all others shown are bicistronic. KRAA denotes J6/JFH with K33A and R35A mutations in p7.

These phenotypes indicated a deleterious impact of tryptophan on p7 function at positions 1, 6, 9, 10 and 12 but do not provide evidence for a rational hypothesis regarding the mechanism of the defect. Thus, to gain insight into the impact of these mutations on p7 structure, we modeled these tryptophan substitutions via homology molecular modeling. Because the structural impact, and hence, proposed functional consequence of our mutations, may differ depending on the 3D model, we aimed to develop hypotheses based on both model 1 and model 2 (**Fig 2B**). Comparing the sequence of the J6 (genotype 2a) p7 used in our study to the genotype 1b and 5a p7 used to study the p7 structure by NMR indicated sufficient similarity at the amino acid level (**Fig 2A**) to enable the generation of J6 p7 models by homology using Swiss-Model facilities [56]. (Coordinates of homology models 1 and 2 for p7 HCV J6 strain are available as supplementary.pdb files; **S1 File and S2 File**). Introduction of any of our tryptophan mutations in these homology models yielded energetically stable hexamer structures without significant structural changes indicating that p7 structure models 1 and 2 readily accommodate these mutations (**S1 Fig**). We then closely examined each model to evaluate/predict the structural/functional consequence of the tryptophan substitution (**Fig 6A**). Not surprisingly, given the similarity of these two models at the N-terminus (**Fig 2**), our predictions were generally consistent between model 1 and model 2 (**S1 Fig**). Indeed, the orientation of the tryptophan side chain toward the lumen of the pore in both models suggests a likely ion channel defect for H9W and S12W. The A10W mutation could disturb p7 intramolecular interactions or interrupt p7 interactions with binding partners, as also predicted for the A1W mutant because of its N-terminal position (**S1 Fig**). Interestingly, model 1 and model 2 did give rise to incongruent hypotheses for some mutants (e.g. V6W), suggesting these residues may impact multiple aspects of p7 function. Alternatively, such mutants could be used as tools to test the accuracy of one model over the other by directly assessing the functional defect in cell culture.

### Bafilomycin A1 treatment mediates a partial rescue of the p7 S12W bicistronic mutant

We next sought to corroborate the hypothesized p7 functional consequences of tryptophan substitution based on our homology models by further assessing selected mutants in cell culture. Specifically, we aimed to rescue infectious virus production by putative ion channel defective mutants in Huh-7.5 cells using bafilomycin A1. Bafilomycin A1 prevents vesicular acidification and thus may compensate for a loss of p7 channel activity. Further, this inhibitor was previously shown to compensate for a defective p7 mutant harboring mutations K33A and R35A [36]. In our initial experiments, 8 nM bafilomycin was found to be both relatively non-toxic to cells and extremely effective in alkalinizing cellular compartments and over a 24 hr time period, retaining 80% cellular viability with complete loss of acidic organelle labeling with LysoTracker Red DND-99 (**S4 Fig**). Since bafilomycin A1 can also prevent endosomal acidification and thus impede HCV entry into cells used for subsequent infectivity analysis, we further optimized methods to concentrate virus- and bafilomycin A1-containing supernatants 5-fold while simultaneously removing a sufficient amount of the inhibitor to enable infectious virus quantification by limiting dilution assay (**S4 Fig**).

We selected V6W, H9W, and S12W bicistronic p7 mutant viruses specifically for analysis based on our homology models that suggested an ion channel defect for both H9W and S12W. For V6W, the interpretation differed between model 1 and model 2, providing a potential opportunity to decipher between them. Following electroporation and incubation of Huh-7.5 cells with selected viral genomes, cells were provided with media containing either bafilomycin A1 or DMSO. Cell culture supernatants were collected 24 hours later and infectivity was assessed (**Fig 6B**). Notably, bafilomycin A1 treatment resulted in a boost of viral titers for all genomes tested that were capable of making detectible levels of infectious particles under DMSO conditions. However, only in the case of the KRAA mutant and our S12W mutant was this increase significant (**Fig 6C**). These data support our structure model-based hypothesis that tryptophan substitution at position 12 abrogates ion channeling, and also indicate that altering intracellular pH via bafilomycin A1 treatment is insufficient to ‘rescue’ the impact of tryptophan substitution at position 6. Interestingly, despite both models pointing to an ion channel defect for H9W, we were unable to recover any infectious virus for this mutant in our assay. One explanation is that our methods are not conducive to detection of low levels of infectious virus production; indeed, the limit of quantification for our limiting dilution assay in this context is 10-fold higher than previous experiments due to some residue bafilomycin A1 carryover in the supernatant. Thus, a small, but significant increase in infectious virus production, as was previously shown in a similar experiment with the KRAA mutant [36], may not be uncovered.

### Amino acid requirement analysis at pore-lining residues

**Fig 7:**
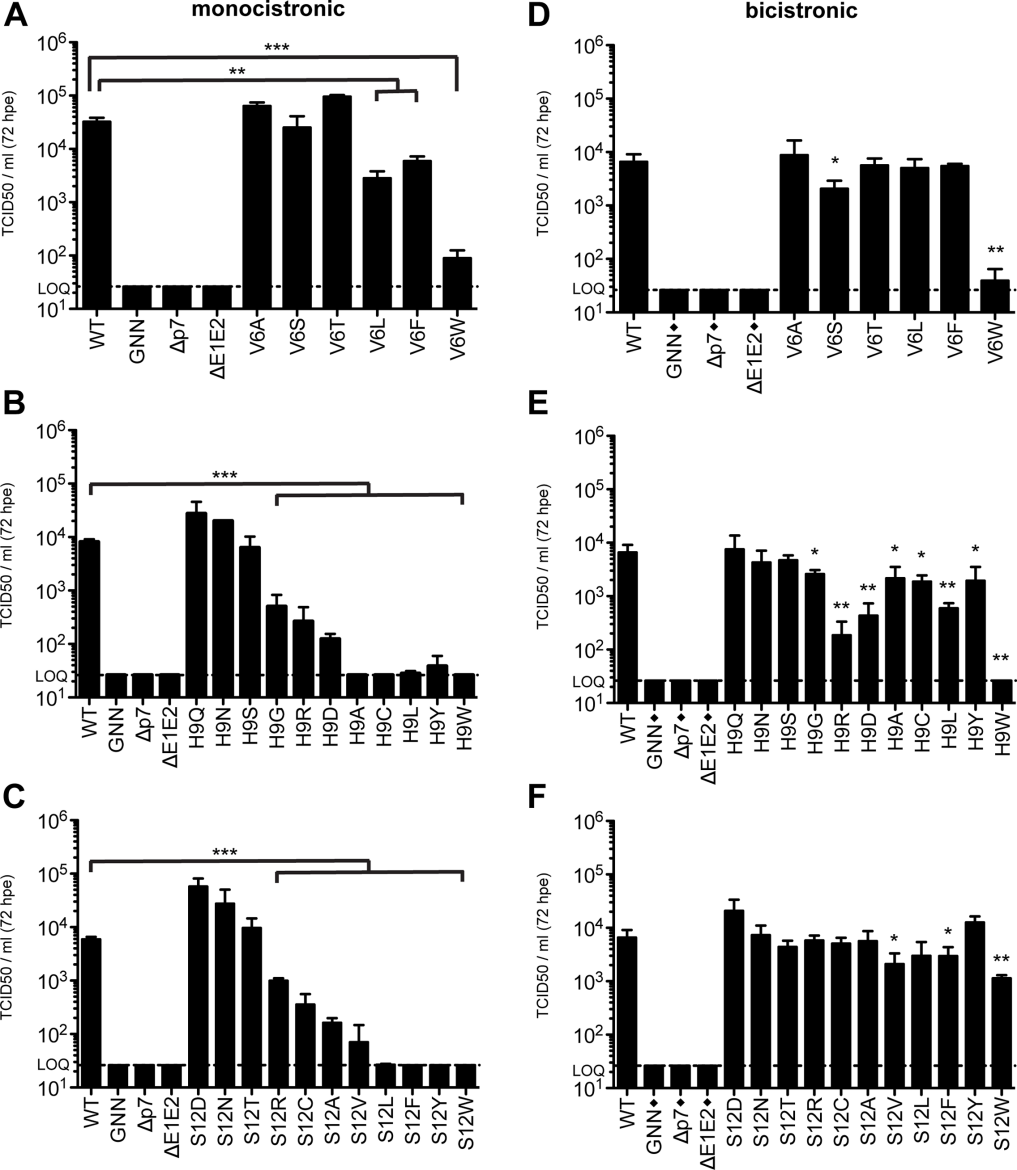
Amino acid requirements at positions 6, 9, and 12. Infectious virus in the supernatants of monocistronic **(A, B & C)** or bicistronic **(D, E & F)** p7 mutants quantified by limiting dilution assay on naïve Huh-7.5 cells. Mutant viruses yielding significantly less infectious virus production as compared with wild-type were identified using unpaired t-tests. Statistical results are indicated as follows: * p<0.05, ** p<0.01 and *** p<0.001. Black diamond (◆) indicates that these control viruses are monocistronic. Note that the WT, GNN, Δp7, and ΔE1E2 titers reported in panels **E** and **F** are duplicated from panel **D** as these viruses were all analyzed in parallel.

In both models interrogated in this study, residues 9 and 12 point to the pore formed by p7 oligomerization (**Fig 2C**). These structural data, supported by our ability to significantly increase S12W mutant infectious viral titers by altering vesicular pH, indicate the amino acids at these positions could contribute to cation selectivity and flux. To further analyze the requirements at these residues, as well the residue at position 6, which is oriented towards the pore in model 1, we expanded the amino acid repertoire at these positions and analyzed the impact of polarity, charge, and hydrophobicity on viral replication and infectious virus production (**Fig 7**). Amino acids were analyzed in both monocistronic and bicistronic viral genetic backgrounds in order to segregate between amino acids that impact E2/p7 cleavage versus those that influence p7-specific functions. Our data indicate that although position 6 tolerates both hydrophilic and hydrophobic residues, bulky residues (Leu, Phe and Trp) are more detrimental in the monocistronic context, suggesting these amino acids have a negative impact on E2/p7 cleavage, whereas residues with smaller side chains (Ala, Ser, Thr) have almost no effect on infectious virus production (**Fig 7A and 7D, and S5 Fig**). Specifically, we observed a correlation between the increasing size of hydrophobic residue side chains (**S6 Fig**) and the inhibition of virus production.

Extending our analysis to position 9, we observed that polar amino acids Gln, Asn, and Ser all function at this position to support infectious virus production while hydrophobic residues (Ala, Cys, Leu and Tyr) do not (**Fig 7B**). In agreement with these data, H9A mutation in JFH-1 p7 (genotype 2a) was previously shown to reduce channel conductance by ~70% [47]. However, the impact of these hydrophobic residues was less significant in Huh-7.5 cells replicating bicistronic genomes, indicating the primary impact of these substitutions is on E2/p7 cleavage (**Fig 7E**). Surprisingly, both acidic (Asp) and basic (Arg) residues support infectious virus production, albeit to low levels.

Similar to position 9, we also observed that amino acids at position 12 with bulky hydrophobic side chains inhibited virus production in the monocistronic context, but this was again less apparent for bicistronic genomes harboring the same amino acid changes (**Fig 7C and 7F**), indicating again that the primary impact of these substitutions is on E2/p7 cleavage. In both cases, amino acids at positions 9 and 12 with polar character supported infectious virus production (**Fig 7B, 7C, 7E and 7F**). Interestingly, substitution with negatively charged Asp at position 12 yielded an increase in viral titers above those obtained for WT, potentially via enhanced cation recruitment at the pore entry. However, positively charged Arg also functions at this position while Ala and Ser are the only natural amino acids found at this position. Together these data indicate that E2/p7 cleavage efficiency is sensitive to downstream mutations within the N-terminal helix region of p7 while the tolerance of positions 6, 9, and 12 to amino acids of different nature in a bicistronic context further supports p7 as a structurally plastic, minimalist ion channel.

## Discussion

In this report, we have extensively interrogated the HCV p7 protein via mutagenesis and determined the effects of these mutations on virus replication and infection in cell culture. In addition, we have modeled these mutations using p7 structure information based on previous NMR experiments. Our data confirm previous reports that p7 is not required for viral replication, as all p7 mutants tested replicated with wild-type efficiency. Importantly, our large-scale, structure-function analyses illustrate a global tolerance for amino acid sequence alterations, either by insertion or individual amino acid substitution in the J6/JFH background. These results underscore the structural flexibility of p7 [44] that has been similarly described for other viroporins such as HIV-1 Vpu [57]. Our data are further in line with the conservation of p7 amino acid physico-chemical properties and hydropathic character but not precise sequence across genotypes [41]. Notably, two of the nine conserved amino acids (G18 and Y42) were directly assessed in this study by Trp substitution and resulted in an increase and decrease in infectious virus titer, respectively, although these phenotypes were not the most dramatic in our panel. Interestingly, structure models give rise to incongruent hypotheses regarding the impact of G18W mutation (**S1 Fig**), suggesting this residue may provide another opportunity to further probe these structure models and test p7 function in cell culture and p7 ion channel activity after reconstitution in artificial membranes. Together our data indicate that escape mutants with significant fitness could be readily generated in the context of p7-targeting antiviral compounds, potentially limiting the efficacy of this class of inhibitors in the clinic. Still, there were several positions tested that did show a marked impact on infectious virus production; this was most pronounced when the residues within the first eighteen amino acids, comprising the N-terminal helical region, were interrogated.

Our functional predictions are based on available hexameric p7 models; however, previous studies indicate that the oligomerization of seven p7 subunits is also feasible [35,44], possibly even resulting in a mix of oligomeric states within the infected cell. Nonetheless, computational analyses for models where p7 subunits reside side-by-side [35,44] indicate that amino acid positions are similar and pore lining residues are retained with the addition of the 7^th^ monomer; thus, our data interpretation would likely be consistent in this context. Interestingly, molecular dynamics simulations in a hydrated POPC bilayer showed that hexameric p7 model 2 formed a pore that was transiently permissive to solvent, potentially linked to a hydrophobic barrier formed by F25 [44]. In our initial mutant panels we observed a decrease in titer following Trp substitution at position F26 and an increase in titer after F25W mutation. Both hexameric models assayed here suggest residue 26 has multiple contacts within p7, thus likely playing a role in stabilizing the protein, whereas the amino acid at position 25 lines the pore. Naturally occurring residues at both of these positions across genotypes are invariably hydrophobic, and while the higher polarity of the Trp side chain may facilitate the passage of ions at position 25, resulting in a higher production of virus particles, the same substitution at position 26 may destabilize p7 assembly. The ability of Trp substitution to boost titers at several positions (including residue 25, as well as 29 and 31) is interesting given that this amino acid does not naturally occur at any of these positions. This suggests that the increased titers we observed in Huh-7.5 cells are not advantageous in a more physiologically relevant system (e.g. primary human hepatocytes) and may negatively impact viral fitness *in vivo*, although this was not directly tested in this report.

A side-by-side structural comparison of the models proposed by OuYang et al. [47] (model 1) and Chandler et al. [44] (model 2) revealed similar helical packing at the N-terminus in these otherwise incongruent models; hence, we focused our studies on this region of p7. Surprisingly, several lines of investigation, including western blot analyses of N-terminal helix mutants and related pseudorevertants, as well as infectious virus production phenotyping of bicistronic mutant genomes, all indicated that mutation within this region has a significant, detrimental impact on E2/p7 processing. This supports previous studies that have implicated this region in modulating the partial cleavage at E2/p7 and p7/NS2 junctions [58]. Interestingly, A13W, a mutant that originally demonstrated a 1-log attenuation compared to WT, acquired an additional mutation at position 17 (N17D) after passage that correlated with enhanced E2/p7 processing and increase in infectious virus production. In model 1, residue 13 points towards the pore, while 17 lies at the p7 protein surface within the hydrophobic region of the membrane–making the identification of negatively charged aspartic acid at this position energetically counter intuitive (**S3 Fig**). In model 2 (as well as the model presented by Foster et al. [42]), however, residues 13 and 17 both point to the p7 pore, one directly above the other, indicating these residues could be related in function.

Overall, our work suggests that tryptophan substitution (or potentially bulky or hydrophobic residues in general, as demonstrated for residues 6, 9, and 12) negatively modulates important interactions between the C-terminus of E2 and the N-terminus of p7 that play a regulatory role in cleavage efficiency of the E2/p7 junction, possibly through mediating correct presentation of the cleavage site to the signal peptidase. In uncleaved E2p7 species, the topology of p7 may be inverted [59], and while a specific role for E2p7 in the HCV life-cycle remains questionable, it has been hypothesized that the proper timing of E2/p7 cleavage may be critical to avoid spontaneous ion channel formation in the ER membrane and to promote the assembly process [44]. Nonetheless, the separation of E2 and p7 is absolutely required for infectious virus production, specifically for proper NS2 localization near assembly complexes [58] and presumably p7 oligomerization.

Beyond deficiencies in protein processing, our mutagenesis data using bicistronic constructs, further informed by homology modeling, identified several N-terminal helix mutants with p7-specific defects, including positions 6, 9, and 12. Interestingly, modeling of V6W (**Fig 6A**), which is located within a conserved hydrophobic cluster spanning from position 5–8 and is either a Val or Ile in all HCV genotypes, indicated this mutation would face the pore in model 1 and has been proposed to play a role in closing the pore via the formation of a hydrophobic ring [47]. In contrast, this V6W mutation would likely affect helix-helix interactions (i.e. oligomeric structural stability) in model 2. In fact, our inability to rescue this bicistronic mutant using bafilomycin A1, in addition to the fact that position 6 tolerates both hydrophilic and hydrophobic residues suggests this position does not play a critical direct role in ion channeling.

Position 9 is an Asn or, in genotype 2 viruses, a His–both of which have an affinity for monovalent and divalent cations. The hydrophilic nature of this position, as well as its location at the pore entry in both models, has implicated this residue in cation selectivity [41]. OuYang et al. [47] further propose it serves as a “filter” to dehydrate cations, allowing them to pass through the hydrophobic ring formed by position 6, the more narrow part of the channel in model 1. In accordance with these hypotheses, polar residues supported virus production in our study, while substitution with hydrophobic or charged residues resulted in significantly decreased infectious virus titers compared to wild-type. Our results for this residue were reminiscent of those obtained for position 17, which is located within the turn sequence and found to line the pore. Position 17 naturally occurs as a histidine or asparagine (like position 9) as well as glutamine–all of which share polar characteristics–and has been implicated in ion channel function [49], as our present data suggest for position 9. Similar to our results at position 9, mutation of H17 to A or G by Meshkat et al. resulted in a decrease in titer while H17E (polar) boosted infectious virus in the supernatant [51]. Interestingly, we did not see a major phenotype following Trp substitution at position 17 (N17W) in the context of the J6 p7 sequence, perhaps due to conservation of some polarity by this substitution.

Beyond positions 6 and 9, we also investigated the previously overlooked residue at position 12, which is only Ser or Ala in natural variants and points to the pore in both models. Our data show a striking complete loss of infectious virus production upon Trp substitution–a phenotype that was only partially rescued when this mutation was subsequently engineered into a bicistronic background. Modeling data indicated aromatic ring-mediated pore obstruction while subsequent rescue with bafilomycin A1 further suggest a novel role for this position in ion channel function. Still, the global tolerance of both position 9 and position 12 for amino acid substitution of different characteristics supports p7 as a structurally plastic, minimalist ion channel.

Our present study identified N-terminal mutants that are defective for infectious virus production but did not distinguish between a defect in particle assembly versus infectivity. The identification of p7 mutants that are competent for particle assembly, but exhibit a profound defect in specific infectivity would provide a unique tool to probe the impact of p7 function on the viral particle at later stages of the viral life cycle. Further, deleterious mutants for which our models generated incongruent functional defect hypotheses offer additional opportunities to delineate between these models by probing p7 functional defects in cell culture or reconstituted in artificial membranes and correlating these with structure-based predictions to support or refute the available structural data. Importantly, our study highlights a potential regulatory role of the p7 N-terminal helix residues in the cleavage efficiency of the E2/p7 junction, although the precise underlying mechanisms remain elusive. In sum, our work illustrates the convergence of current p7 models at the N-terminal helix and demonstrates the biological impact of amino acid perturbation in this region, offering extensive insight into the relationship between p7 structure and function in the context of HCVcc.

## Materials and Methods

### Plasmid construction

J6/JFH [60], the K33A/R35A p7 mutant and bicistronic genomes J6/JFH E2-IRES-p7 and p7-IRES-NS2 [11] have all been previously described. To facilitate the generation of p7 mutant viruses, two silent restriction sites (NotI and BglII) were engineered into the wild type monocistronic J6/JFH sequence in E2 and NS2, respectively. The resulting genome is termed J6/JFH 1.1 and referred to here simply as J6/JFH or wild-type. Mutations in p7 were introduced by overlap PCR using standard procedures and engineered into monocistronic or bicistronic constructs using NotI and BgIII or MluI and NotI, respectively. Plasmid and primer sequences are available upon request. All constructs were confirmed by sequencing.

### Cell culture

Huh-7.5 cells [61] were propagated in Dulbecco’s modified minimal essential medium (DMEM) supplemented with 10% heat-inactivated fetal bovine serum (FBS) and 0.1 mM nonessential amino acids (NEAA). Cells were grown at 37°C in a humidified 5% CO_2_ atmosphere.

### In vitro RNA synthesis

Viral cDNAs were linearized with XBa1 and purified using a MinElute PCR purification kit (Qiagen). In vitro RNA transcription was performed using a T7 RiboMAX Express large-scale RNA production system (Promega) and newly synthesized RNAs were isolated with an RNeasy RNA isolation kit with a second DNase I digestion (Qiagen) according to the manufacturer’s protocol. RNAs were eluted in nuclease free water and integrity and concentration were determined by agarose gel electrophoresis and absorbance at 260 nm, respectively.

### RNA transfection

In vitro-transcribed RNAs were electroporated into cells using a 4 mm gap 96-well plate format (BTX ElectroSquare Porator ECM830 with Plate Handler; Harvard Apparatus). Briefly, Huh-7.5 cells were trypsinized, washed in cold, RNase-free Dulbecco’s phosphate-buffered saline (D-PBS) without Ca2+ / Mg2+ (Gibco–Invitrogen) and resuspended at a concentration of 1.5 x 10^7^ cells / ml in D-PBS. Two hundred microliters (3 x 10^6^ cells) was then mixed with 5 ug RNA and loaded into the cuvette. Electroporation was performed using the following settings: 0.80 kV, 99 ms, 5 pulses. Pulsed cells were transferred into 1.5 ml DMEM with 10% FBS and 0.1 mM NEAA before plating. Cells were plated in 24-well plates at a density of 5.3 x 10^4^ cells / well in a final volume of 0.5 ml.

### Viral replication and infectious virus production assays

Eight hours post-electroporation, cells were washed twice with D-PBS. One well from each electroporation was then harvested in 0.35 ml RLT buffer containing 0.01 ml beta-mercaptoethanol (βME) per ml, applied to a Qiashredder and spun at 16,300 x g for 2 min before storage at -80°C. A second well from each electroporation was provided with 0.5 ml fresh complete medium and returned to the incubator until 72 hours post-electroporation when the cell culture supernatant was collected and stored at -80°C until analysis. The cells were then washed and collected in RLT buffer as described above. HCV infectious titers in the supernatants were determined by a limiting dilution assay on naïve Huh-7.5 cells as previously described [60]. Total cellular RNA was isolated using an RNeasy kit (Qiagen) and 50 ng of total RNA was then assayed for HCV genomes using a one-step quantitative RT-PCR assay (Multicode-RTx HCV RNA kit, Luminex Corp.) targeting the 3’ UTR of the viral genome and a Roche LC480 light cycler, according to manufacture’s instructions.

### Passage of p7 mutants

Selected p7 genomes shown to be defective for infectious virus production were electroporated into Huh-7.5 cells as described above. Cells were plated (1.3 x 10^6^ cells) in 100 mm dishes and maintained in 10 ml DMEM 10% FBS with 0.1 mM NEAA. Supernatants were collected before each passage and stored at -80°C until analysis. Supernatants found to contain infectious virus were then applied to naïve Huh-7.5 cells (300,000 cells / 100 mm dish plated 24 hrs prior to inoculation), split once, and harvested in 0.6 ml RLT containing βME for RNA extraction and HCV RNA sequencing or fixed in 4% paraformaldehyde (PFA) and stained with anti-NS5A antibody (9E10 [60]-alexafluor 647) to determine the frequency of HCV antigen-positive cells by flow cytometry.

### Molecular modeling

The relatively high amino acid sequence similarities between p7 of HCV strain J6 and that of strains EUH1480 (40% identity, 80% overall similarity) and HC-J4 (62% identity, 92% overall similarity) allowed us to construct three-dimensional homology models 1 and 2 for p7 hexamers, respectively, using the NMR structure of HCV p7 of OuYang et al. [47] as template (PDB accession number 2M6X) for model 1, and the NMR/MD model of Chandler et al. [44] as template for model 2. Models of p7 were constructed with the Swiss-Model automated protein structure homology modeling server (http://www.expasy.org/spdbv/ [56]) using the p7 HCV strain J6 sequence as input. p7 model 1 was directly obtained as a hexamer by the automated procedure. For model 2, raw amino acid sequence of p7 from strain J6 was first loaded in Swiss-PdbViewer software [56] and fitted to the NMR/MD p7 hexamer model of Chandler et al. [44] before submission for model building to Swiss-Model using the SwissModel Project Mode. All p7 mutants were constructed using the latter protocol, i.e., fitting of the raw amino acid sequence of p7 mutants to wild type hexamer models 1 and 2 from the J6 strain. Coordinates of homology models 1 and 2 for p7 HCV J6 strain are available as supplementary.pdb files. These coordinates are derived directly from the automated model building with no further minimization or manual manipulation.

### Western blotting

Electroporated Huh-7.5 cells were plated in 6-well plates and lysed 72 hpe using modified radioimmunoprecipitation assay (RIPA) buffer (50 mM Tris-HCl (pH 8.0), 1% (v/v) nonyl phenoxypolyethoxylethanol, 0.5% (w/v) Na-deoxycholate, 150 mM NaCl, and 0.1% sodium dodecyl sulfate). Protein (10 μg) was then denatured and subsequently deglycosylated using PNGase F according to the manufacturer’s protocol (New England BioLabs, Inc.) before being separated on 4–12% Bis-Tris NuPAGE polyacrylamide gels (ThermoFisher Scientific) and transferred to 0.2 micron nitrocellulose membranes. Membranes were blocked with 5% milk in Tris-buffered saline with 0.1% Tween-20 and E2-containing protein species were detected using rat anti-E2 antibody (clone 3/11 [62]; 2 μg/ml final concentration). Following secondary antibody staining with Peroxidase AffiniPure donkey anti-rat IgG (H+L; 1:10,000), blots were visualized using SuperSignal West Dura reagent (Thermo Scientific).

### Bafilomycin A1 experiments

To establish the bafilomycin concentration to be used in subsequent virus rescue experiments, bafilomycin A1 (Sigma Aldrich; or DMSO vehicle control) was titrated onto mock-electroporated cells and both viability and intracellular pH assessed 24 hrs post-treatment. Cellular viability was determined using CellTiter-Glo luminescent cell viability assay (Promega) according to the manufacturer’s protocol. Parallel wells were washed in HEPES buffer [36] and loaded with 50 nM LysoTracker Red DND-99 (ThermoFisher Scientific) diluted in HEPES buffer for 30 min at 37°C to label acid organelles. Cells were then washed with PBS, trypsinized, and LysoTracker Red content analyzed by flow cytometry after gating on live cell singlets.

For rescue experiments, selected p7 mutant genomes were electroporated into Huh-7.5 cells as described above. Forty-eight hours post-electroporation, the cell culture media was removed and replaced with media containing bafilomycin A1 (8 nM final concentration) or DMSO (vehicle control). Supernatants were harvested 24 hours post-treatment, pooled across identical wells and applied to Millipore centrifugal filters (100 MW cutoff). Samples were centrifuged at 930 x g for 15 min at 4°C and then dialyzed with 4 ml serum free medium by centrifuging again at 930 x g for 12 min to remove bafilomycin A1. The remaining sample volume (250–500 μl) was brought up to 600 μl with DMEM containing 10% FBS and 0.1 mM nonessential amino acids and infectious virus was quantified by standard limiting dilution assay performed on naïve Huh-7.5 cells.

### Statistical analysis

Statistical analysis of virological data was performed with GraphPad Prism 5. Specific tests are noted in figure legends.

## Supporting Information

S1 Fig Location of tryptophan substitutions generated in J6 p7 hexamer structure models 1 and 2 and evaluation of their possible impact on the structure, function, and interaction features of p7. For each tryptophan substitution indicated in the title of each page, p7 top and side views of models 1 and 2 are show in both ribbon and surface representations. Top view (ER lumen view) or bottom view (cytosolic side view) highlights the p7 pore while the side view (membrane side view) highlights the p7 surface embedded in the membrane. Model 1 (Ouyang et al. [47]) and Model 2 (Chandler et al. [44]) are colored white and yellow, respectively. The side-chain atoms of tryptophan residues are represented as red spheres of the corresponding van der Waals radius. The effect of Trp substitution on virus production is indicated as a subtitle and evaluation of its possible effect on p7 structure, function, and/or interaction features is commented at the bottom. Comments in red indicate the convergence of possible effects with both models 1 and 2 while comments in black indicate divergences. Additional comments are in blue.
(PDF)

S2 Fig Impact of H9W on the ability of wild-type HCV to produce infectious particles. Infectious virus in the supernatants of Huh-7.5 cells electroporated with a mix of wild-type RNA and either H9W (2.5 μg) or Δp7 RNA (2.5 μg). The total amount of RNA electroporated across conditions was kept constant (5 μg) by adding additional Δp7 RNA as needed. Infectious virus titers 72 hpe were quantified by limiting dilution assay on naïve Huh-7.5 cells. Data represent the mean and standard deviation of (n = 3) independent electroporations. Statistically significant differences in titers were determined by unpaired t-tests. * p<0.05.
(TIF)

S3 Fig p7 structure models of pseudorevertants identified after passage of defective p7 mutant genomes. **A)** Top view (ER lumen side view) of p7 models 1 and 2 are shown in ribbon representations for both the natural (wild-type) amino acid and amino acid identified at the same position after passage of the Trp substitution mutant in Huh-7.5 cells. The side-chain atoms of natural and pseudorevertant residues are represented as spheres of the corresponding van der Waals radius. Carbon and hydrogen atoms are in cyan, oxygen atoms in red, nitrogen atoms in blue, and sulfur atoms in yellow. **B)** Location of A13W and N17D mutations in the hexameric forms of p7 models 1 and 2 (surface and ribbon representations from different viewpoints) and on two opposing subunits within the hexamers. Lines shown in the left hand panels represent the membrane interfaces and hydrophobic core (between the middle two lines). For more details see the legend for Fig 2.
(TIF)

S4 Fig Optimization of bafilomycin A1 conditions in Huh-7.5 cells. **A)** The effect of bafilomycin A1 on cell viability (CellTiter-Glo assay; Promega) and **B)** acidic intracellular pH indicated by LysoTracker Red DND-99 staining and quantified by flow cytometry. Based on these data, a concentration of 8 nM bafilomycin was used in subsequent assays. **C)** The impact of bafilomycin A1 on HCV entry into Huh-7.5 cells before and after dialysis indicating dialysis is required to determine infectivity in 8 nM bafilomycin-treated samples, but that some loss of overall titer occurs during buffer exchange.
(TIF)

S5 Fig Replication of p7 mutant viruses harboring amino acid changes as position 6, 9, or 12. HCV RNA levels in Huh-7.5 cells determined 8 and 72 hpe showing all monocistronic **(A, B & C)** and bicistronic **(D, E & F)** p7 mutants replicate efficiently. Black diamond (◆) indicates that these control viruses are monocistronic. Note that the WT, GNN, Δp7, and ΔE1E2 titers reported in panels **E** and **F** are duplicated from panel **D** as these viruses were all analyzed in parallel. Data shown correspond to infectious virus titers reported in Fig 7.
(TIF)

S6 Fig p7 structure models of amino acid substitutions at positions 6, 9 and 12. Top view (ER lumen side view) of p7 models 1 and 2 are shown in ribbon representations for all amino acids tested at positions **A)** 6, **B)** 9 and **C)** 12. The side-chain atoms of residues are represented as spheres of the corresponding van der Waals radius. Carbon and hydrogen atoms are in cyan, oxygen atoms in red, nitrogen atoms in blue, and sulfur atoms in yellow.
(TIF)

S1 File PDB coordinates of homology model 1 for p7 HCV J6 strain. (PDB)

S2 File PDB coordinates of homology model 2 for p7 HCV J6 strain. (PDB)
