# Supplementary material for: The N-terminal Helical Region of the Hepatitis C Virus p7 Ion Channel Protein Is Critical for Infectious Virus Production
Source: PLoS Pathog. 2015 Nov 20;11(11):e1005297. doi: 10.1371/journal.ppat.1005297 (PMC4654572; doi:10.1371/journal.ppat.1005297)
Supplement: S1 Fig — For each tryptophan substitution indicated in the title of each page, p7 top and side views of models 1 and 2 are show in both ribbon and surface representations. Top view (ER lumen view) or bottom view (cytosolic side view) highlights the p7 pore while the side view (membrane side view) highlights the p7 surface embedded in the membrane. Model 1 (Ouyang et al. [47]) and Model 2 (Chandler et al. [44]) are colored white and yellow, respectively. The side-chain atoms of tryptophan residues are represented as red spheres of the corresponding van der Waals radius. The effect of Trp substitution on virus production is indicated as a subtitle and evaluation of its possible effect on p7 structure, function, and/or interaction features is commented at the bottom. Comments in red indicate the convergence of possible effects with both models 1 and 2 while comments in black indicate divergences. Additional comments are in blue. (PDF) [file ppat.1005297.s001.pdf]

Mutation A1W *(Bicistronic virus)*

$TCID_{50}$ vs. WT : reduced 9-fold

aa natural variability:
A,T

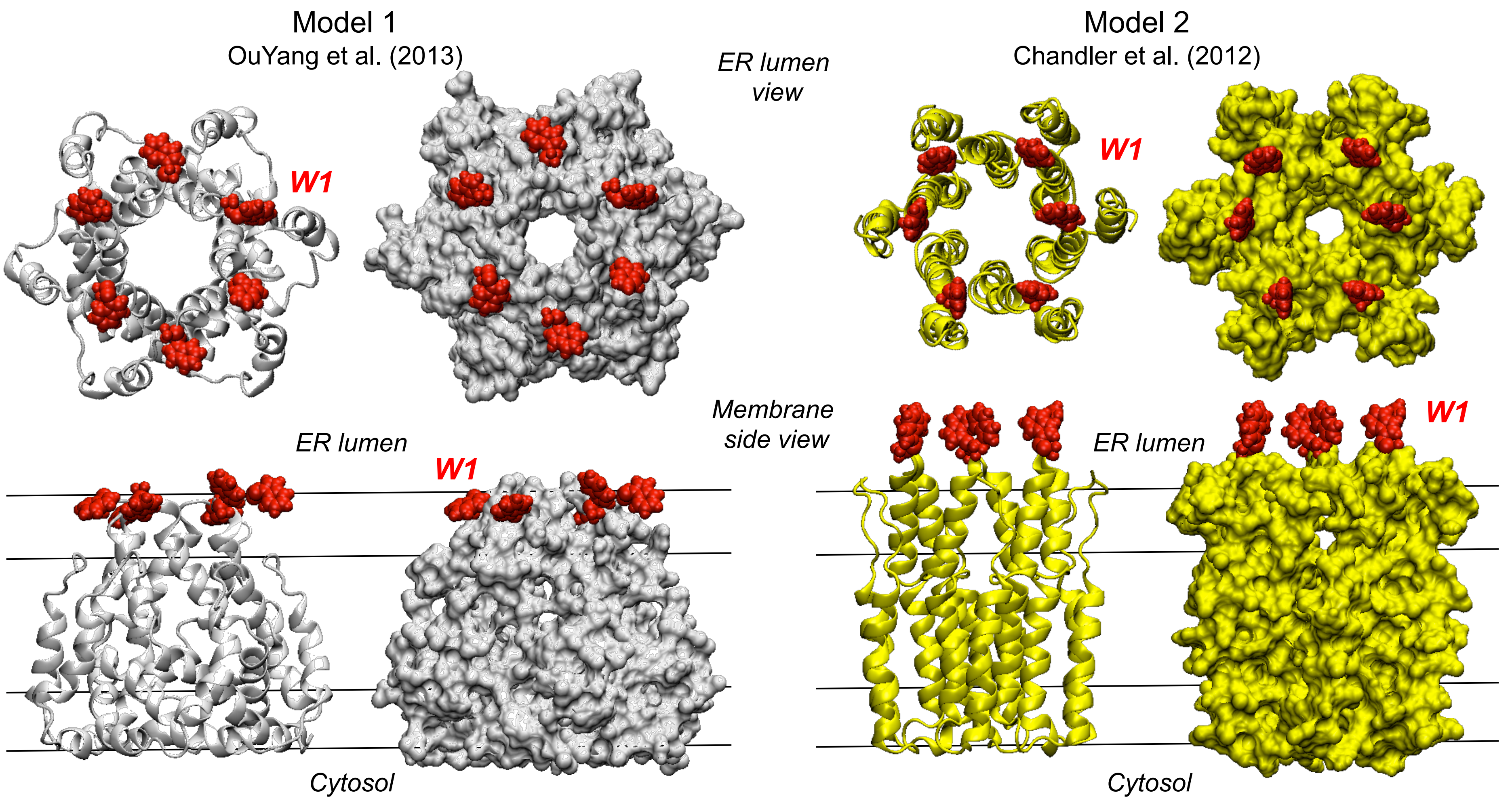

In both models, the A/W1 N-terminal residue is close to the pore entrance and could disturb ion entry and/or flux. W1 is also accessible at the protein surface and might disturb interaction(s) with p7 partner(s).

Mutation L2W *(Bicistronic virus)*

$TCID_{50}$ vs. WT : reduced 2-fold

aa natural variability:
L,M,C,V
hydrophobic position

Model 1
OuYang et al. (2013)

Model 2
Chandler et al. (2012)

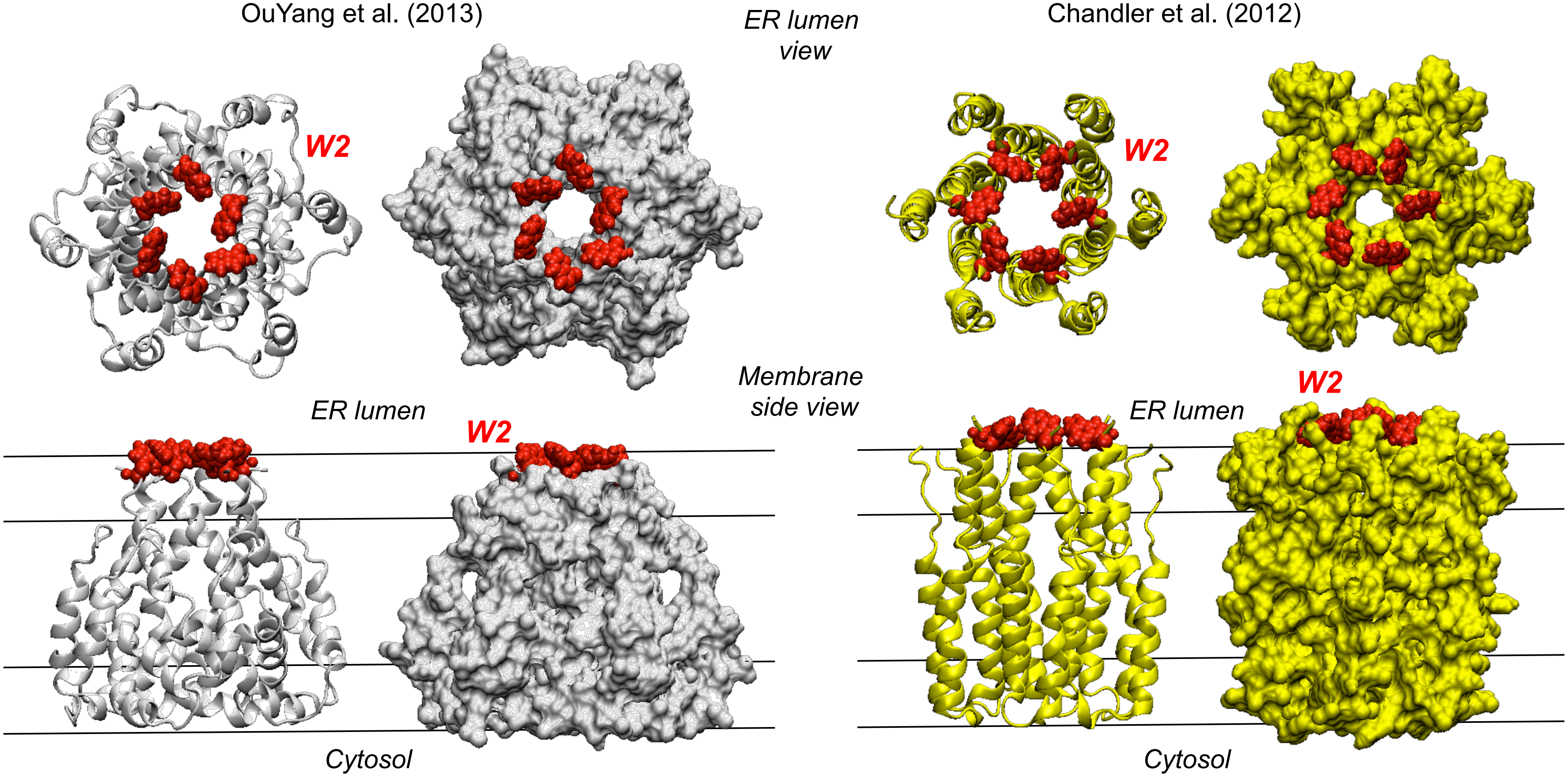

In both models, the L/W2 side chain points to the pore entrance and the bulky Trp side chain could slightly disturb ion entry and/or flux. W2 is also accessible at the protein surface and might disturb interaction(s) with p7 partner(s).

Mutation E3W *(Bicistronic virus)*

$TCID_{50}$ vs. WT : reduced 2-fold

aa natural variability:
E,A,T,S,K
polar position

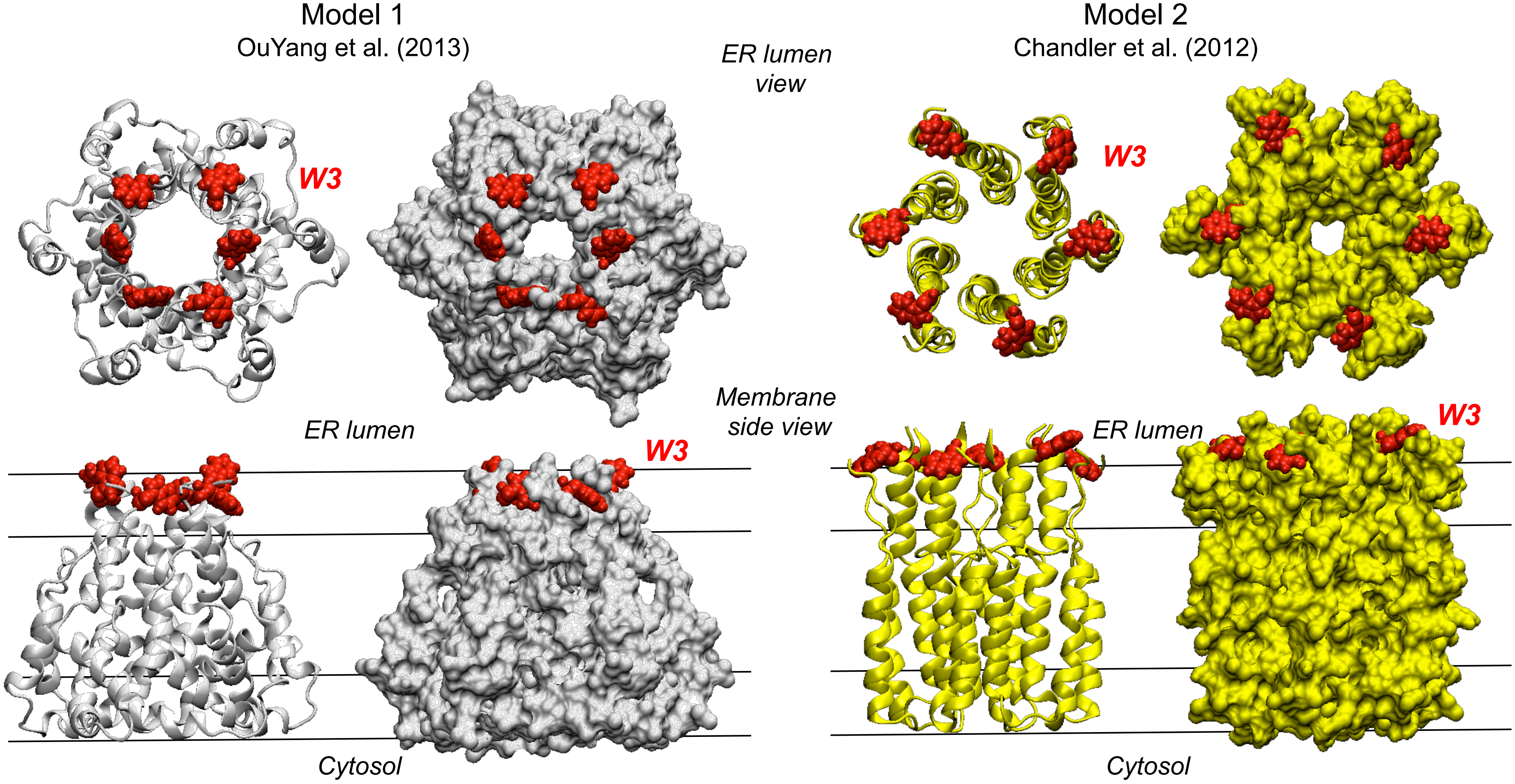

In both models, W3 is accessible at the protein surface and could therefore slightly disturb interaction(s) with p7 partner(s). Alternatively, for model 1, as the W3 side chain is close to the pore entrance and could access the pore lumen, it could slightly disturb the ion flux. For model 2, W3 could slightly disturb the N- and C-terminus interaction network organization of each p7 subunit.

Mutation K4W *(Bicistronic virus)*

$TCID_{50}$ vs. WT : reduced 2-fold

aa natural variability:
N,K,R
polar position

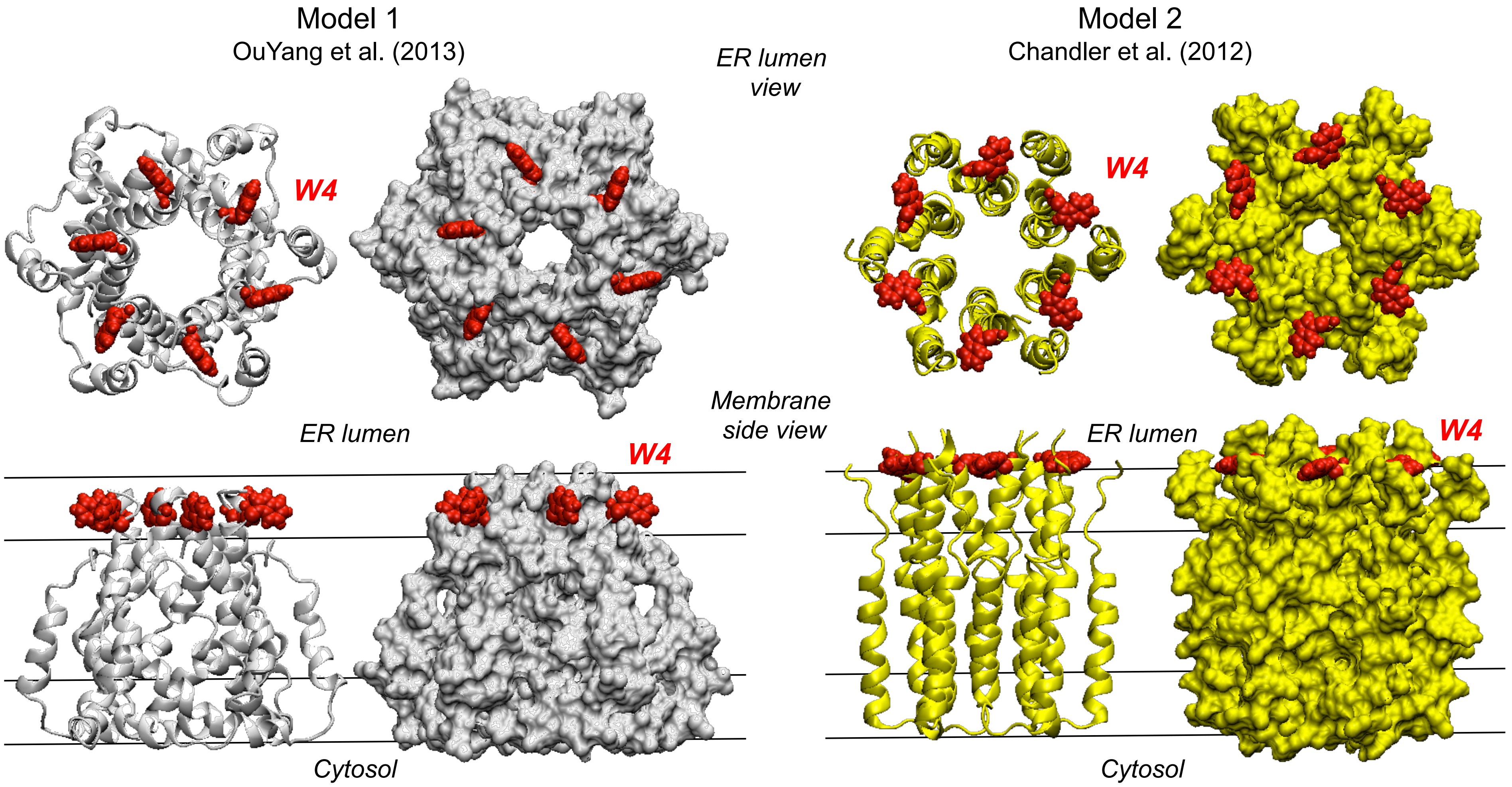

In both models, the K/W4 side chain does not have access to the pore lumen but is accessible at the protein surface on the cytosolic side at the membrane interface.
W4 could thus slightly disturb interaction(s) with p7 partner(s).

Mutation L5W *(Bicistronic virus)*

$TCID_{50}$ vs. WT : enhanced 2-fold

aa natural variability:
L,V,A
hydrophobic position

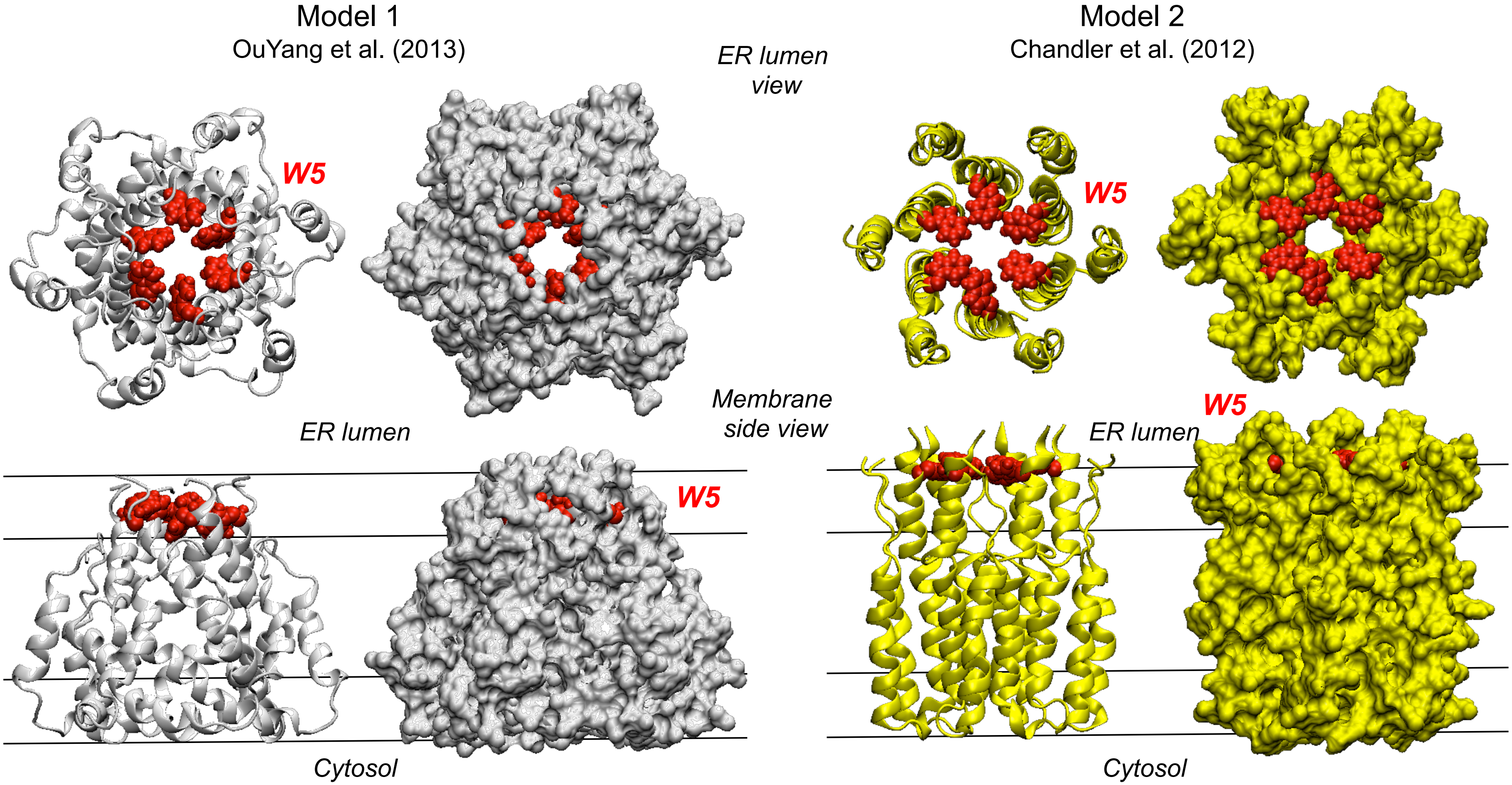

In both models, the L/W5 side chain could point to the pore lumen and thus could play a role in modulating ion flux and/or in ion selectivity.

Mutation V6W *(Bicistronic virus)*

TCID$_{50}$ vs. WT : reduced 75-fold

aa natural variability:
V,I,A
hydrophobic position

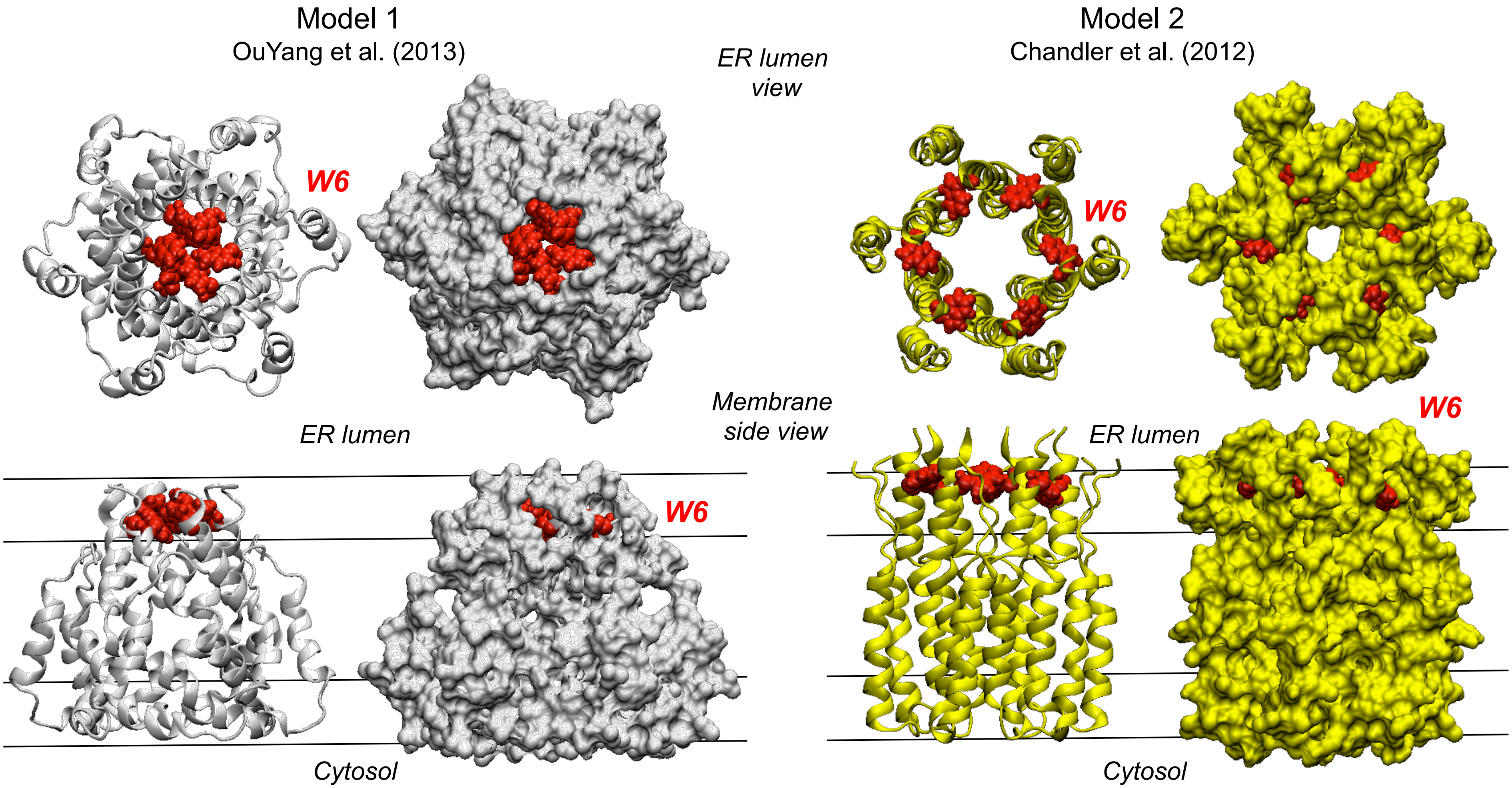

In model 1, the W6 side chain occupies the pore lumen and thus could disturb/prevent ion flux.

In model 2, residue 6 is involved in helix-helix interactions. The W6 side chain could disturb the folding/stability of the N-terminal six-helix bundle and thus disturb ion channel activity.

Mutation I7W *(Bicistronic virus)*

$TCID_{50}$ vs. WT : enhanced 2-fold

aa natural variability:
V,I,L,M,T,N,H
variable position

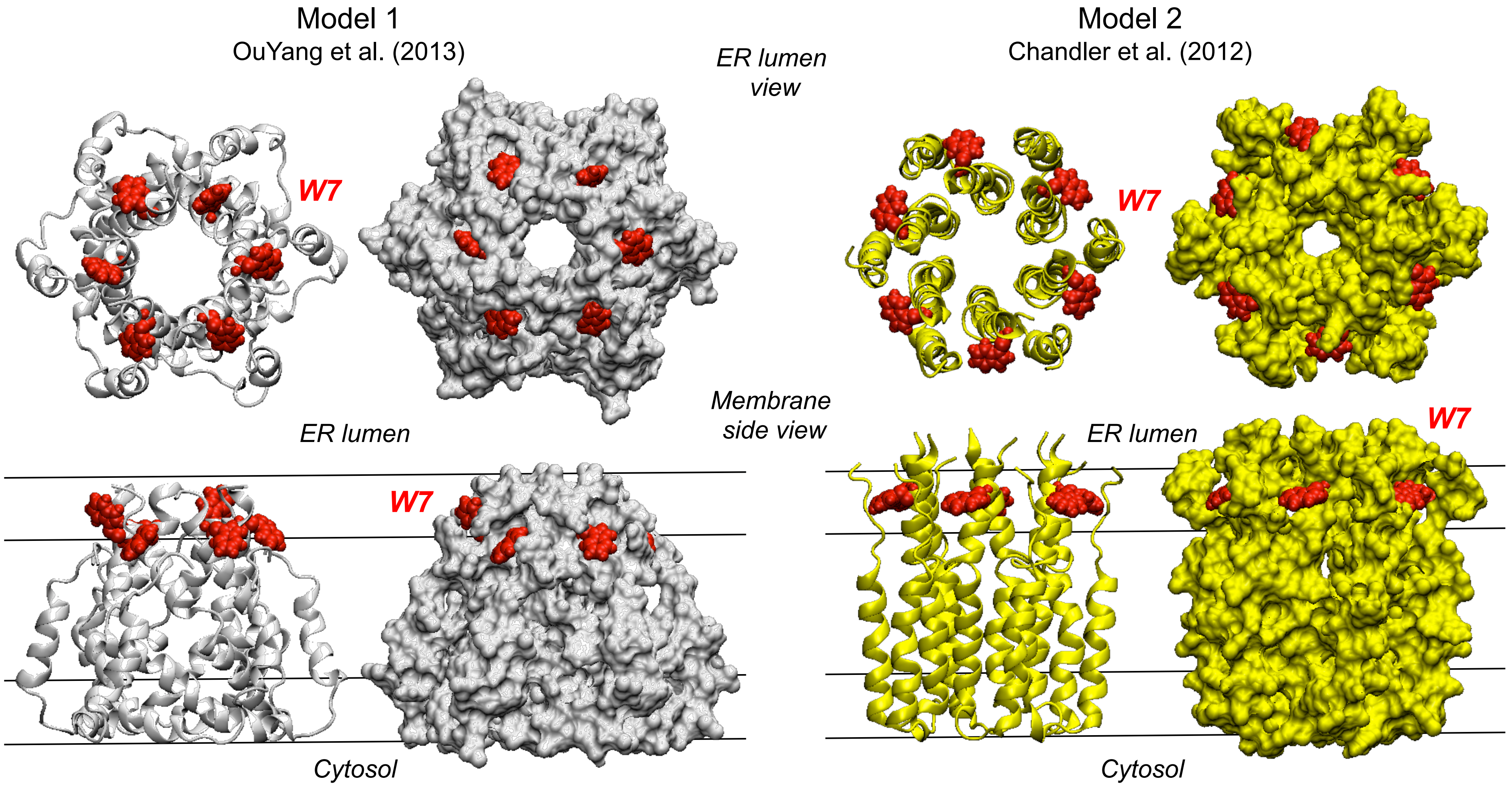

In both models, the I/W7 side chain does not access the pore lumen but is accessible at the protein surface on the cytosolic side at the membrane interface. Both Trp and Ile are hydrophobic residues that could play a similar role in subunit interactions in hexamer assembly and interaction with membrane lipids. The enhanced effect of I7W on virus production could be due to a stabilization of the folding/assembly of the N- and/or C-terminal parts of p7.

Mutation L8W *(Bicistronic virus)*

$TCID_{50}$ vs. WT : enhanced 3-fold

aa natural variability:
L,I
hydrophobic position

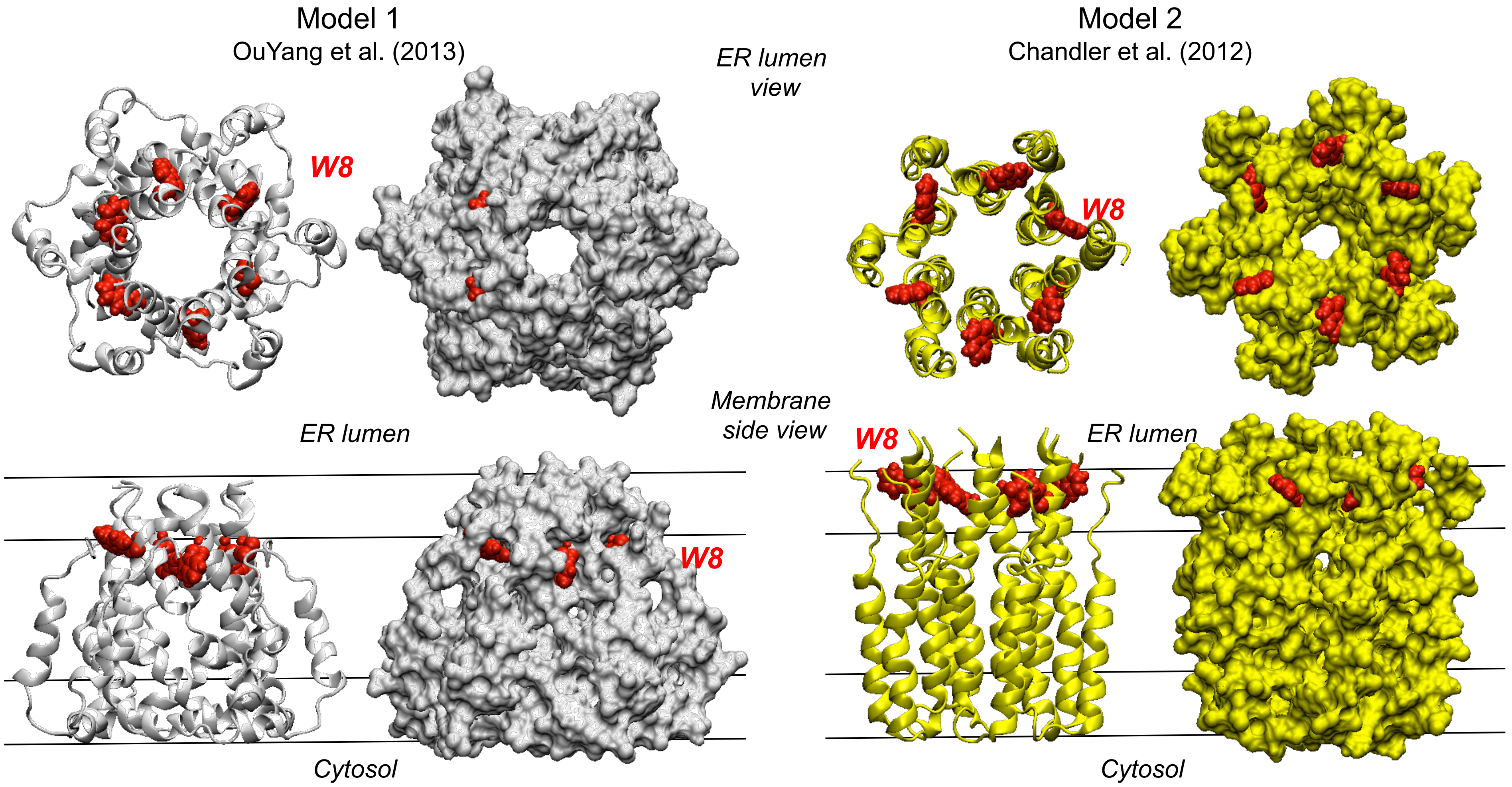

In both models, the L/W7 side chain does not access the pore and is poorly accessible at the protein surface. Both Trp and Leu are hydrophobic residues that could play a similar role in subunit interactions in hexamer assembly. The effect of I7W on virus production could be due to a perturbation of the folding/assembly of the N- and/or C- terminal parts of p7.

Mutation H9W *(Bicistronic virus)*

$TCID_{50}$ vs. WT : no detectable virus production

aa natural variability:
N,H
polar position

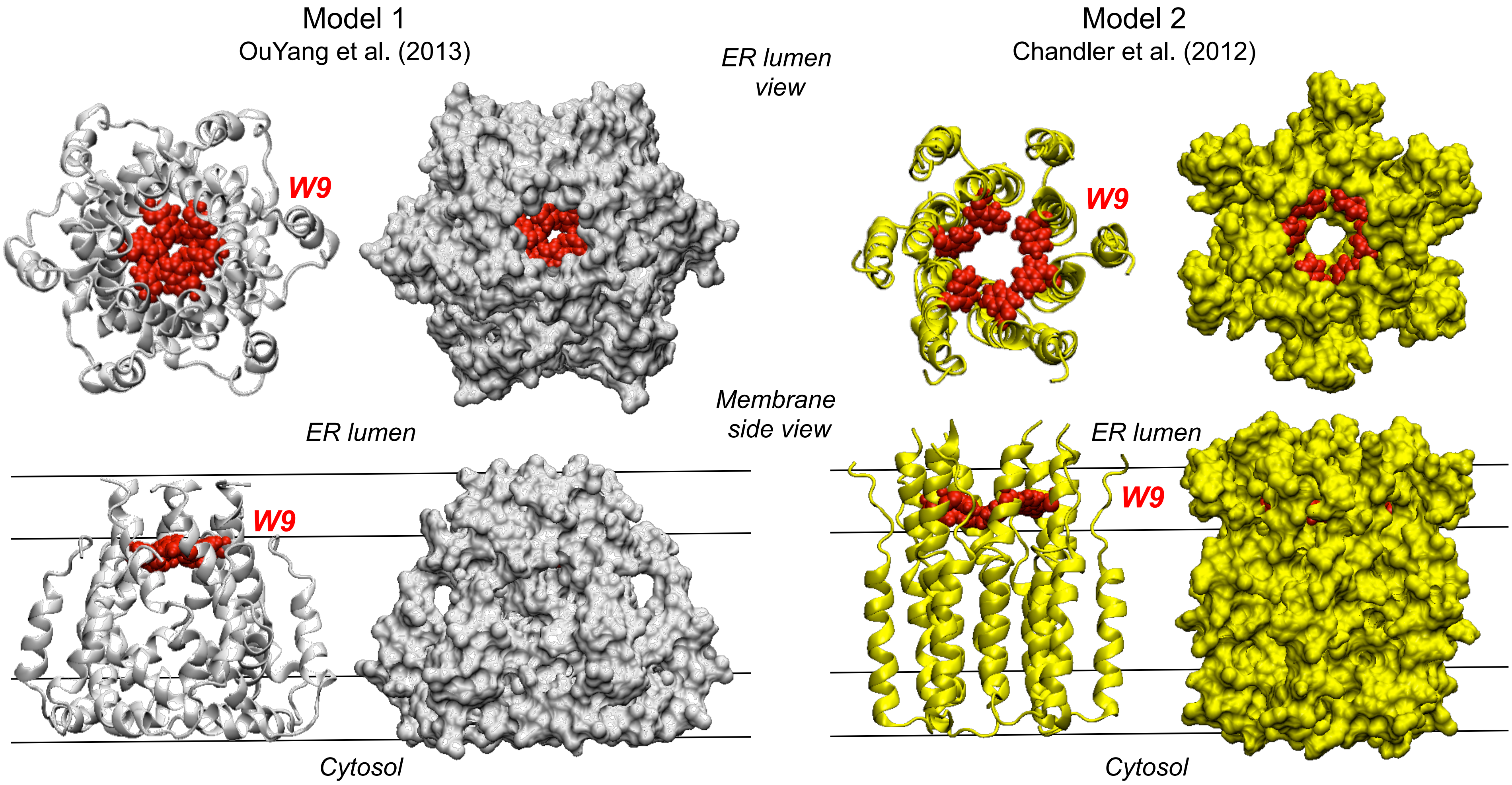

In both models, the H/W9 side chain points to the pore lumen, indicating that H9W could prevent ion flux.

Mutation A10W *(Bicistronic virus)*

TCID$_{50}$ vs. WT : reduced 44-fold

aa natural variability:
A,S
polar position

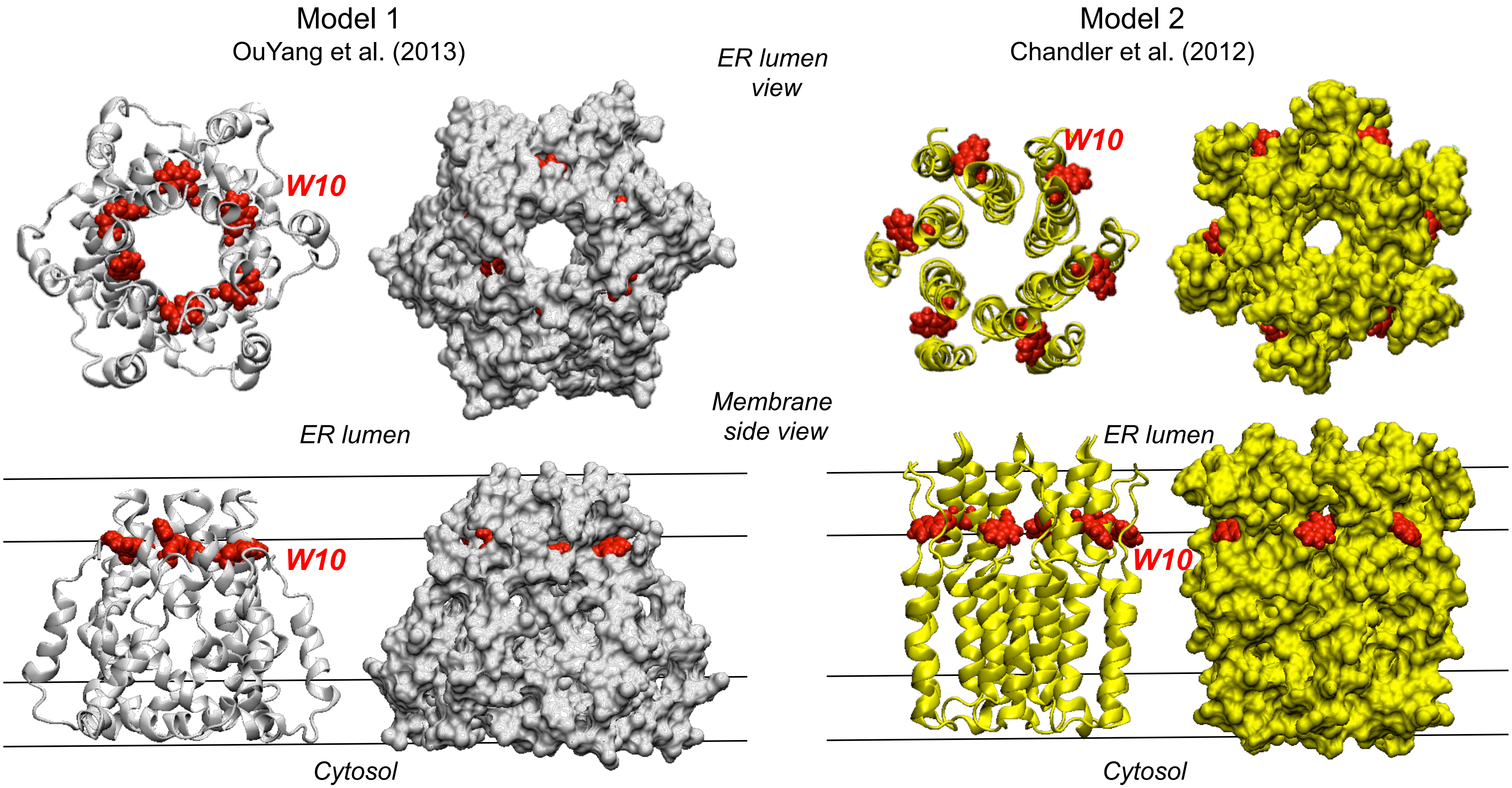

In both models, the W10 side chain does not access the pore lumen. In model 2, the W10 side chain is accessible at the protein surface and thus could disturb interaction(s) with p7 partner(s). This hypothesis might be also valid for model 1, although the accessibility of W10 is limited at the surface of this model. Because the W10 side chain interacts with many residues of neighboring subunits in model 1, an alternative explanation would be that this mutation rigidifies the oligomeric structure and thus prevents conformational changes that are required for pore functioning.

Mutation A11W *(Bicistronic virus)*

$TCID_{50}$ vs. WT : enhanced 3-fold

aa natural variability:
A,V,L,I,T,S,M
variable position

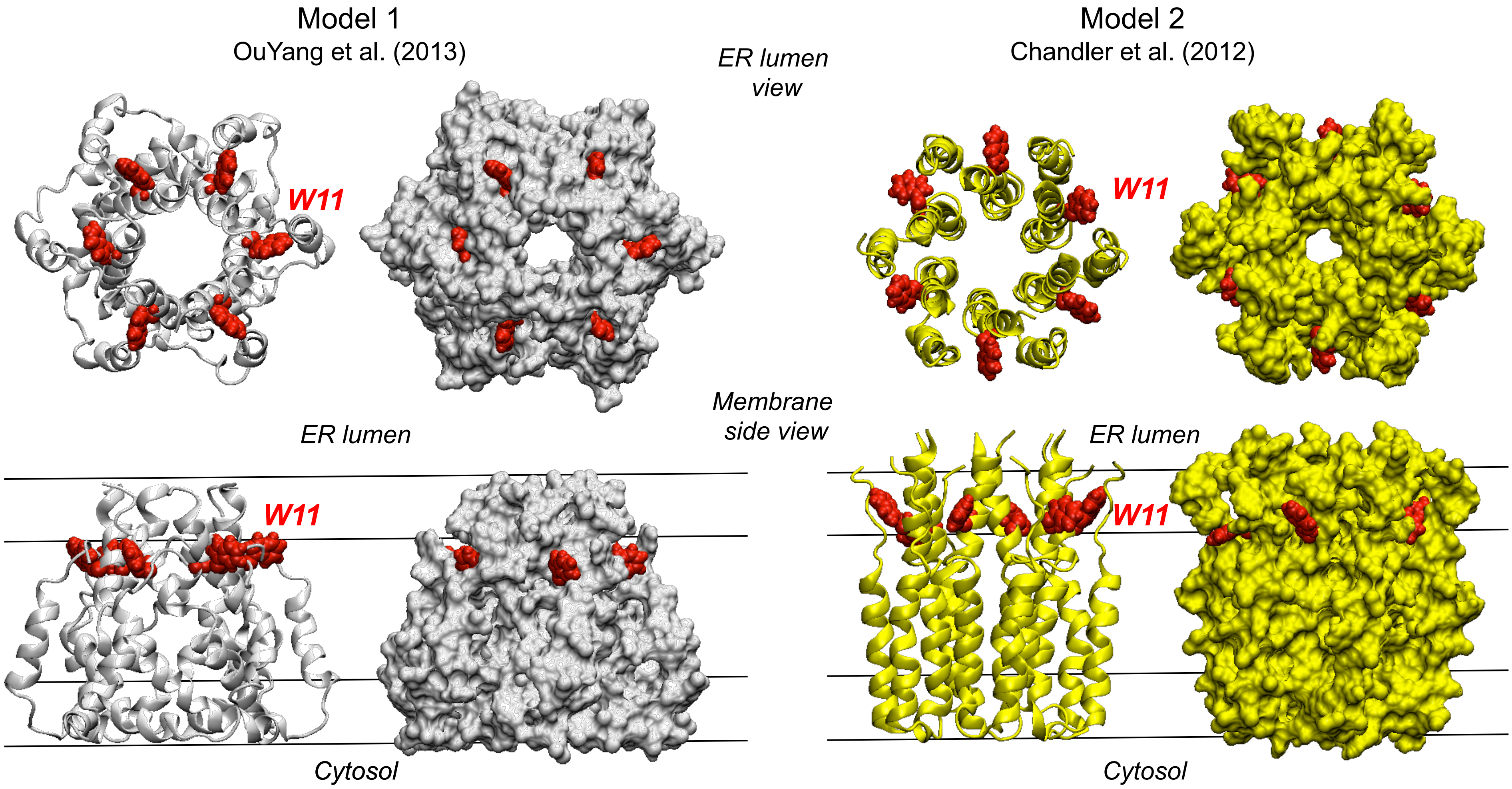

In both models, the A/W11 side chain is accessible at the protein surface and is close to residues of the C-terminus of p7. The A11W mutation could stabilize interaction(s) with p7 partner(s), either directly and/or by stabilizing the folding/assembly of the C-terminal region of p7.

Mutation S12W *(Bicistronic virus)*

TCID$_{50}$ vs. WT : reduced 8-fold

aa natural variability:
S,A
polar position

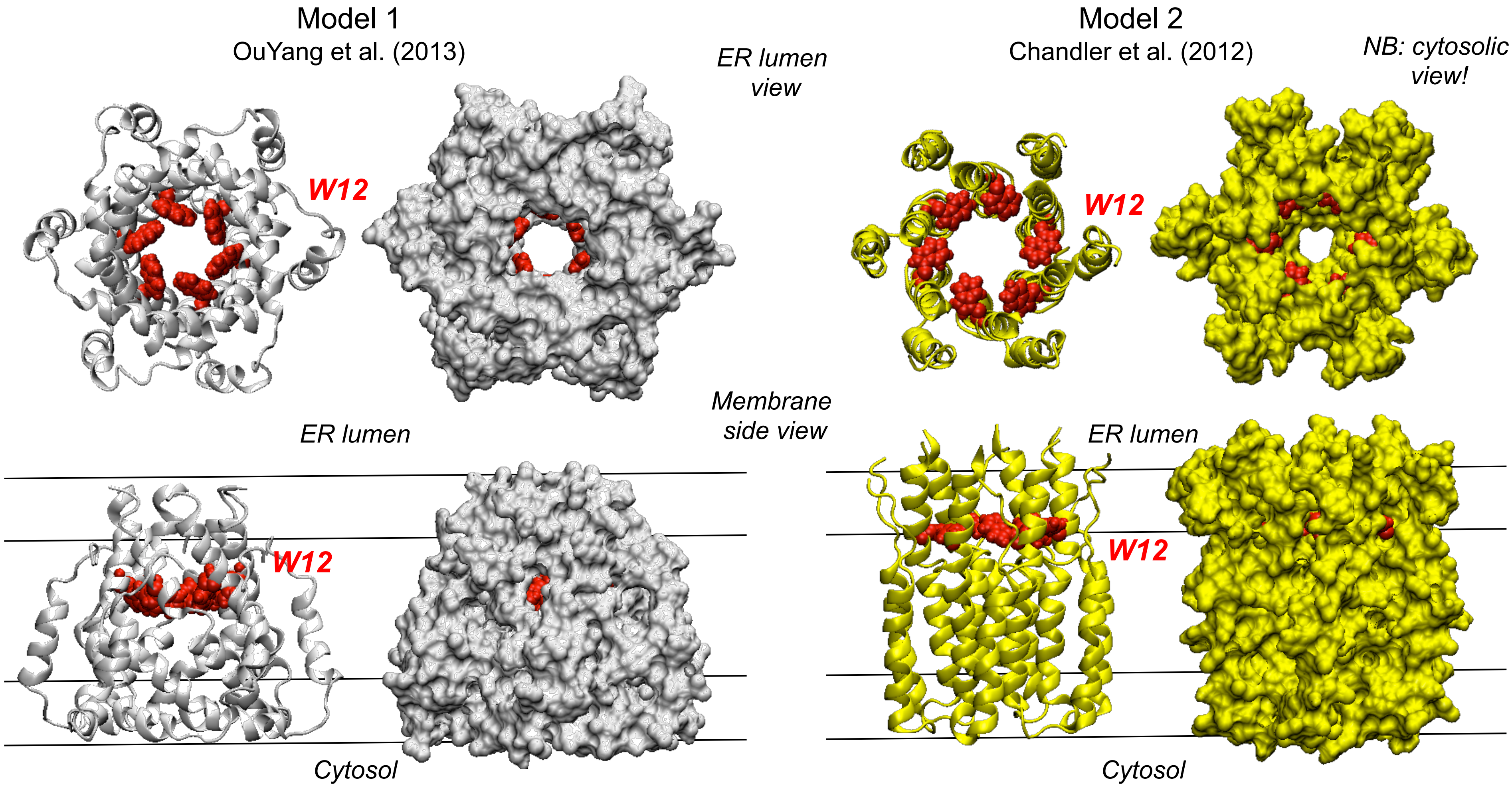

In both models, S/W12 points to the pore lumen, indicating that mutation S12W can disturb ion flux.

Mutation A13W *(Bicistronic virus)*

$TCID_{50}$ vs. WT : equivalent

aa natural variability:
A,V,L,M,T
hydrophobic position

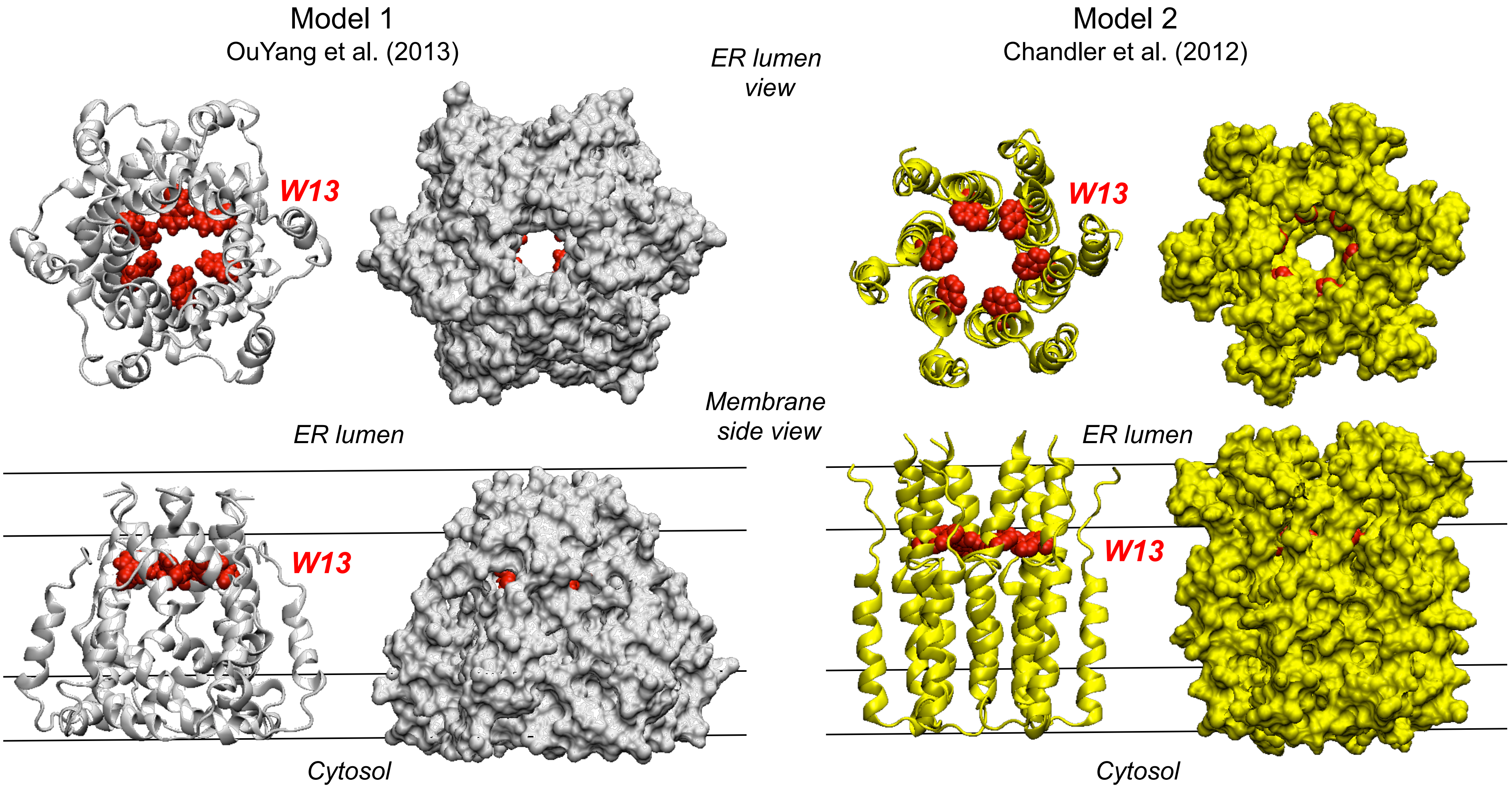

In both models, the side chain of amino acid 13 partially points to the pore and could be involved in p7 subunit interactions. The absence of an effect of this mutation in modulating ion flux is questionable.

Mutation A14W *(Monocistronic virus)*

TCID$_{50}$ vs. WT : equivalent

aa natural variability:
A,V,S
variable position

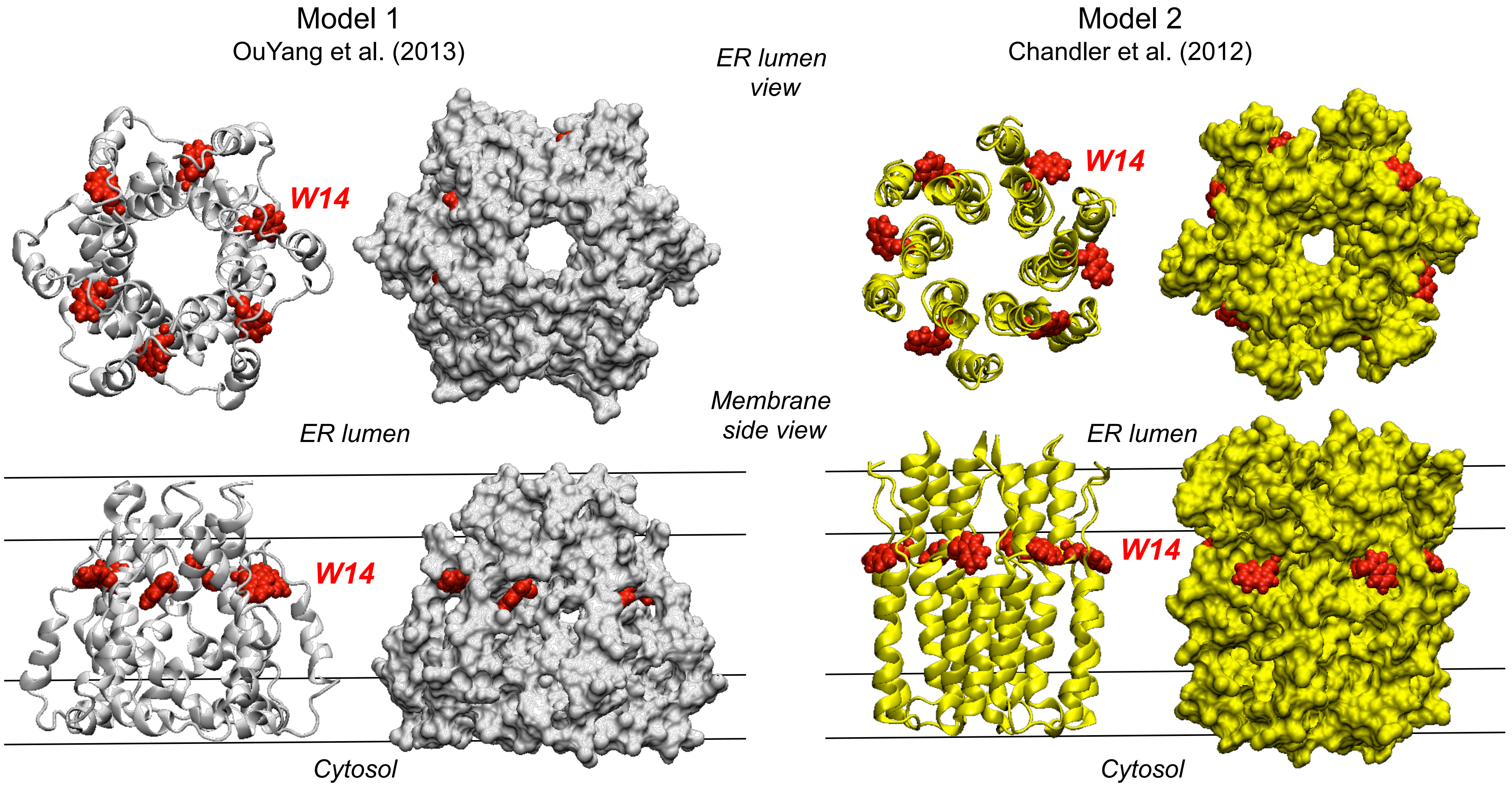

In both models, the A/W14 side chain does not access the pore but is accessible at the protein surface at the level of the membrane hydrophobic core. Despite its bulky side chain, W14 has no major impact on p7 structure and assembly, likely explaining the absence of any detrimental effect on virus production.

Mutation S15W *(Monocistronic virus)*

$TCID_{50}$ vs. WT : reduced 2-fold

aa natural variability:
G,S,A
polar position

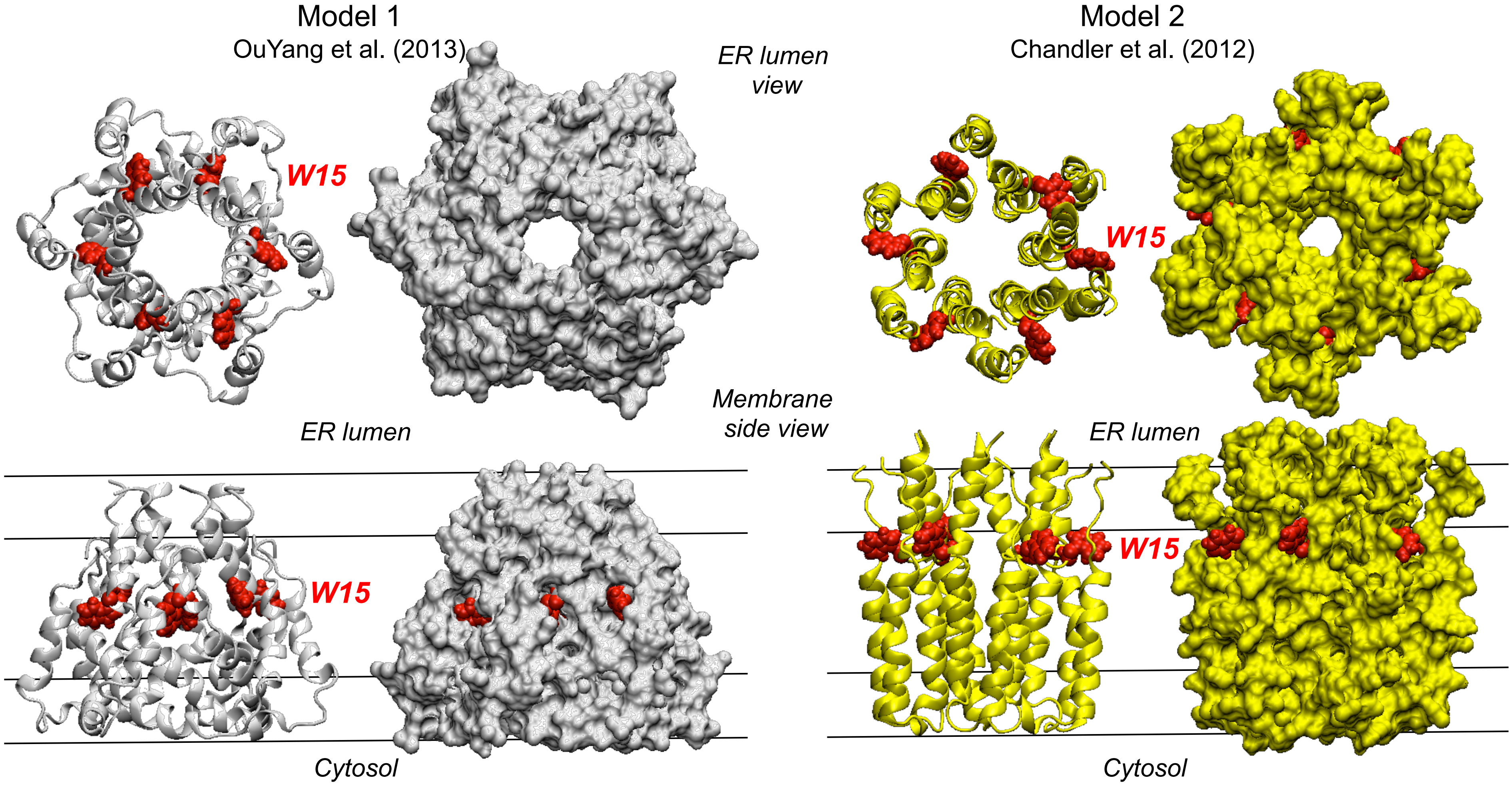

In both models, the side chain of amino acid 15 does not access the pore lumen but is accessible at the protein surface and thus might disturb some interaction(s) with p7 partner(s). However, because the W15 side chain interacts with C-terminal residues of neighboring subunits in both models, an alternative explanation would be that this mutation disturbs the folding and/or rigidifies the oligomeric structure and thus prevents conformational changes required for pore functioning.

Mutation C16W *(Monocistronic virus)*

$TCID_{50}$ vs. WT : enhanced 5-fold

aa natural variability:
T,A,S,N,C
polar position

Model 1
OuYang et al. (2013)

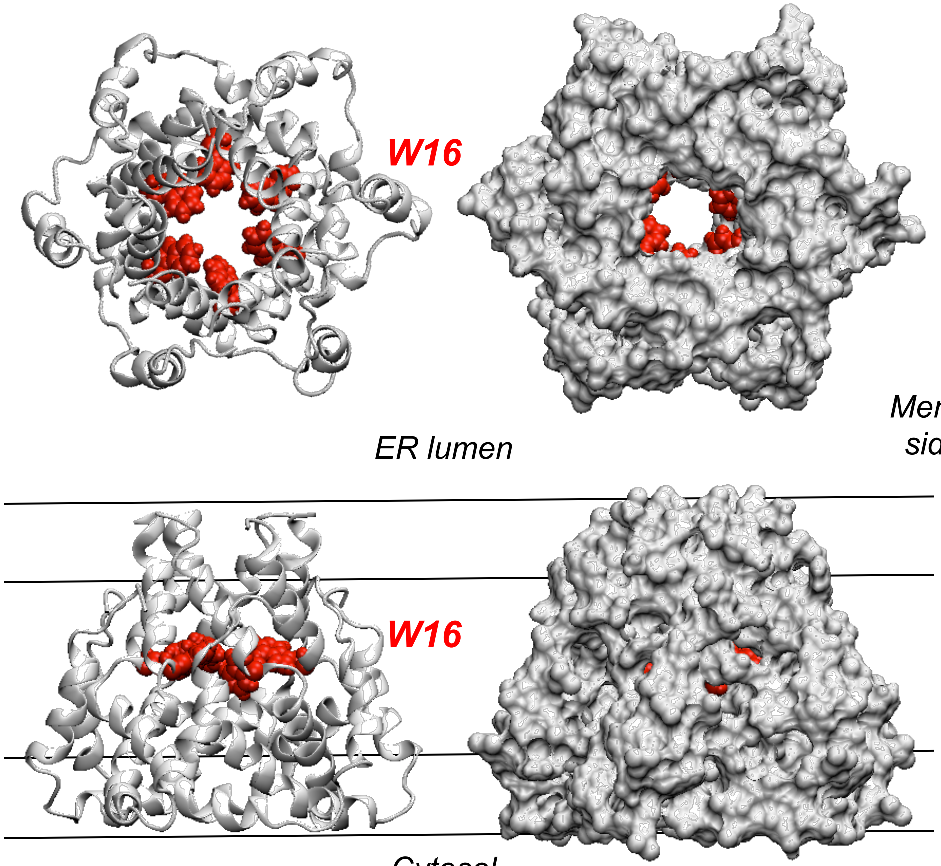

Model 2
Chandler et al. (2012)

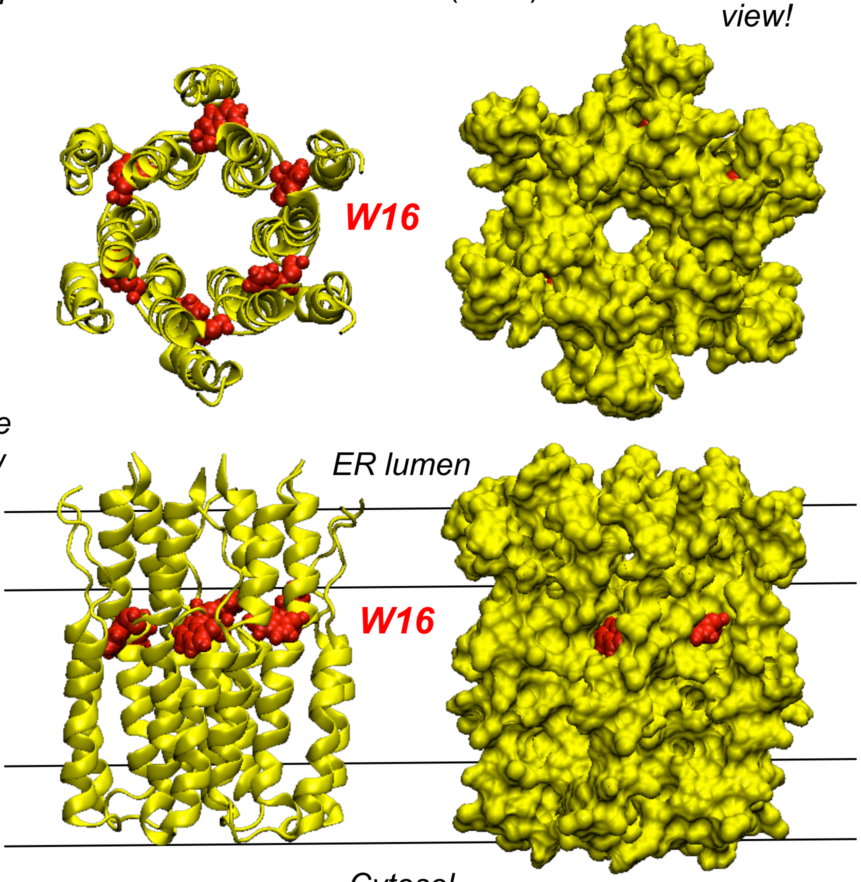

In model 1, the C/W16 side chain is in the pore lumen. It is thus surprising that mutation C16W would have no effect on the ion flux in this model.

In model 2, the C/W16 side chain does not access the pore lumen and its accessibility to the protein surface is limited. This position at the subunit interface could be involved in the stability of p7 assembly.

Mutation N17W *(Monocistronic virus)*

TCID$_{50}$ vs. WT : enhanced 2-fold

aa natural variability:
H,N,Q,A,R
polar position

Model 1
OuYang et al. (2013)

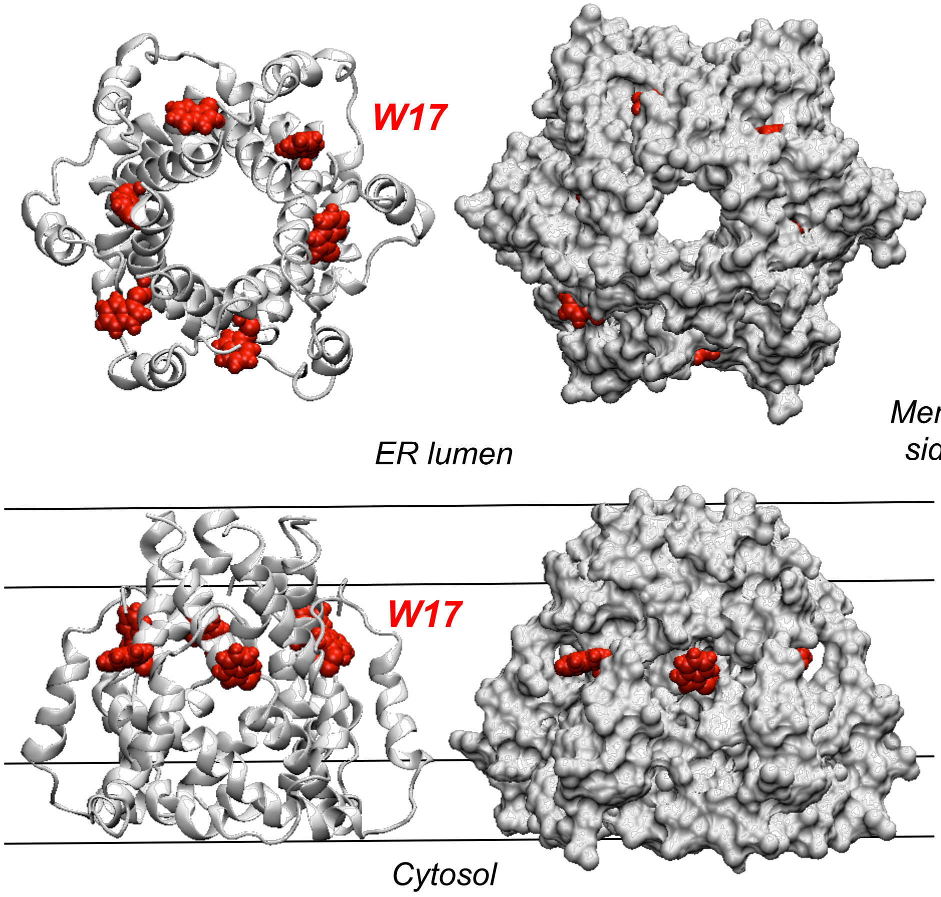

ER lumen view

Membrane side view

Model 2
Chandler et al. (2012)

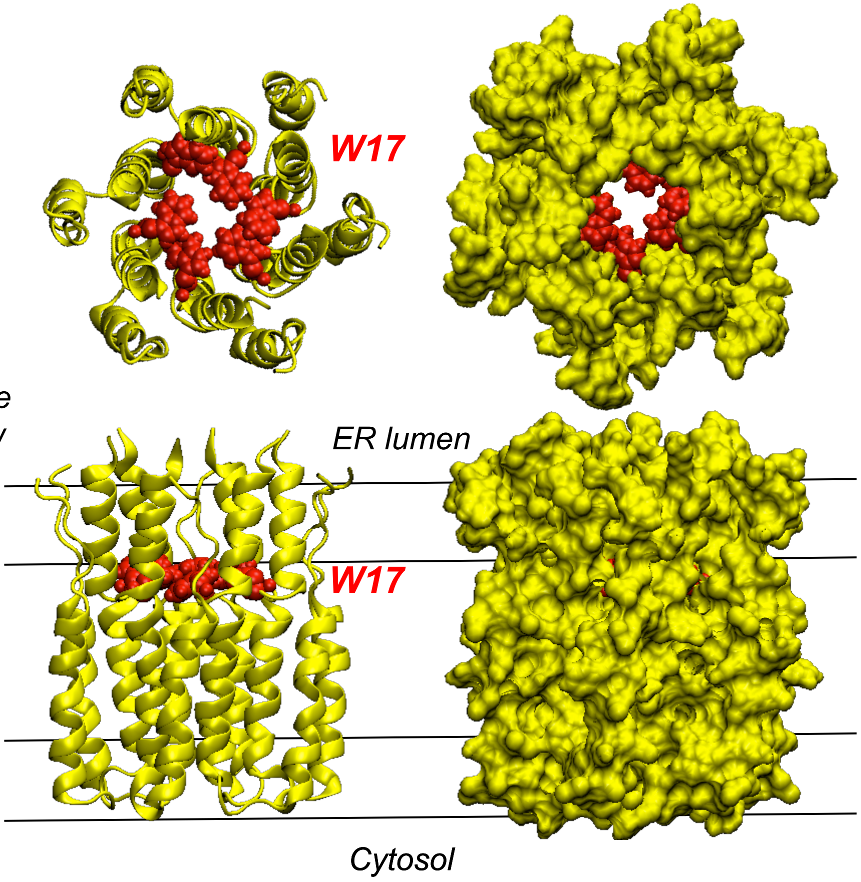

In model 1, the N/W17 side chain does not access the pore lumen and is accessible at the protein surface at the level of the membrane hydrophobic core. The N17W mutation might thus alter some interactions with p7 partner(s).

In model 2, the N/W17 side chain is in the pore lumen, indicating that the N17W mutation could seriously disturb the ion flux.

Mutation G18W *(Monocistronic virus)*

TCID$_{50}$ vs. WT : enhanced 6-fold

aa natural variability:
G only
flexible position

Model 1
OuYang et al. (2013)

Model 2
Chandler et al. (2012)

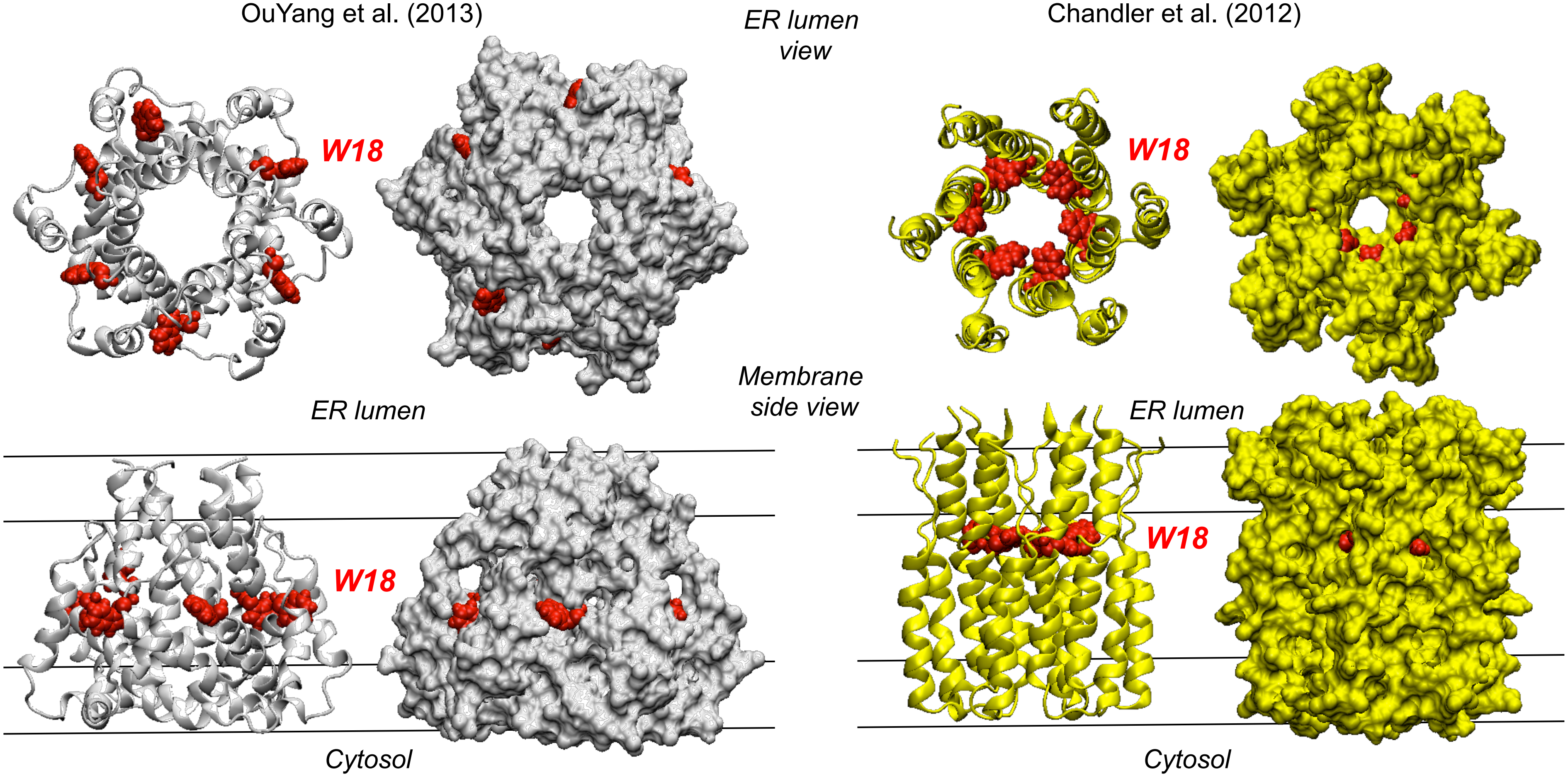

In model 1, the G/W18 side chain does not access the pore lumen and is accessible at the protein surface at the level of the membrane hydrophobic core. Mutation G18W might indirectly modulate p7 activity by acting on the stability of p7 assembly.

In model 2, the G/W18 side chain points to the pore lumen, indicating that the G18W mutation could modulate ion flux.

Mutation F19W *(Monocistronic virus)*

$TCID_{50}$ vs. WT : equivalent

aa natural variability:
L,I,F,W,V,P,T,M
hydrophobic, variable position

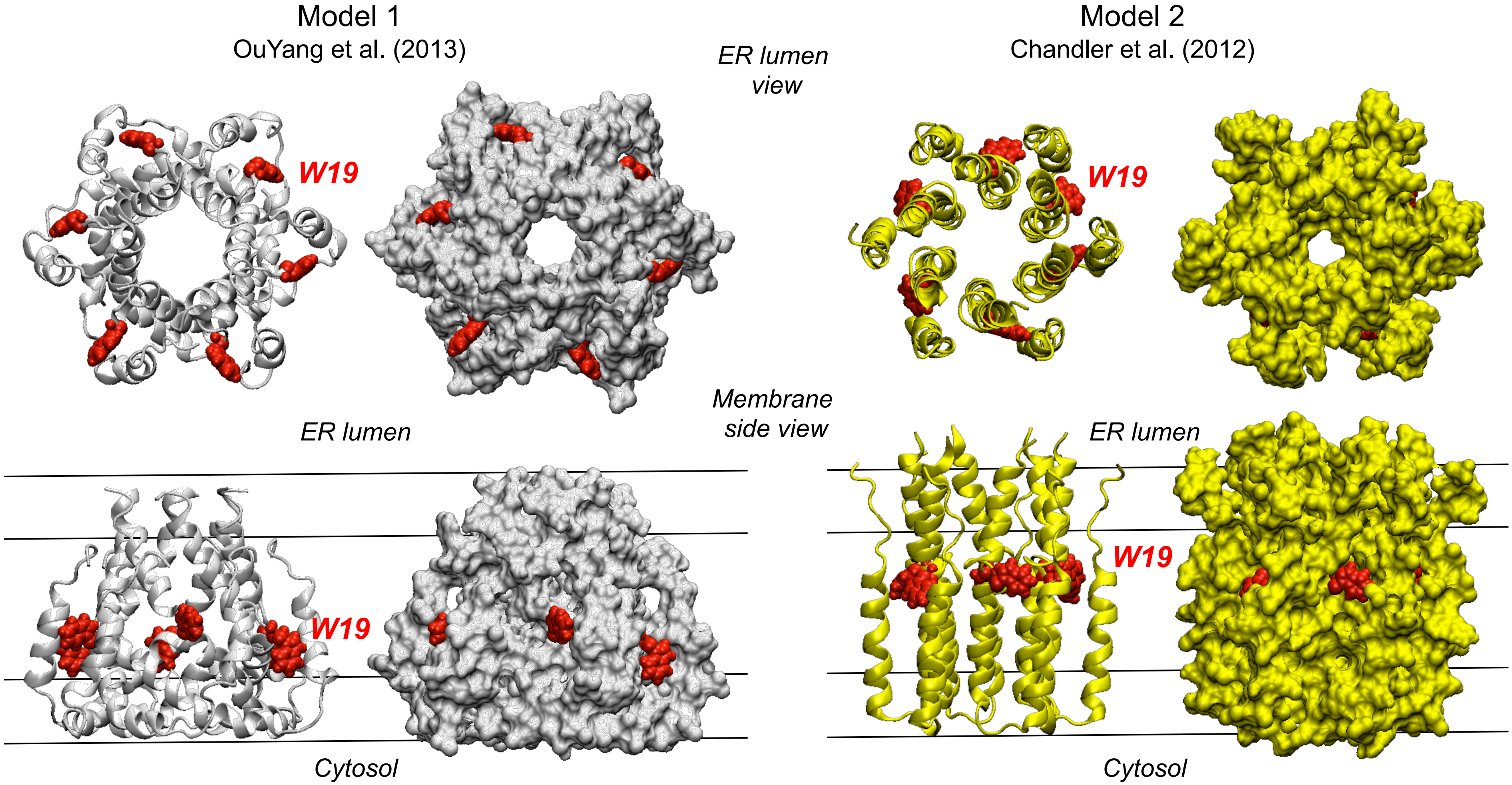

This hydrophobic position is quite variable and W is observed at this position in p7 in most subtypes of genotype 6. This residue is located at the level of the membrane hydrophobic layer.

In model 1, amino acid 19 is freely accessible at the p7 surface and thus might modulate interaction(s) with lipids and/or p7 partners. In model 2, amino acid 19 could play a role in the intra- and inter-subunit interactions. It is also accessible at the protein surface and thus might modulate interaction(s) with p7 partner(s) and/or lipids.

Mutation L20W *(Monocistronic virus)*

$TCID_{50}$ vs. WT : equivalent

aa natural variability:
L,V,G,A,I,W,F,M,Y,M,C,E
variable position

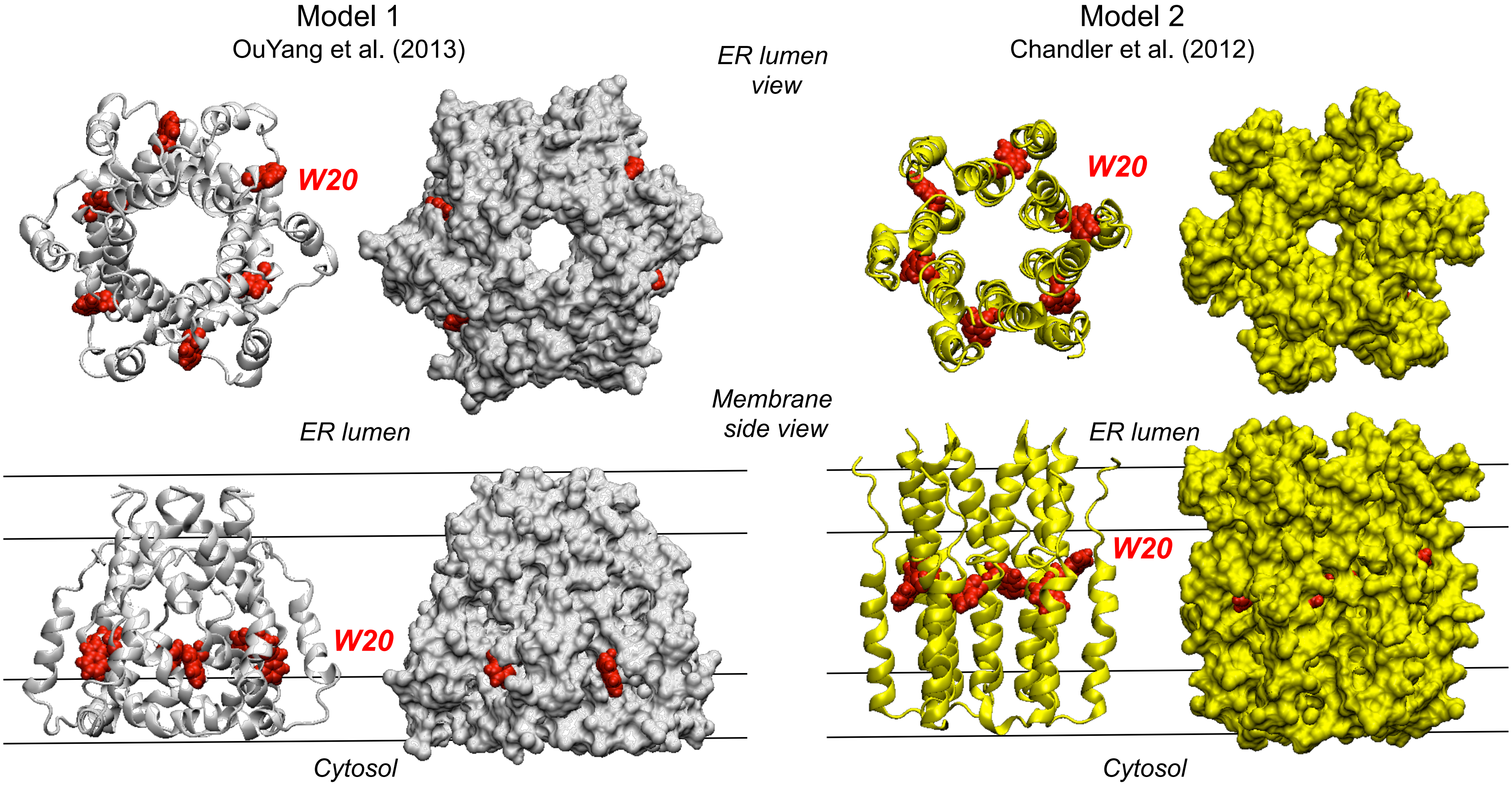

W is often observed at this position in natural variants, especially in genotypes 3a, 4a, 6a and 7a, indicating that it is well tolerated/required at this position. Moreover, this position is tolerant of mutation to hydrophobic residues *(except Glu in genotypes 6g)*. In both models, this residue is involved in p7 oligomer assembly. In addition, in model 1, it is accessible at the protein surface at the level of hydrophobic core of the membrane where is could interact with lipids.

Mutation Y21W *(Monocistronic virus)*

$TCID_{50}$ vs. WT : equivalent

aa natural variability:
W,Y,S,C,P
variable position

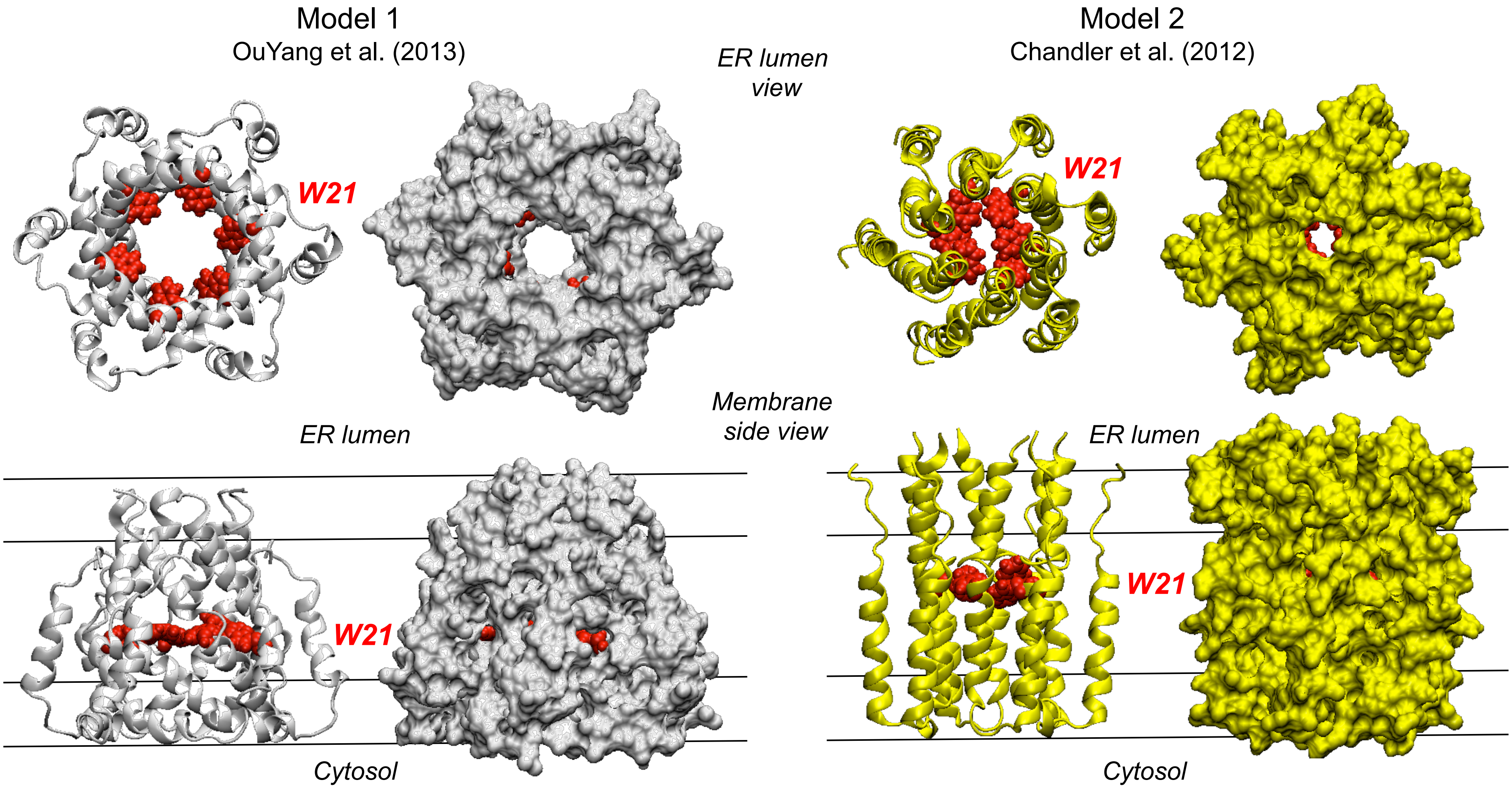

W is often observed at this position in natural variants, especially in genotypes 2b, 3 5 and 6, indicating that it is well tolerated/required at this position. In both models, Y21 as well as W21 point to the pore lumen. The absence of effect of the Y21W mutation on virus production might be explained by the similar physico-chemical properties of Trp and Tyr side chains: aromatic in nature with the presence of a polar group. Both residues likely play a similar role in ion channeling through p7.

Mutation F22W *(Monocistronic virus)*

$TCID_{50}$ vs. WT : enhanced 2-fold

aa natural variability:
F,Y,G,A,C,L,S,T
variable position

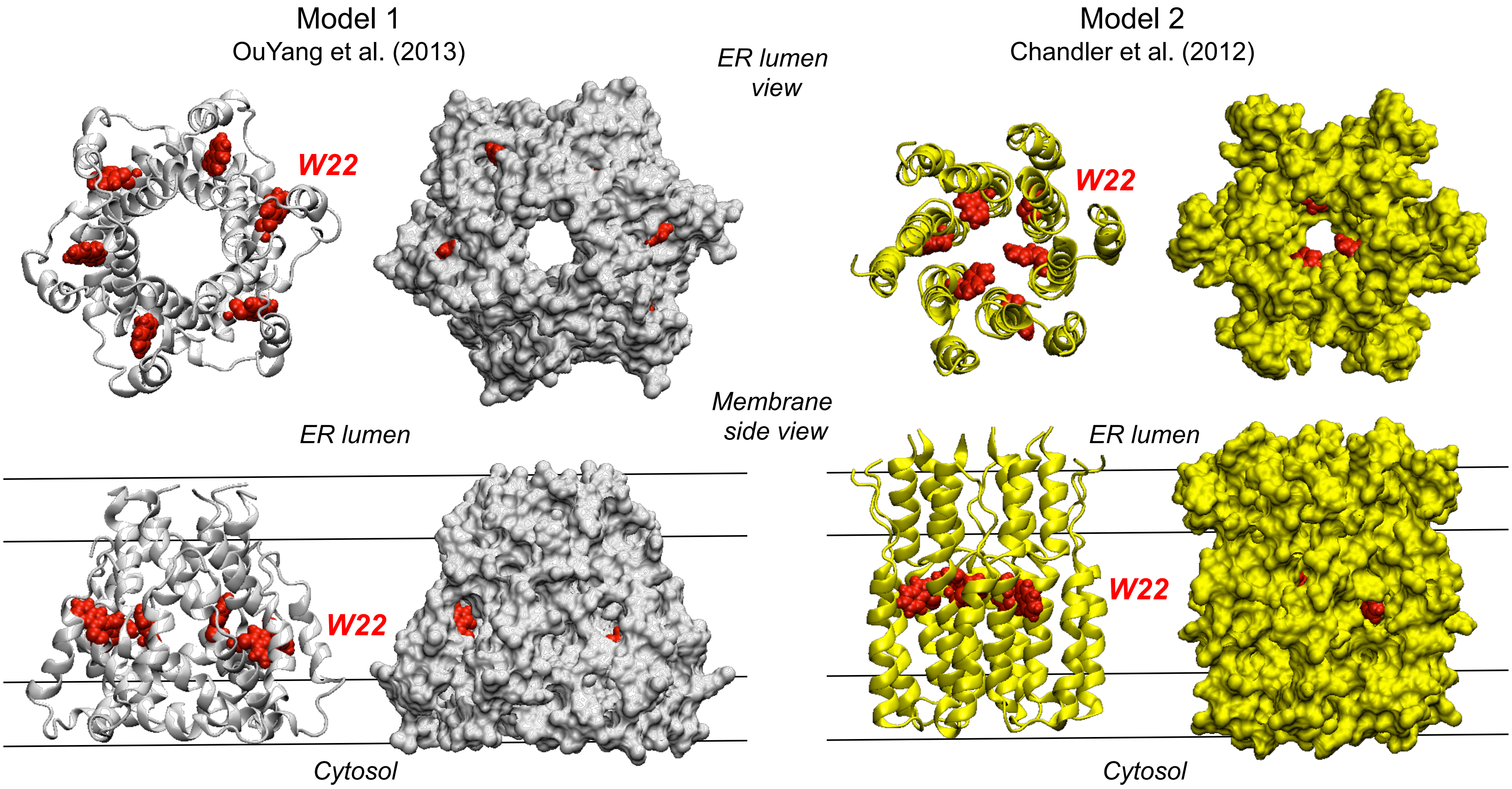

In model 1, the F/W22 side chain is located at the subunit interface. The increased virus production observed with F22W mutation could be linked to a stabilization of p7 oligomer assembly.

In model 2, the F/W22 side chain can access both the pore lumen and subunit interface. Mutation F22W could stabilize the p7 oligomer assembly and/or facilitate ion flux.

Mutation V23W *(Monocistronic virus)*

$TCID_{50}$ vs. WT : reduced 2-fold

aa natural variability:
L,V,I,F
hydrophobic position

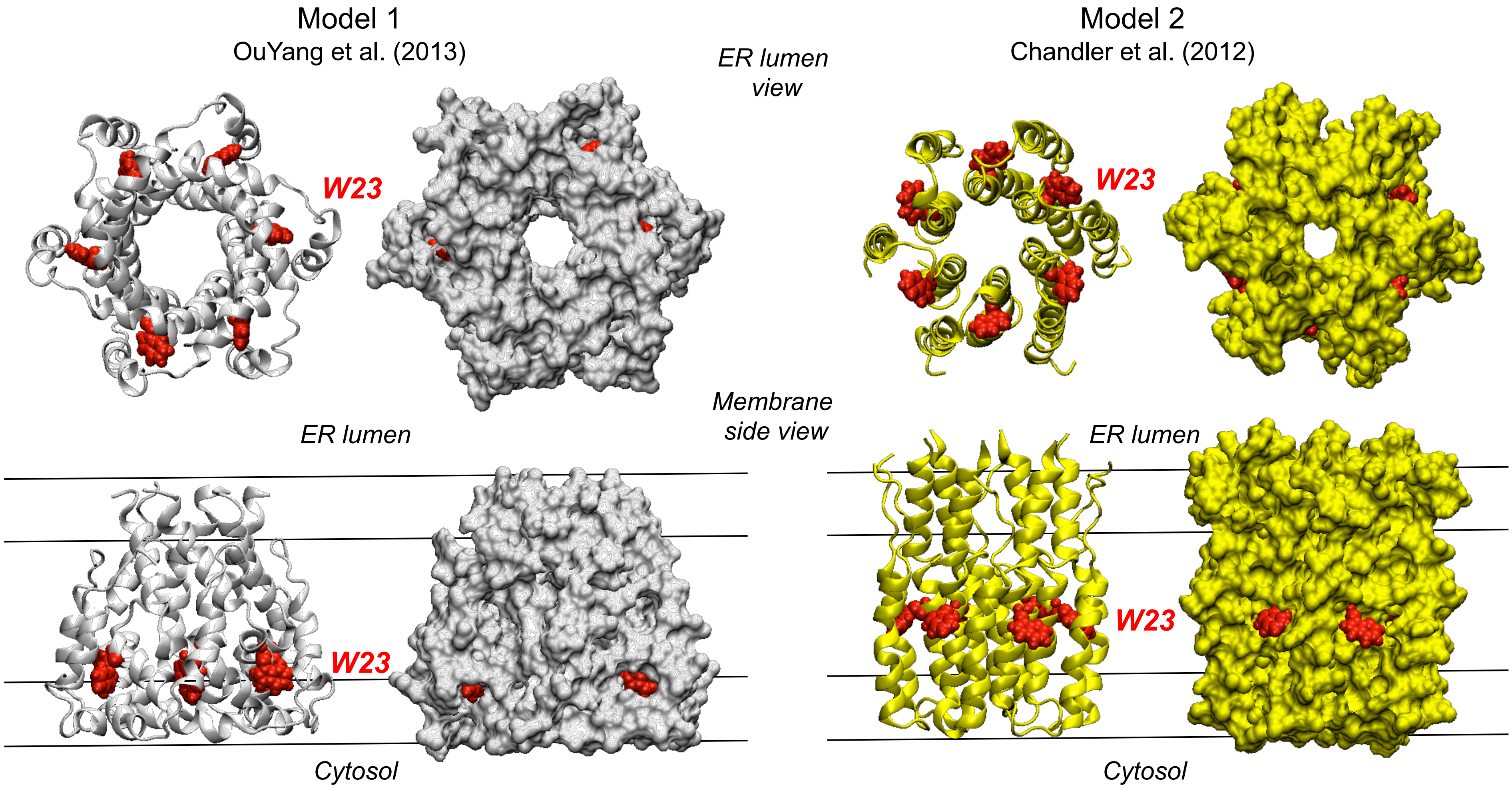

In model 1, the V/W23 side chain is located at the subunit interface and likely involved in p7 oligomer assembly. Its access to the protein surface is very limited.

In model 2, the V/W 23 side chain is accessible at the protein surface at the level of the hydrophobic core of the membrane (and could thus be involved in interactions with p7 partners).

Mutation I24W *(Monocistronic virus)*

$TCID_{50}$ vs. WT : reduced 6-fold

aa natural variability:
V,I,L,A,M
hydrophobic position

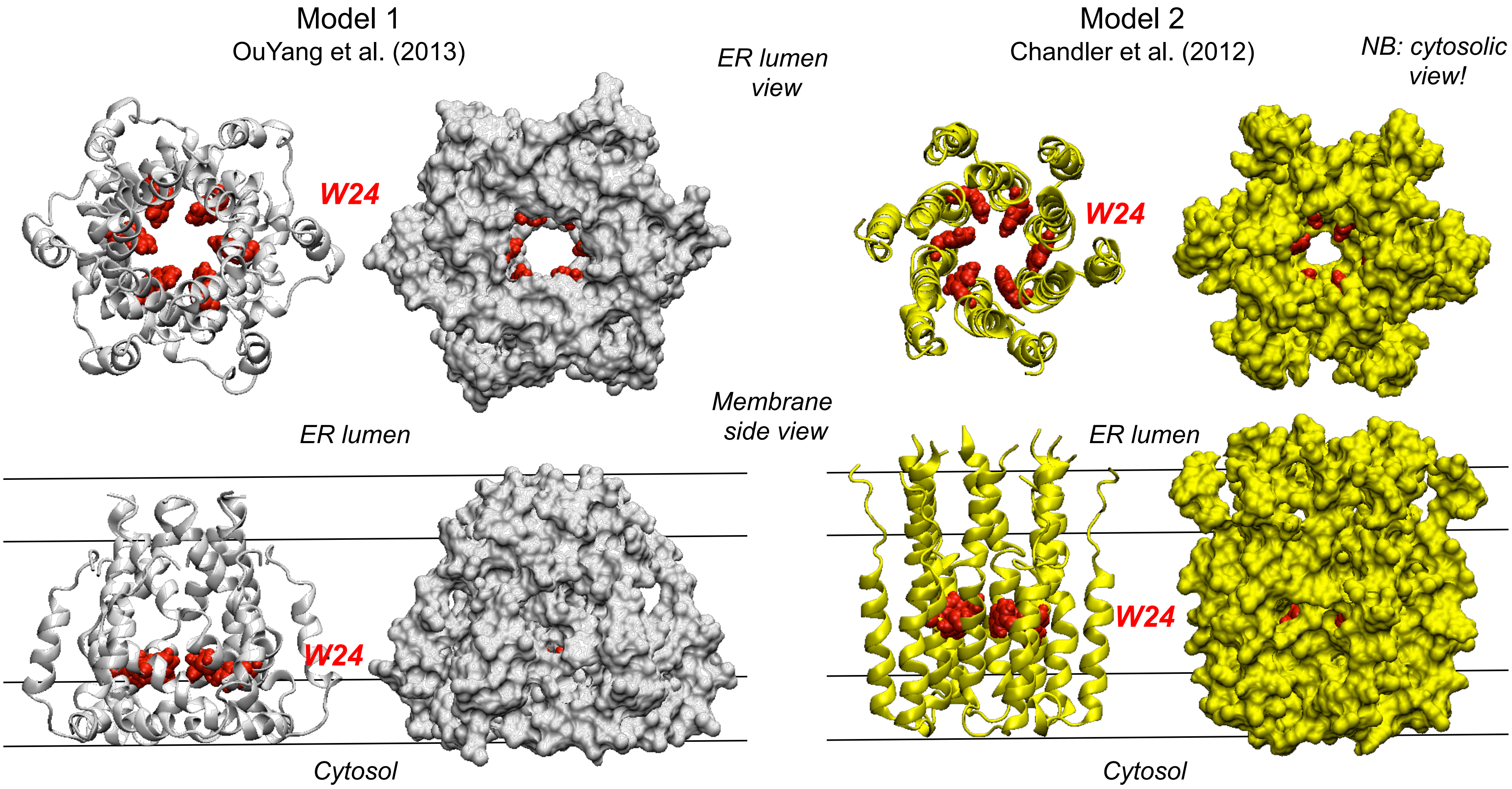

In both models, the I/W24 side chain points to the pore lumen, indicating that mutation I24W can modulate ion flux.

Mutation F25W *(Monocistronic virus)*

TCID$_{50}$ vs. WT : enhanced 12-fold

aa natural variability:
F,A,V
hydrophobic position

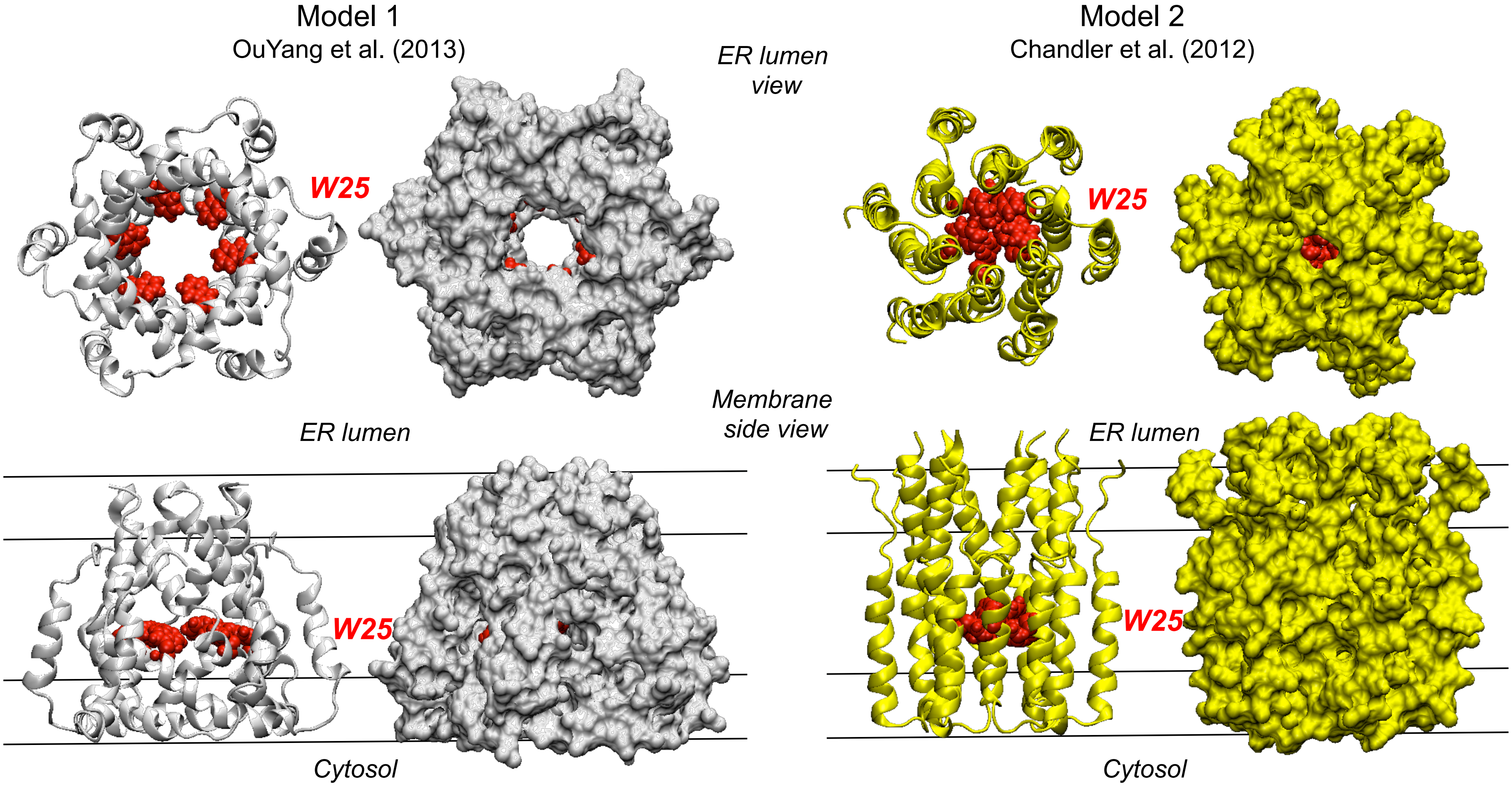

In both models, F25 as well as W25 side chains point to the pore lumen. In model 2, Phe forms a very efficient hydrophobic barrier to the passage of ions. In the F25W mutant, the higher polarity of the Trp side chain could facilitate the passage of ions, *i.e.,* an activation of ion flux, and consequently a higher production of virus particles. This hypothesis might also be valid for model 1.

Mutation F26W *(Monocistronic virus)*

$TCID_{50}$ vs. WT : reduced 234-fold

aa natural variability:
F,I,L,M
hydrophobic position

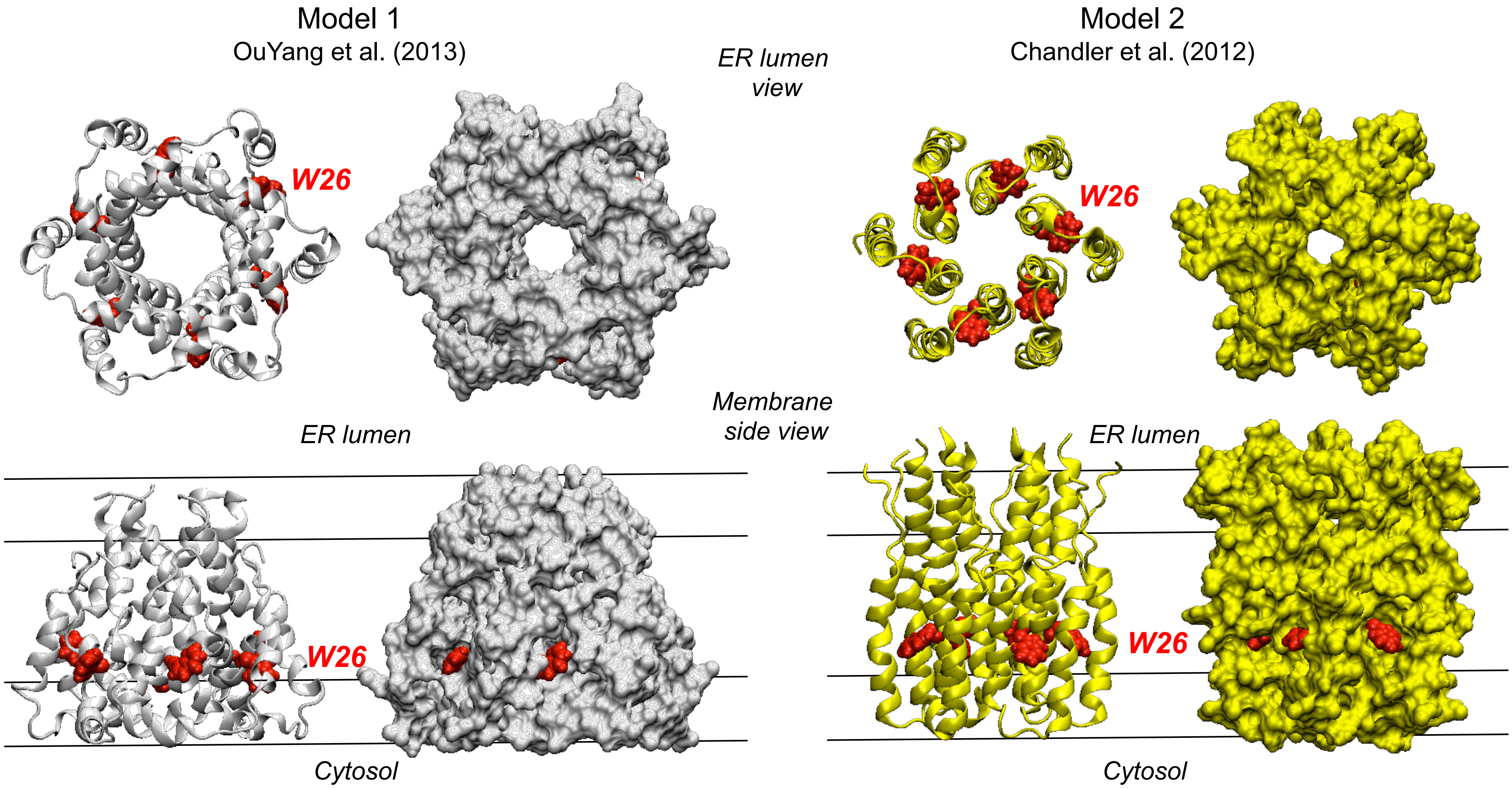

In both models, residue F26 does not access the pore lumen but is involved in numerous contacts within p7. Thus, it is likely to play a major role in protein assembly. The mutation F26W could disturb the stability of this assembly and/or the functional dynamics of this assembly, leading to a malfunctioning of p7 ion channel. In addition, the Trp side chain is more accessible to the protein surface than that of Phe, and could thus disturb some interactions with p7-interacting partners.

Mutation V27W *(Monocistronic virus)*

$TCID_{50}$ vs. WT : equivalent

aa natural variability:
C,V,I,T
variable position

Model 1
OuYang et al. (2013)

Model 2
Chandler et al. (2012)

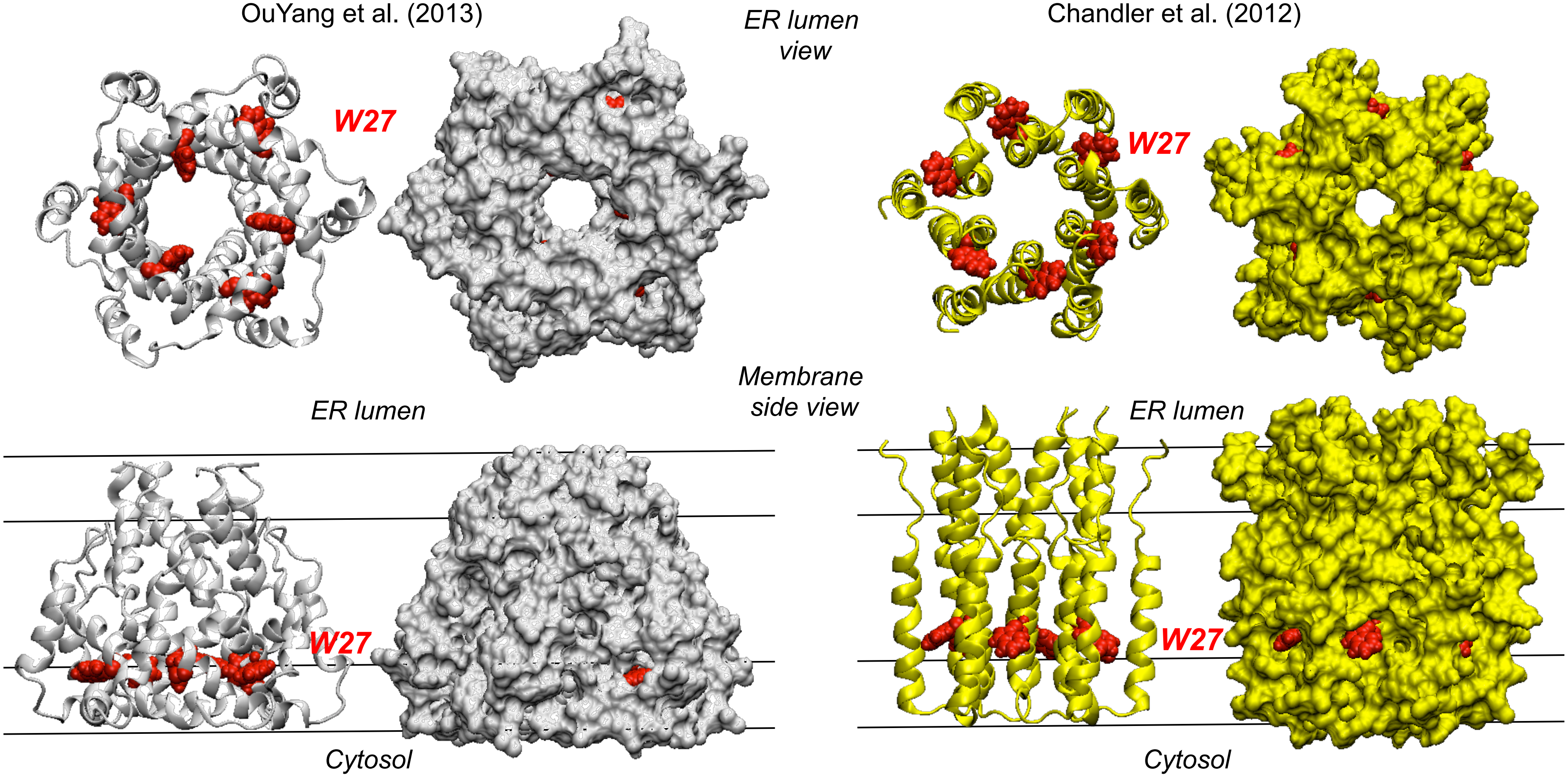

In model 1, the V/W27 side chain can access both the pore lumen and subunit interface. This ambivalence could explain the absence of effect of V27W mutation (although a modulation of ion flux would be expected).

In model 2, the V/W27 side chain is at the subunit interface and is partly accessible at the protein surface at the level of the membrane hydrophobic core. The lack of an effect of this mutation suggests this residue is not essential for interaction with p7 partner(s).

Mutation A28W *(Monocistronic virus)*

$TCID_{50}$ vs. WT : reduced 26-fold

aa natural variability:
A,F,I,C,V,W
variable, hydrophobic position

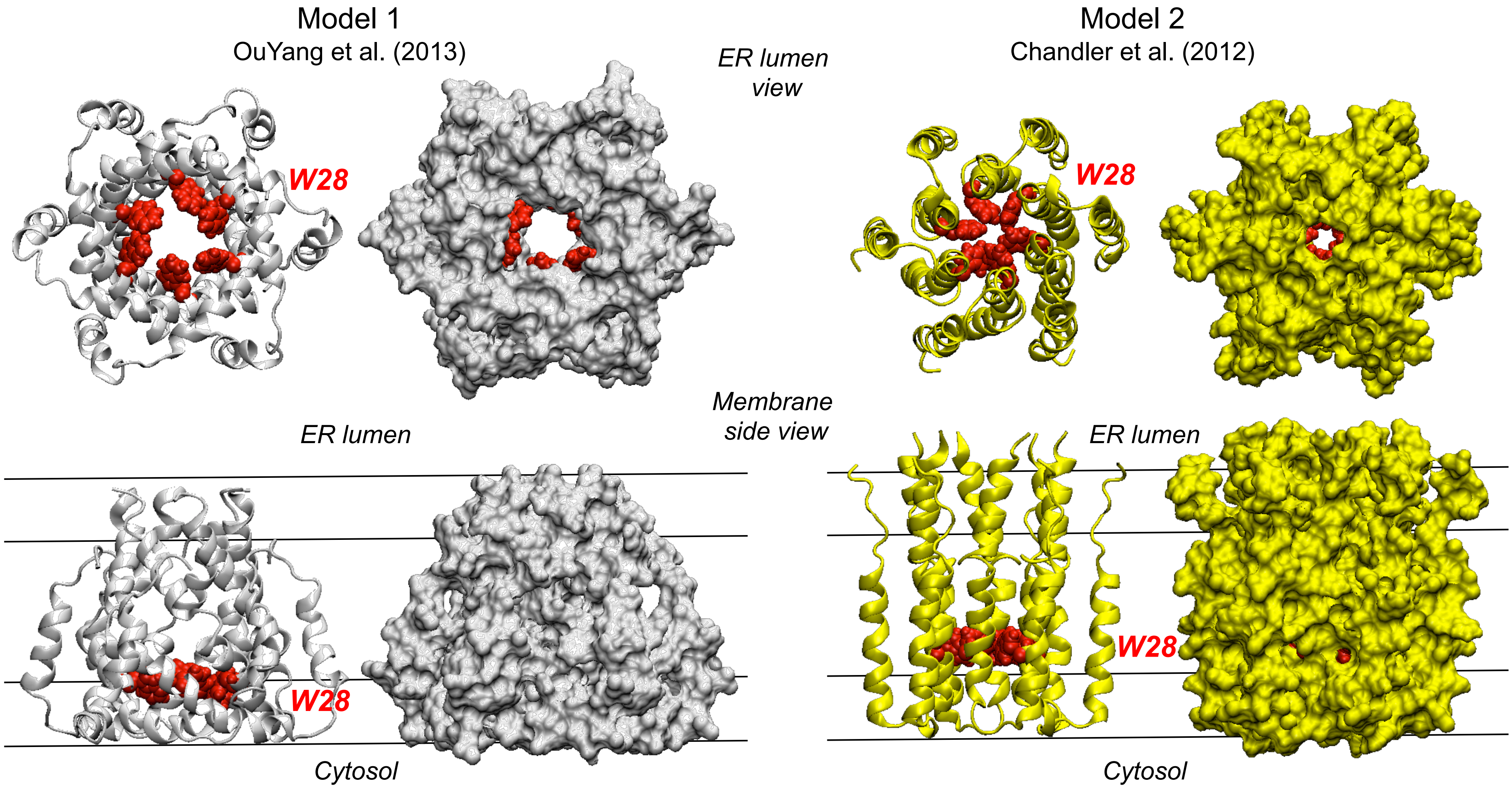

In both models, the A/W28 side chain points to the pore lumen, indicating that mutation A28W can modulate ion flux. Note, however, that a W is observed at this position in p7 of genotype 6g.

Mutation A29W *(Monocistronic virus)*

TCID$_{50}$ vs. WT : enhanced 8-fold

aa natural variability:
A,V,T,S
variable position

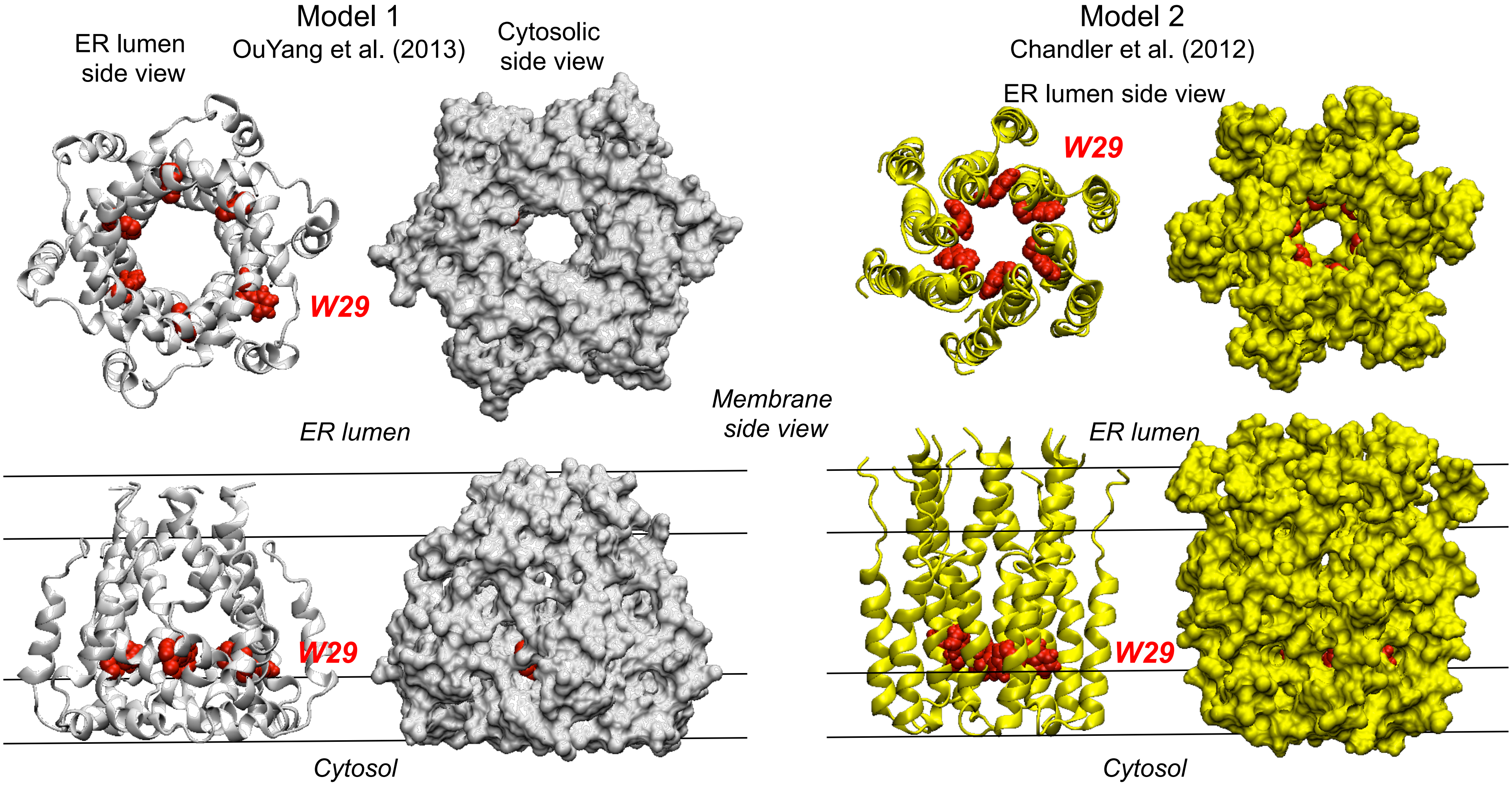

In both models, the A/W29 side chain is located at the subunit interface but also has limited access to the pore lumen, indicating that this residue could modulate ion flux. For the A29W mutant, the bulky side chain of Trp at the subunit interface might facilitate the open state of the pore, allowing an increase of p7 activity.

Mutation Y31W *(Monocistronic virus)*

$TCID_{50}$ vs. WT : enhanced 11-fold

aa natural variability:
Y,H,C
variable, polar position

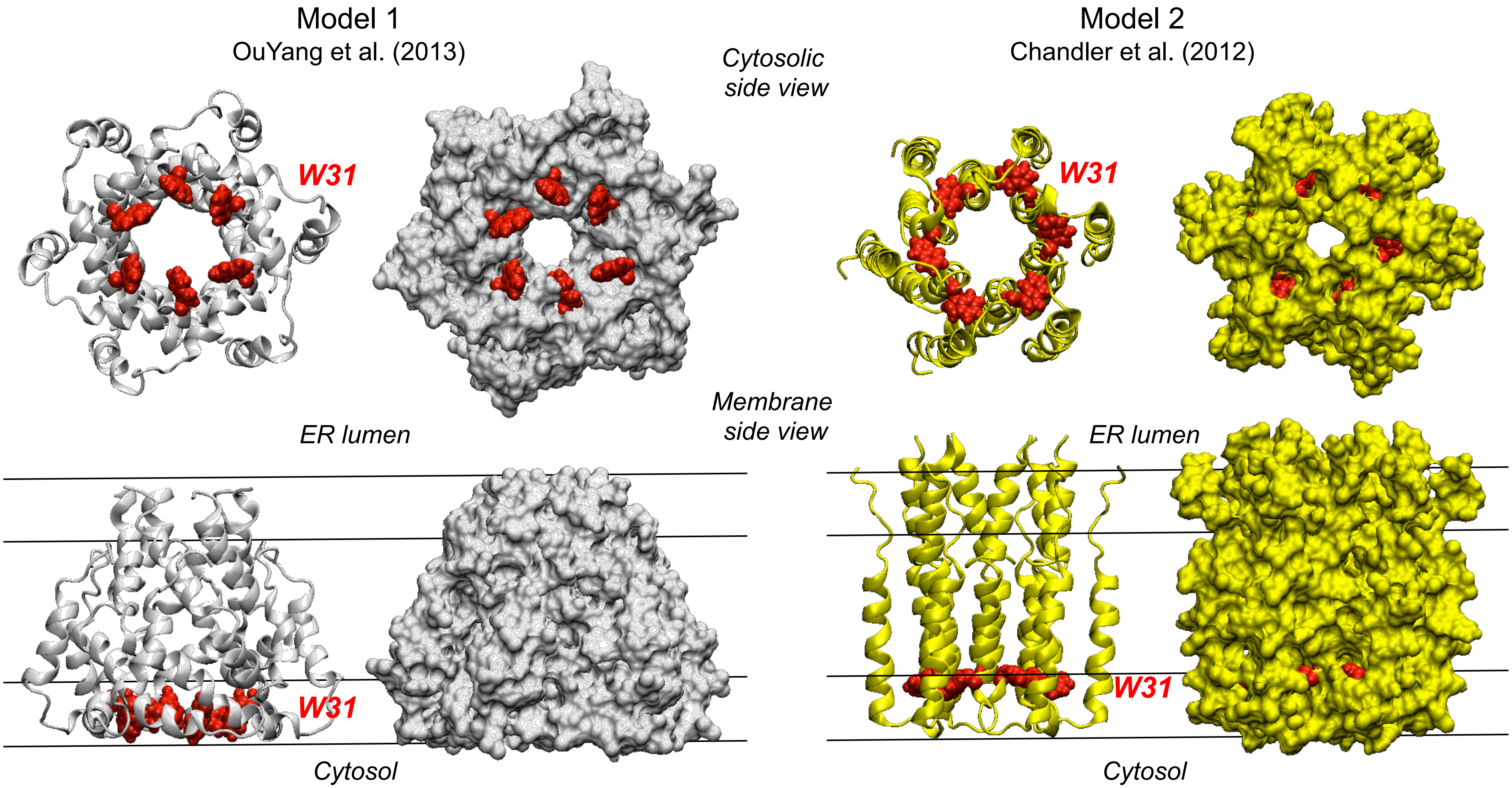

In model 1, the Y/W31 side chain is in the pore lumen. The enhanced virus production for the Y31W mutant might be explained by the similar physico-chemical properties of Trp and Tyr side chains (aromatic nature with the presence of a polar group). The amide group of Trp side chain (-NH-) maybe more favorable to facilitate ion flux than the hydroxyl group of Tyr side chain (-OH)

In model 2, the Y/W31 side chain is only partly accessible to the pore lumen. In this model, the major role of residue 31's side chain seems to be a stabilization of p7 assembly. The bulky side chain of Trp might facilitate the open state of the pore.

Mutation I32W *(Monocistronic virus)*

$TCID_{50}$ vs. WT : reduced 2-fold

aa natural variability:
I,L,V,F
hydrophobic position

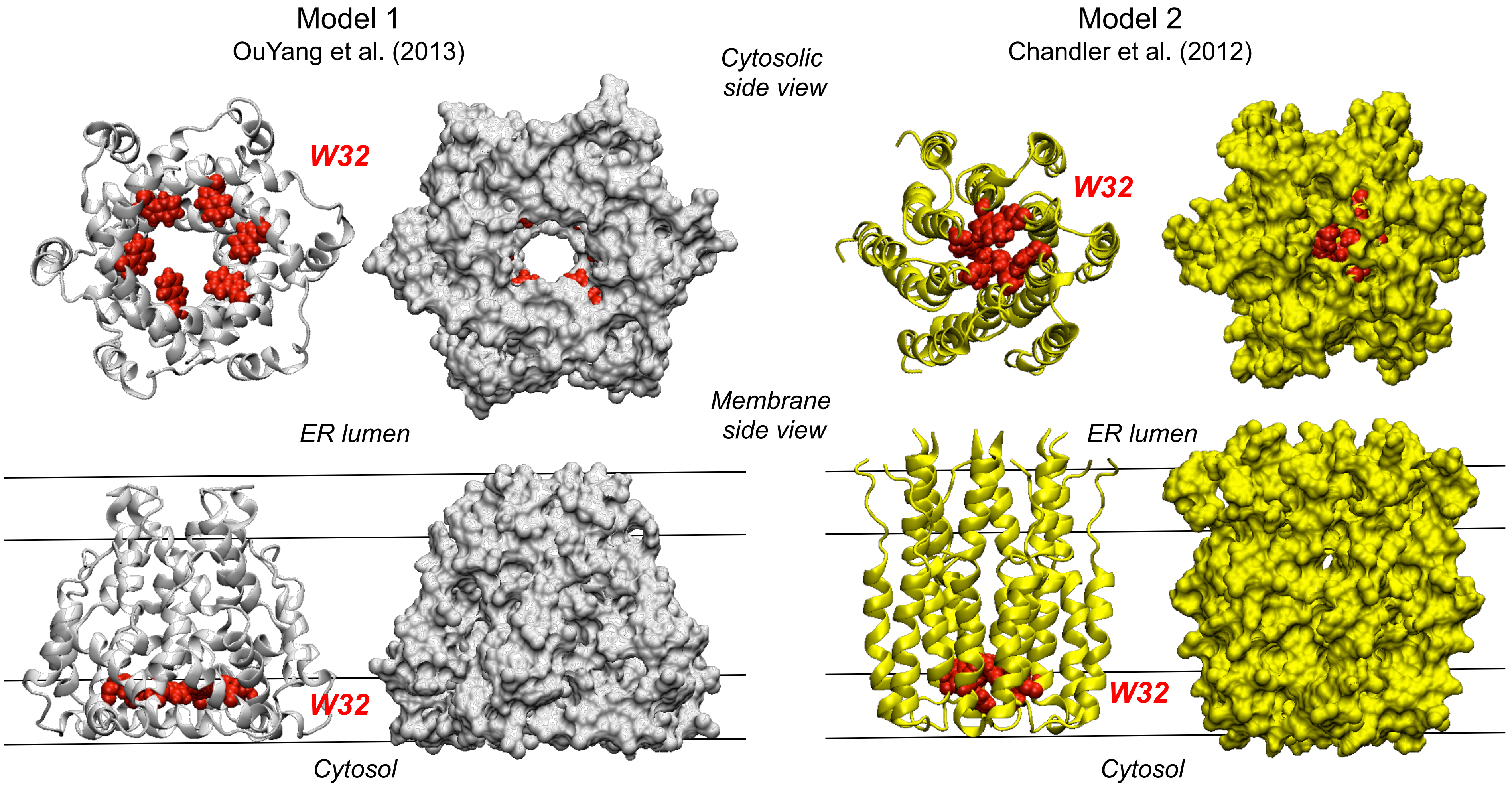

In both models, the I/W32 side chain points to the pore lumen, indicating that mutation I32W could disturb ion flux.

Mutation of basic positions 33 and 35 *(Monocistronic virus)*

Mutation to Gln (Steinmann et al. PLoS Pathogens 2007)

aa natural variability:
R,K
positively charged position

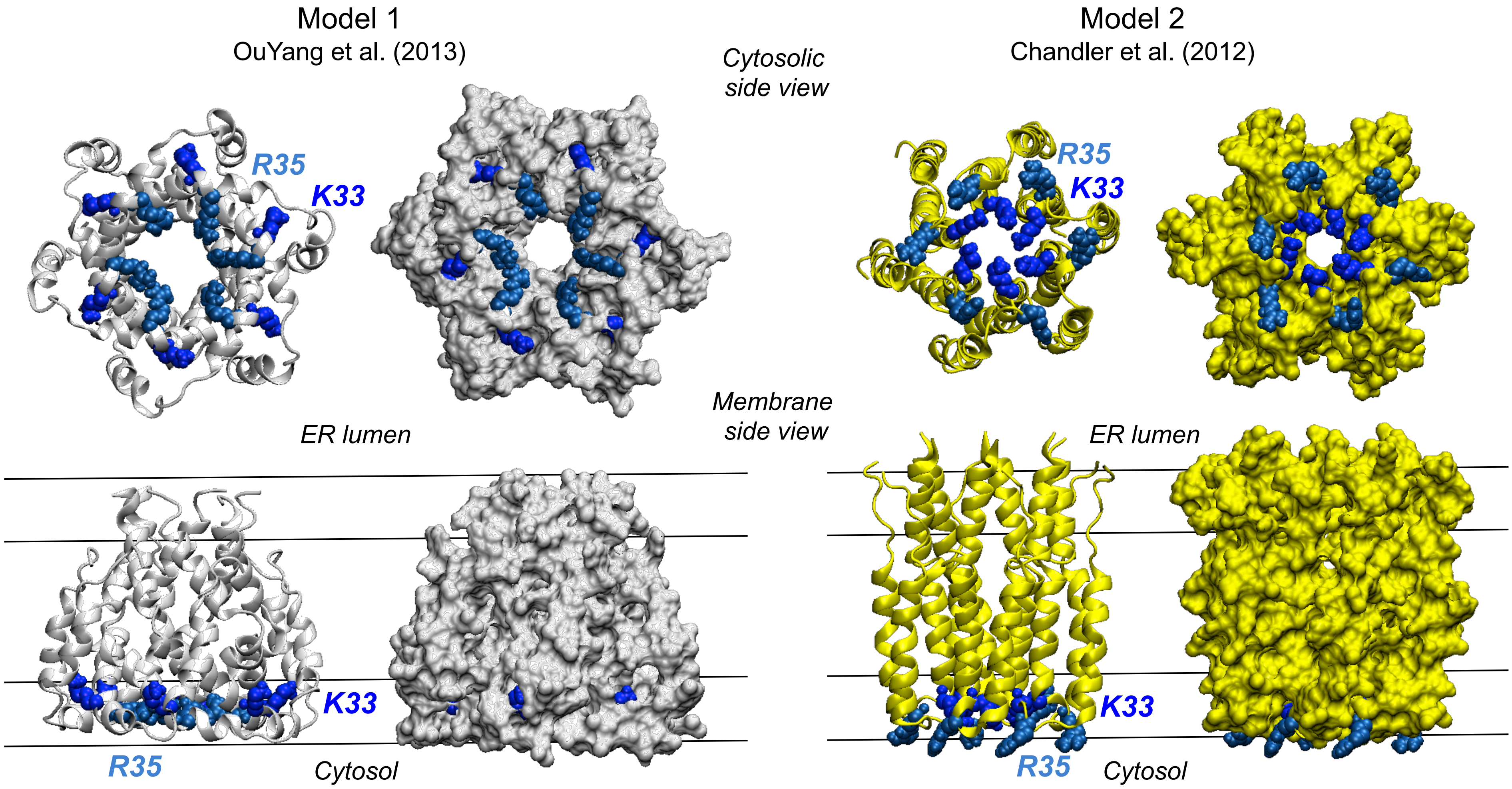

In both models, only one basic amino acid points to the pore lumen, but it is amino acid 33 in model 2 and amino acid 35 in model 1. The second basic amino acid is located at the cytosolic surface in model 2 (R35) while it is embedded in the structure and poorly accessible at the protein surface in model 1. Mutation of either position 33 or 35 to Gln reduced infectivity by about 1.2 logs while the double mutation RR33/35QQ fully abrogated infectivity (Steinmann et al. 2007).

Note that the double mutation RR33/35AA likely abrogates the correct folding of p7 and it is thus likely not relevant.

Mutation V36W *(Monocistronic virus)*

$TCID_{50}$ vs. WT : reduced 19-fold

aa natural variability:
L,W,V,I,F,A
hydrophobic position

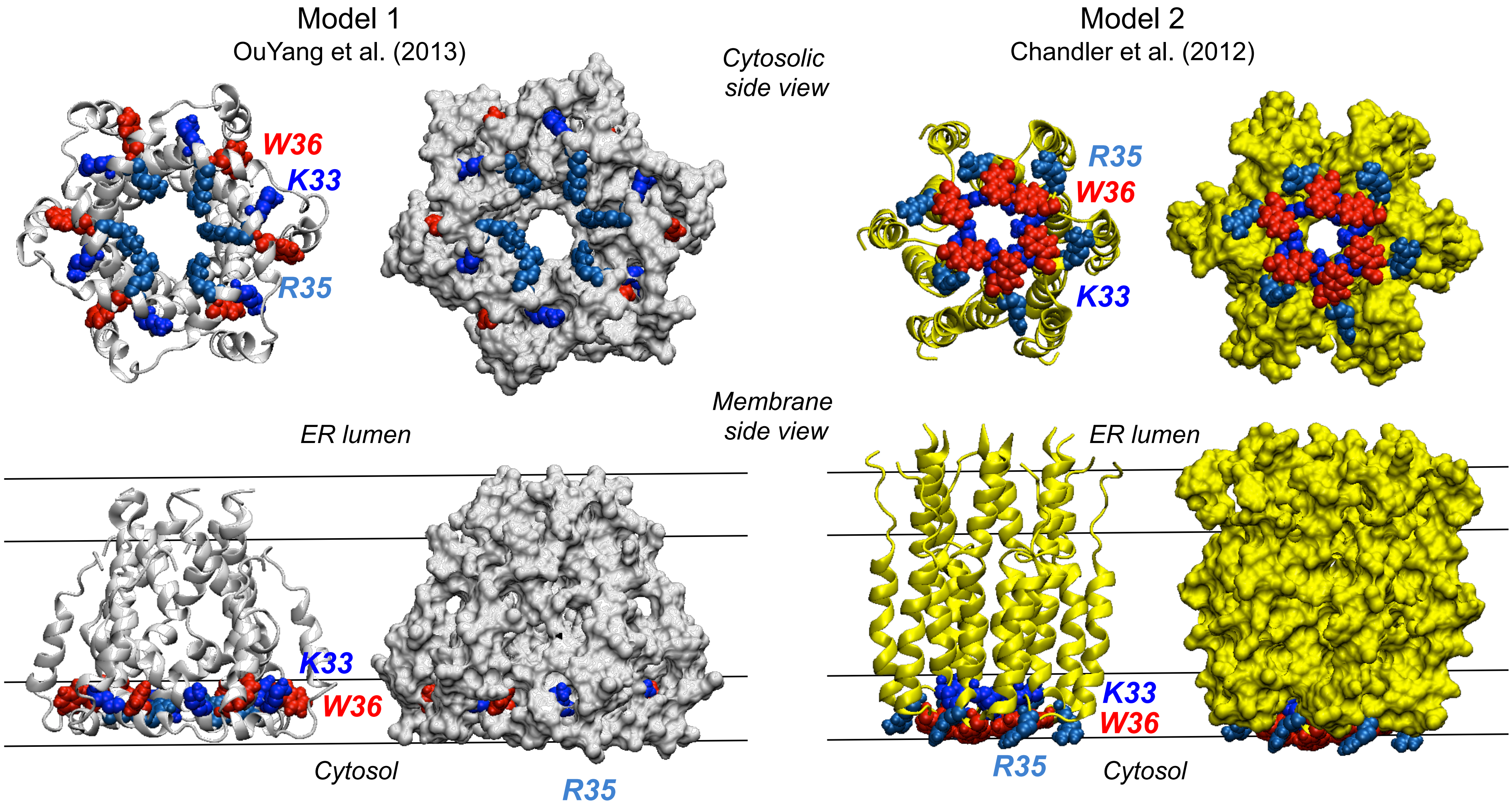

In model 1, the V/W36 side chain does not access the p7 pore lumen and is likely involved in p7 oligomerization. Mutation V36W could increase the rigidity of the overall structure, thus disturbing the functional dynamics of p7.

In model 2, the V/W36 side chain interacts with basic residues 33 and 35 on the cytosolic side, suggesting that V36W mutation could disturb the amino acid organization on the cystosolic side and directly or indirectly disturb ion channeling.

Mutation V37W *(Monocistronic virus)*

$TCID_{50}$ vs. WT : enhanced 2-fold

aa natural variability:
V,A,P,T,I,F
hydrophobic position

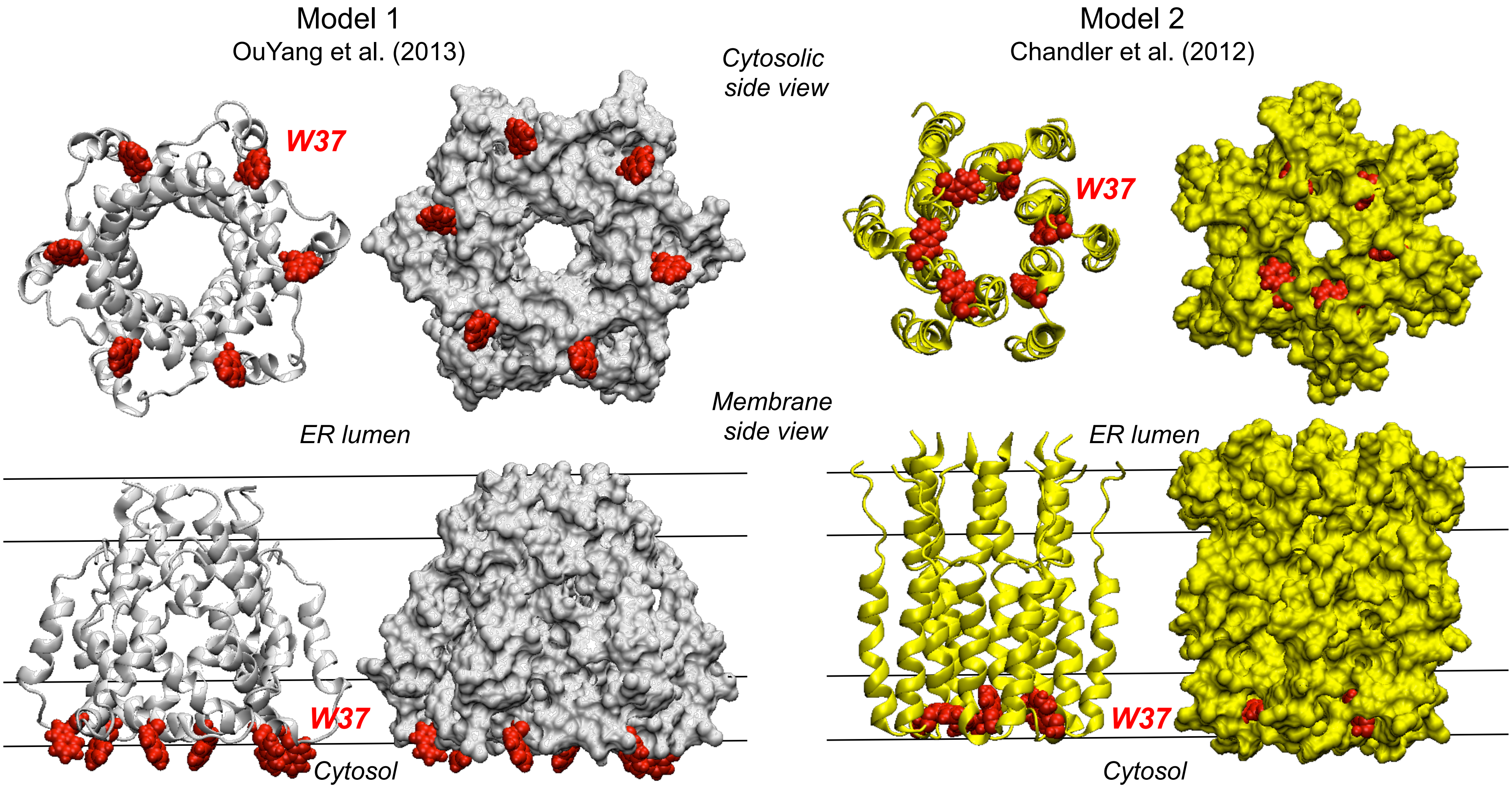

In model 1, the V/W37 side chain is fully accessible at the protein surface and located at the membrane interface where it might modulate interaction(s) with p7 partner(s) and/or lipids.

In model 2, amino acid 37's side chain is likely involved in the stability of subunit assembly. The mutation to W might rigidify this assembly. W37 is almost not accessible at the protein surface on the cytosolic side.

Mutation P38W *(Monocistronic virus)*

TCID$_{50}$ vs. WT : reduced 2-fold

aa natural variability:
P,A
hydrophobic position

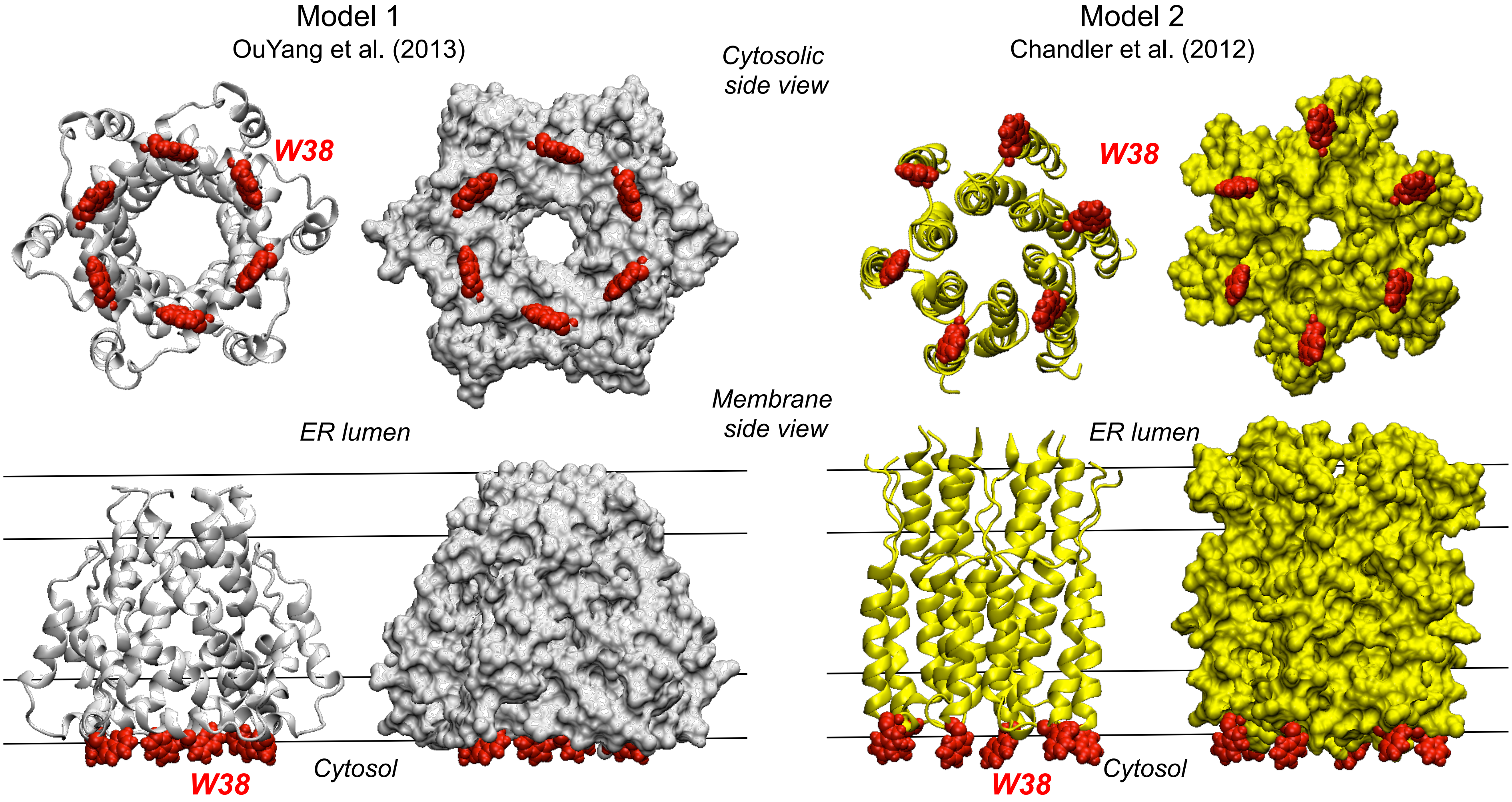

In both models, the P/W38 side chain is accessible at the protein surface on the cytosolic side. As proline is highly conserved at this position, it should have an important structural role, potentially in the conformational changes required for p7 functioning. Trytophan should not ensure such a role, explaining the deleterious effect of P38W mutation.

Mutation L39W *(Monocistronic virus)*

$TCID_{50}$ vs. WT : reduced 2-fold

aa natural variability:
G,L,A,V,I,M
variable position

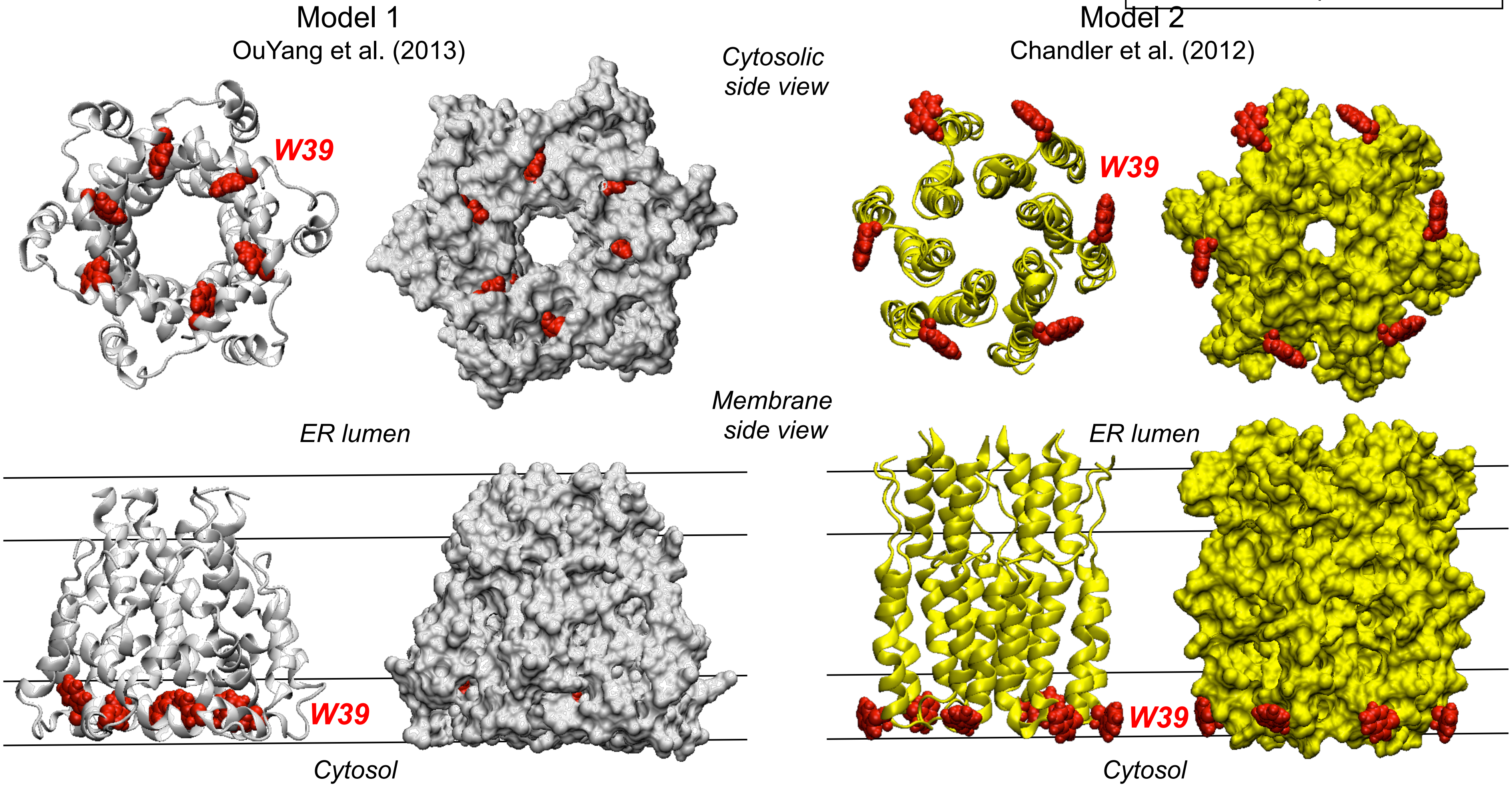

In model 1, amino acid 39's side chain is likely involved in the stability of subunit assembly. The mutation in Trp might rigidify this assembly, limiting conformational changes required for p7 functioning.

In model 2, the L/W39 side chain is accessible at the protein surface and located at the membrane interface where it might modulate interaction(s) with p7 partner(s) and/or lipids. This could explain the slight deleterious effect of this mutation.

Mutation A40W *(Monocistronic virus)*

$TCID_{50}$ vs. WT : enhanced 2-fold

aa natural variability:
A,V,T,L,C,F,M
variable position

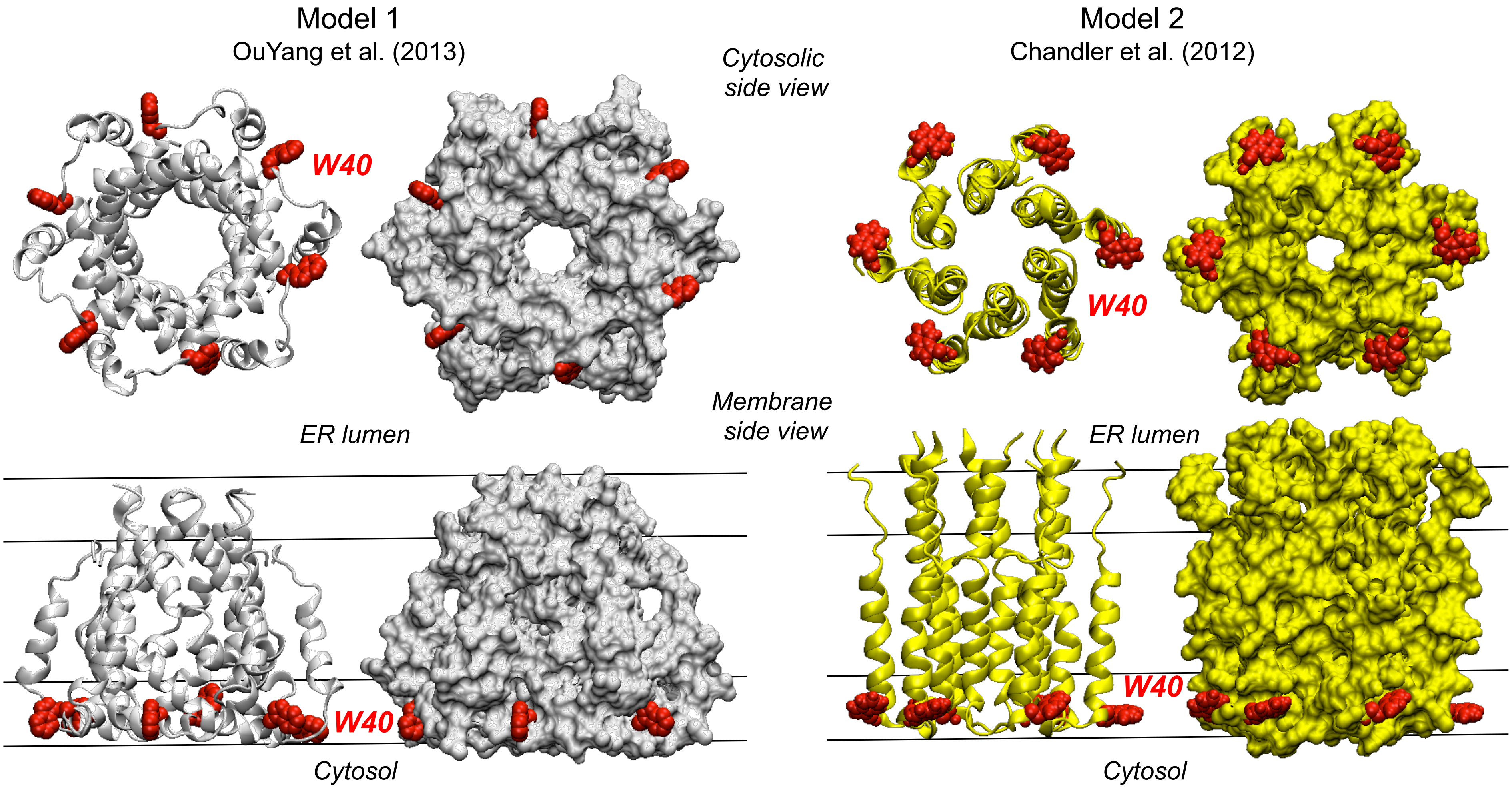

In both models, the A/W40 side chain is accessible at the protein surface and located at the membrane interface. W41 might modulate interaction(s) with p7 partner(s) and/or lipids. This could explain the positive effect of this mutation.

Mutation T41W *(Monocistronic virus)*

$TCID_{50}$ vs. WT : enhanced 4-fold

aa natural variability:
A,T,V
variable position

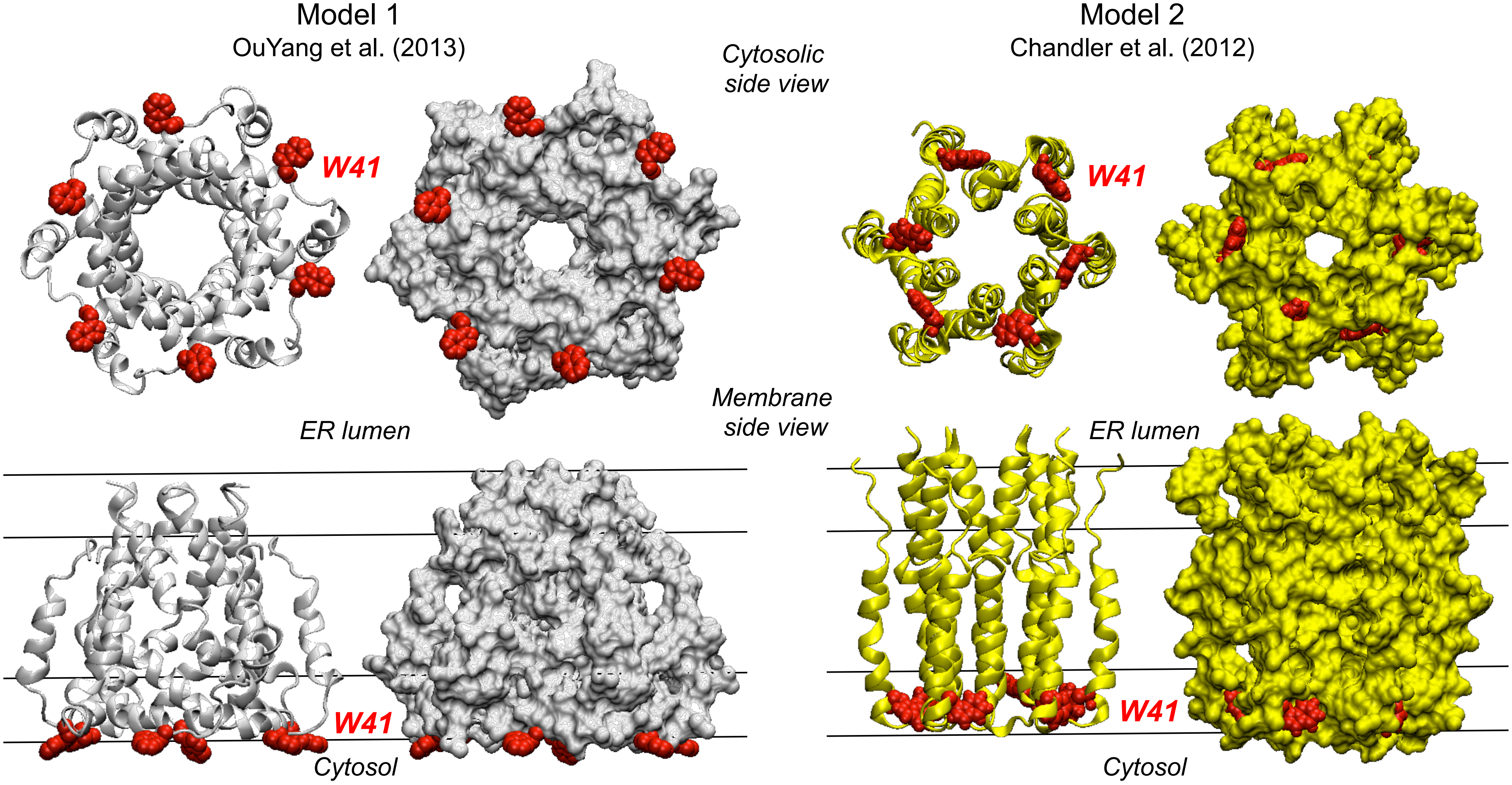

In both models, the T/W41 side chain is accessible at the protein surface and located at the membrane interface. W41 might modulate interaction(s) with p7 partner(s) and/or lipids. This could explain the positive effect of this mutation. In model 2, W41 might stabilize subunits interactions, which could have a positive effect on p7 functioning.

Mutation Y42W *(Monocistronic virus)*

$TCID_{50}$ vs. WT : reduced 9-fold

aa natural variability:
Y only
aromatic, polar position

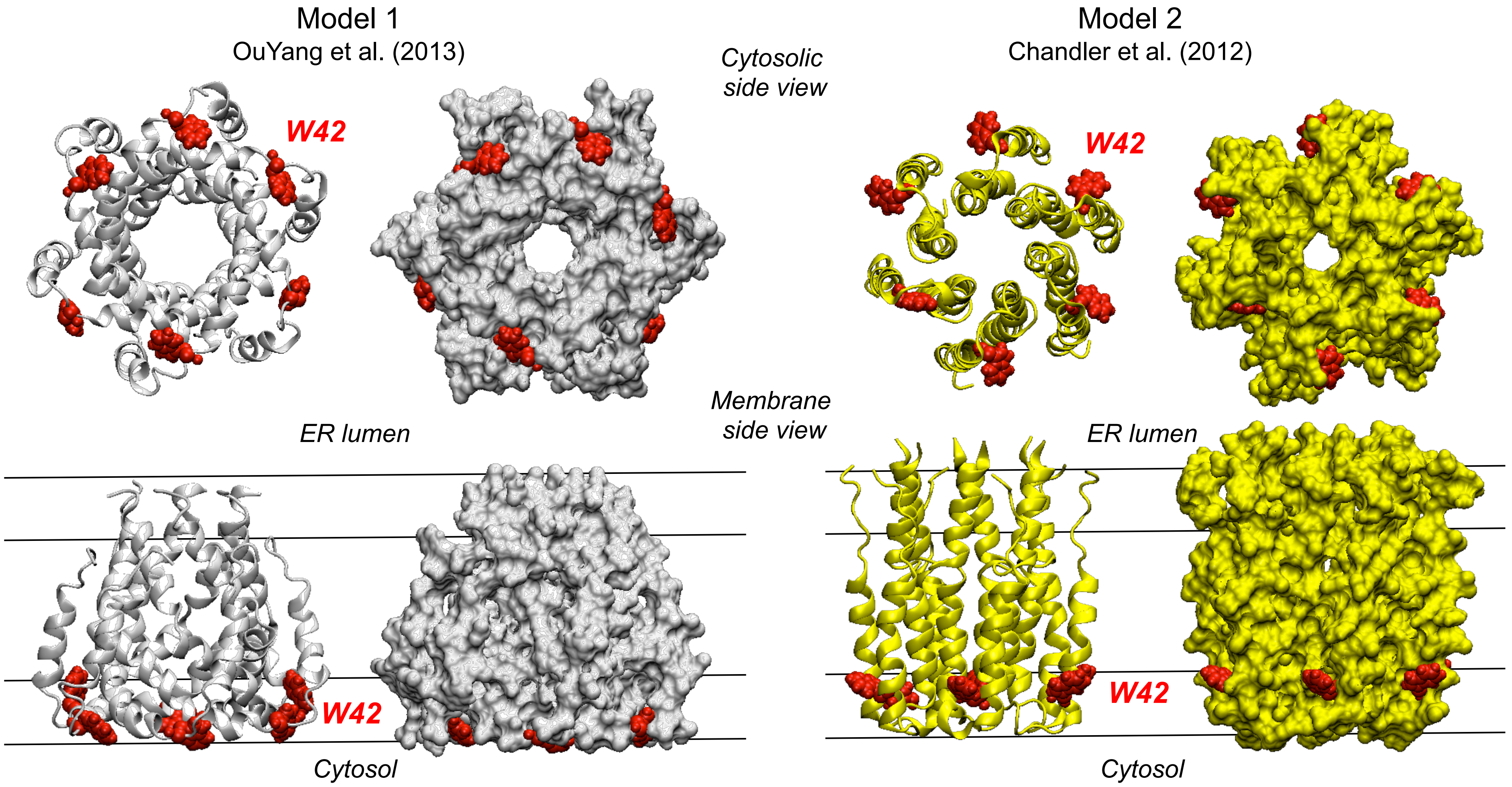

Tyr at position 42 is completely conserved across all genotypes, indicating an essential role in the structure/function/interaction of p7. In both models, the Y/W42 side chain is accessible at the protein surface and located at the level of membrane interface. Y42W mutations could disturb interaction(s) with p7 partner(s) and/or lipids. This could explain the deleterious effect of this mutation. In model 2, Y42 is involved in intramolecular interaction through aromatic ring stacking; W42 could disturb this interaction.

Mutation S43W *(Monocistronic virus)*

$TCID_{50}$ vs. WT : reduced 6-fold

aa natural variability:
A,S,G,C,T,L,M
variable position

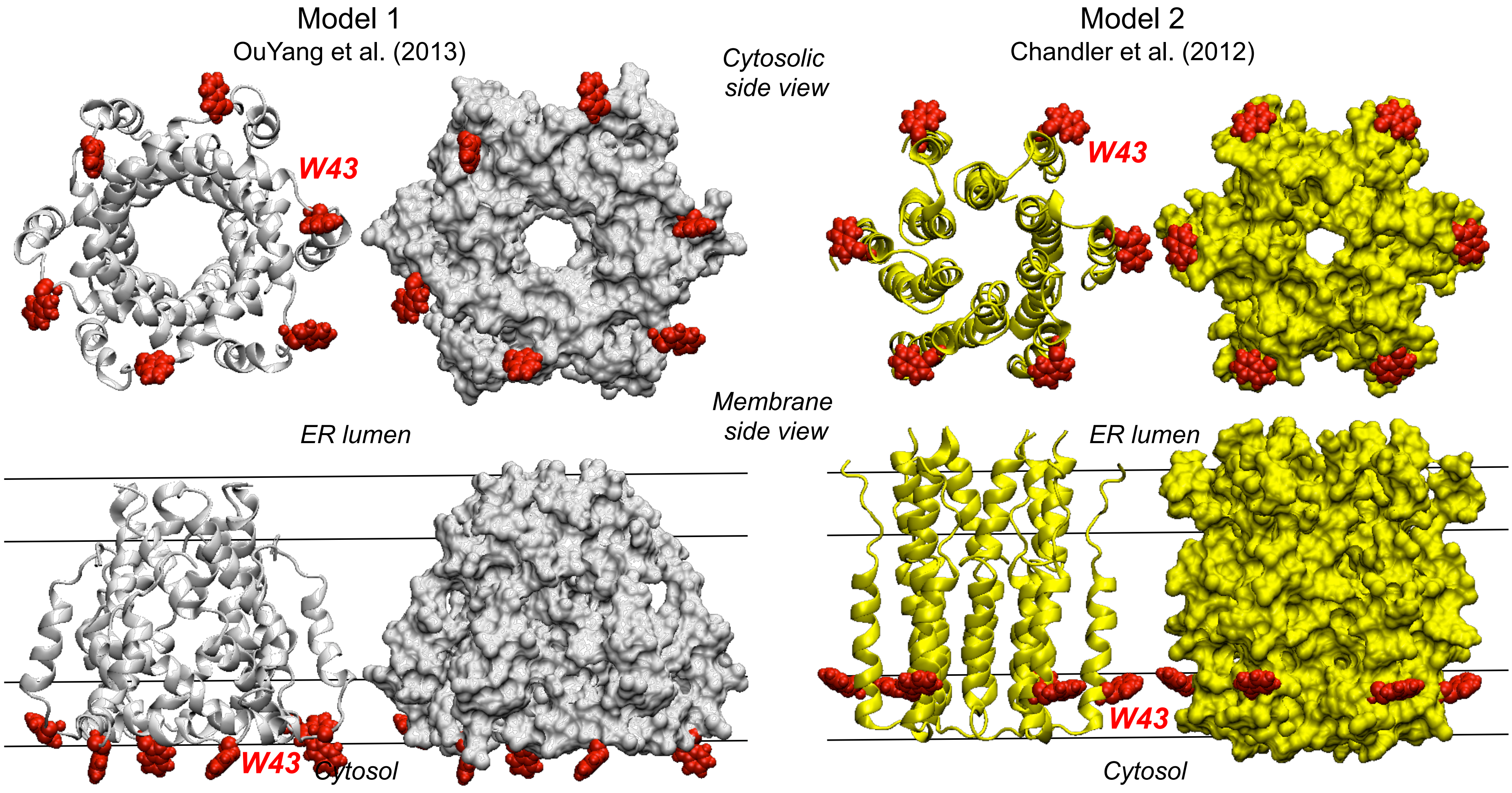

In both models, the S/W43 side chain is accessible at the protein surface and is located at the membrane interface. W43 might disturb interaction(s) with p7 partner(s) and/or lipids. This could explain the deleterious effect of this mutation.
