# Supplementary figures and images for: The N-terminal Helical Region of the Hepatitis C Virus p7 Ion Channel Protein Is Critical for Infectious Virus Production
Source: PLoS Pathog. 2015 Nov 20;11(11):e1005297. doi: 10.1371/journal.ppat.1005297 (PMC4654572; doi:10.1371/journal.ppat.1005297)

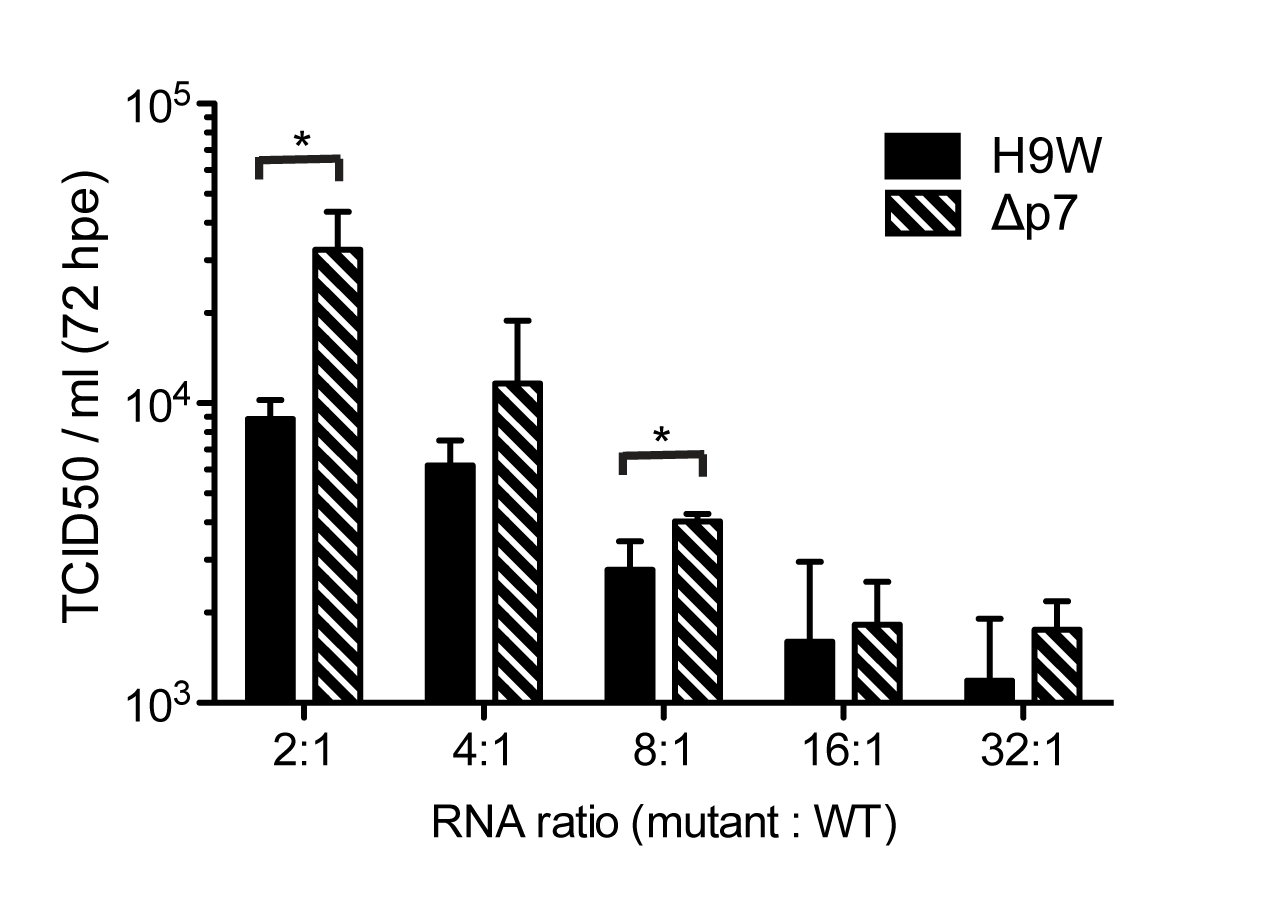

Supplement: S2 Fig — Infectious virus in the supernatants of Huh-7.5 cells electroporated with a mix of wild-type RNA and either H9W (2.5 μg) or Δp7 RNA (2.5 μg). The total amount of RNA electroporated across conditions was kept constant (5 μg) by adding additional Δp7 RNA as needed. Infectious virus titers 72 hpe were quantified by limiting dilution assay on naïve Huh-7.5 cells. Data represent the mean and standard deviation of (n = 3) independent electroporations. Statistically significant differences in titers were determined by unpaired t-tests. * p<0.05. (TIF) [file ppat.1005297.s002.tif]

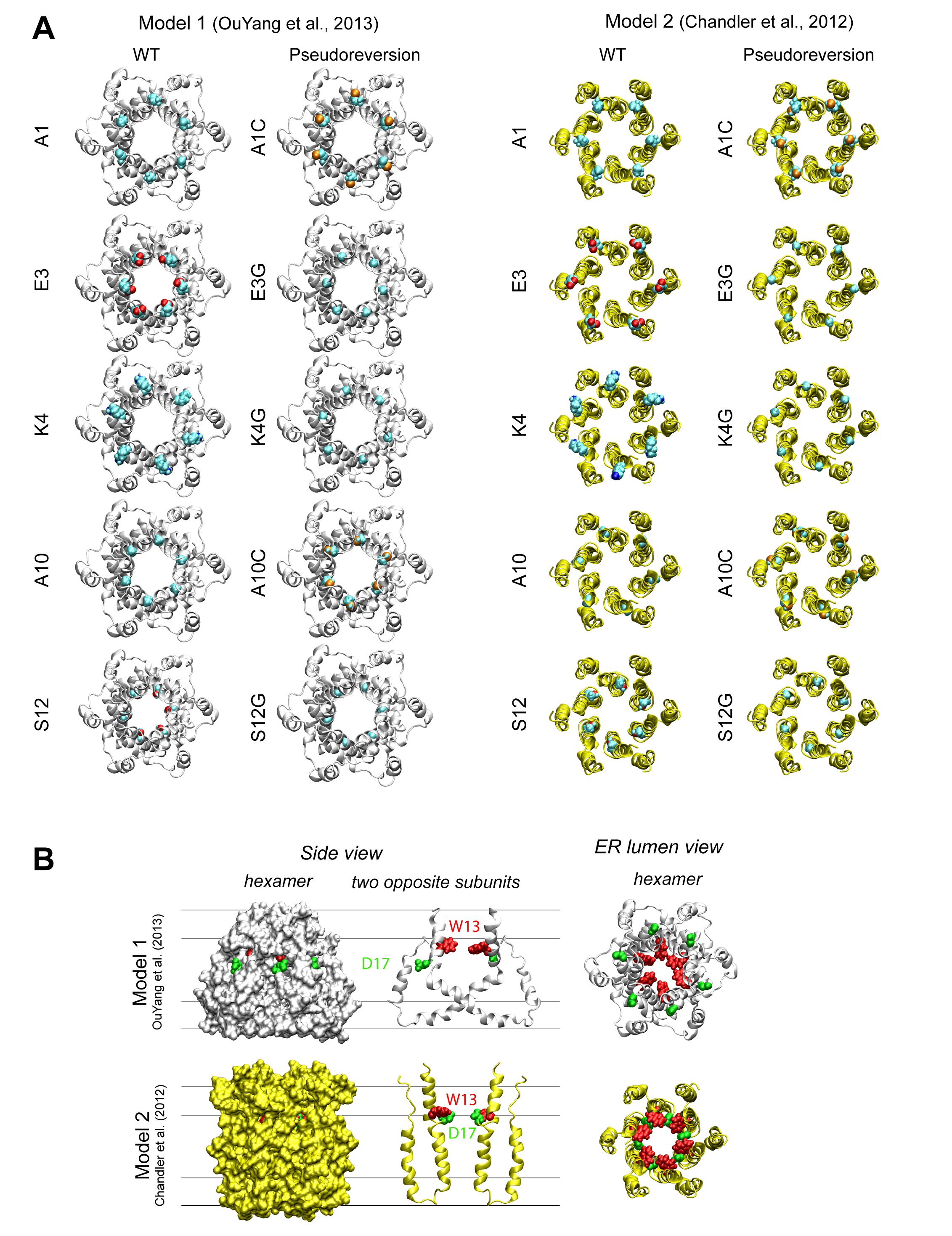

Supplement: S3 Fig — A) Top view (ER lumen side view) of p7 models 1 and 2 are shown in ribbon representations for both the natural (wild-type) amino acid and amino acid identified at the same position after passage of the Trp substitution mutant in Huh-7.5 cells. The side-chain atoms of natural and pseudorevertant residues are represented as spheres of the corresponding van der Waals radius. Carbon and hydrogen atoms are in cyan, oxygen atoms in red, nitrogen atoms in blue, and sulfur atoms in yellow. B) Location of A13W and N17D mutations in the hexameric forms of p7 models 1 and 2 (surface and ribbon representations from different viewpoints) and on two opposing subunits within the hexamers. Lines shown in the left hand panels represent the membrane interfaces and hydrophobic core (between the middle two lines). For more details see the legend for Fig 2. (TIF) [file ppat.1005297.s003.tif]

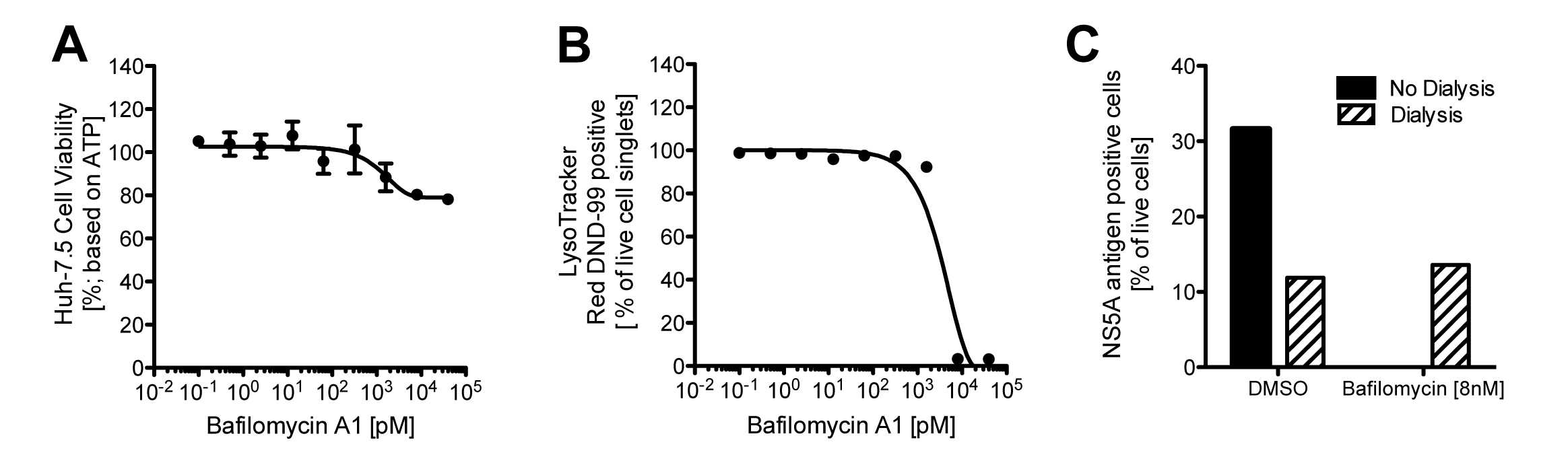

Supplement: S4 Fig — A) The effect of bafilomycin A1 on cell viability (CellTiter-Glo assay; Promega) and B) acidic intracellular pH indicated by LysoTracker Red DND-99 staining and quantified by flow cytometry. Based on these data, a concentration of 8 nM bafilomycin was used in subsequent assays. C) The impact of bafilomycin A1 on HCV entry into Huh-7.5 cells before and after dialysis indicating dialysis is required to determine infectivity in 8 nM bafilomycin-treated samples, but that some loss of overall titer occurs during buffer exchange. (TIF) [file ppat.1005297.s004.tif]

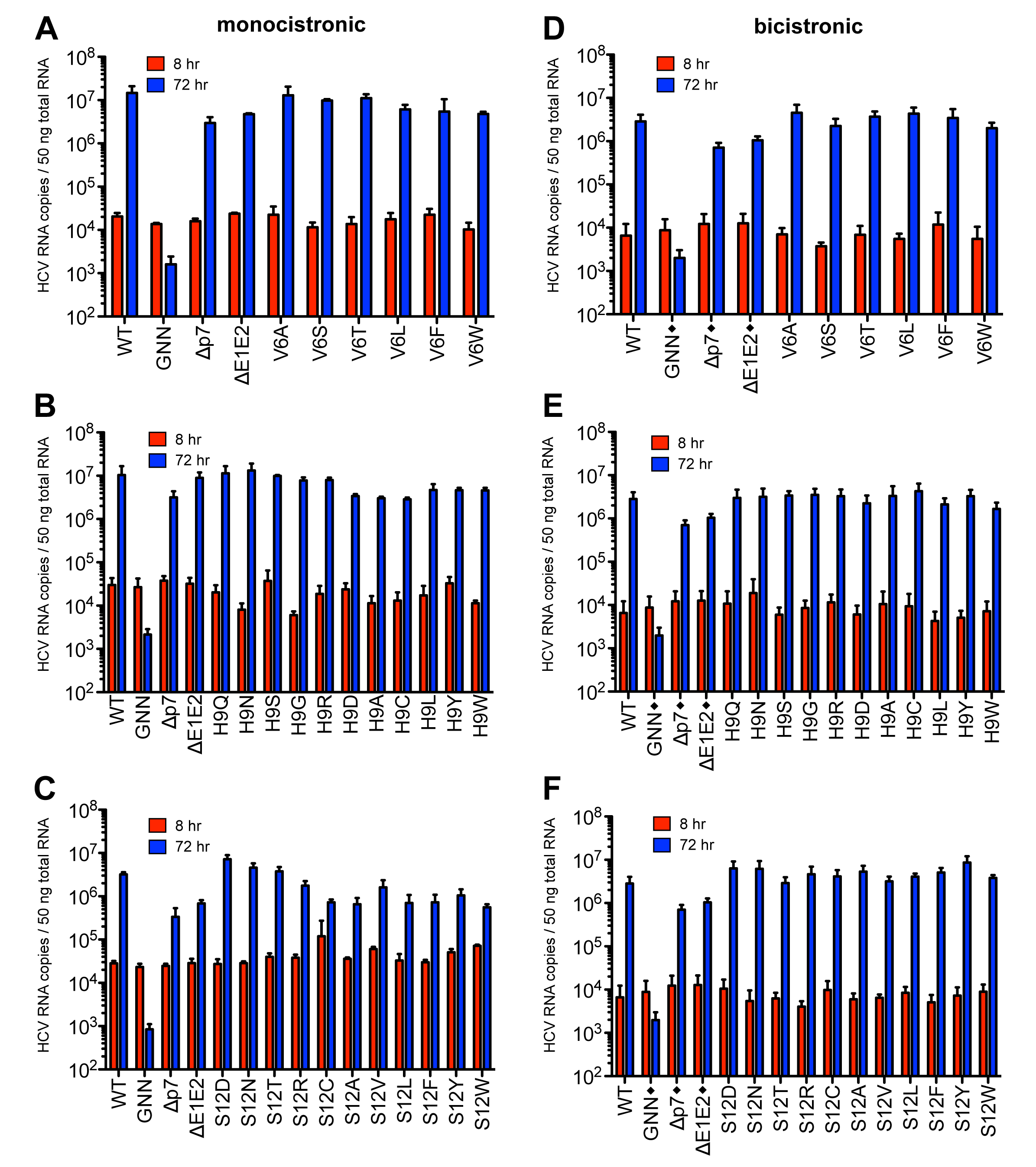

Supplement: S5 Fig — HCV RNA levels in Huh-7.5 cells determined 8 and 72 hpe showing all monocistronic (A, B & C) and bicistronic (D, E & F) p7 mutants replicate efficiently. Black diamond (◆) indicates that these control viruses are monocistronic. Note that the WT, GNN, Δp7, and ΔE1E2 titers reported in panels E and F are duplicated from panel D as these viruses were all analyzed in parallel. Data shown correspond to infectious virus titers reported in Fig 7. (TIF) [file ppat.1005297.s005.tif]

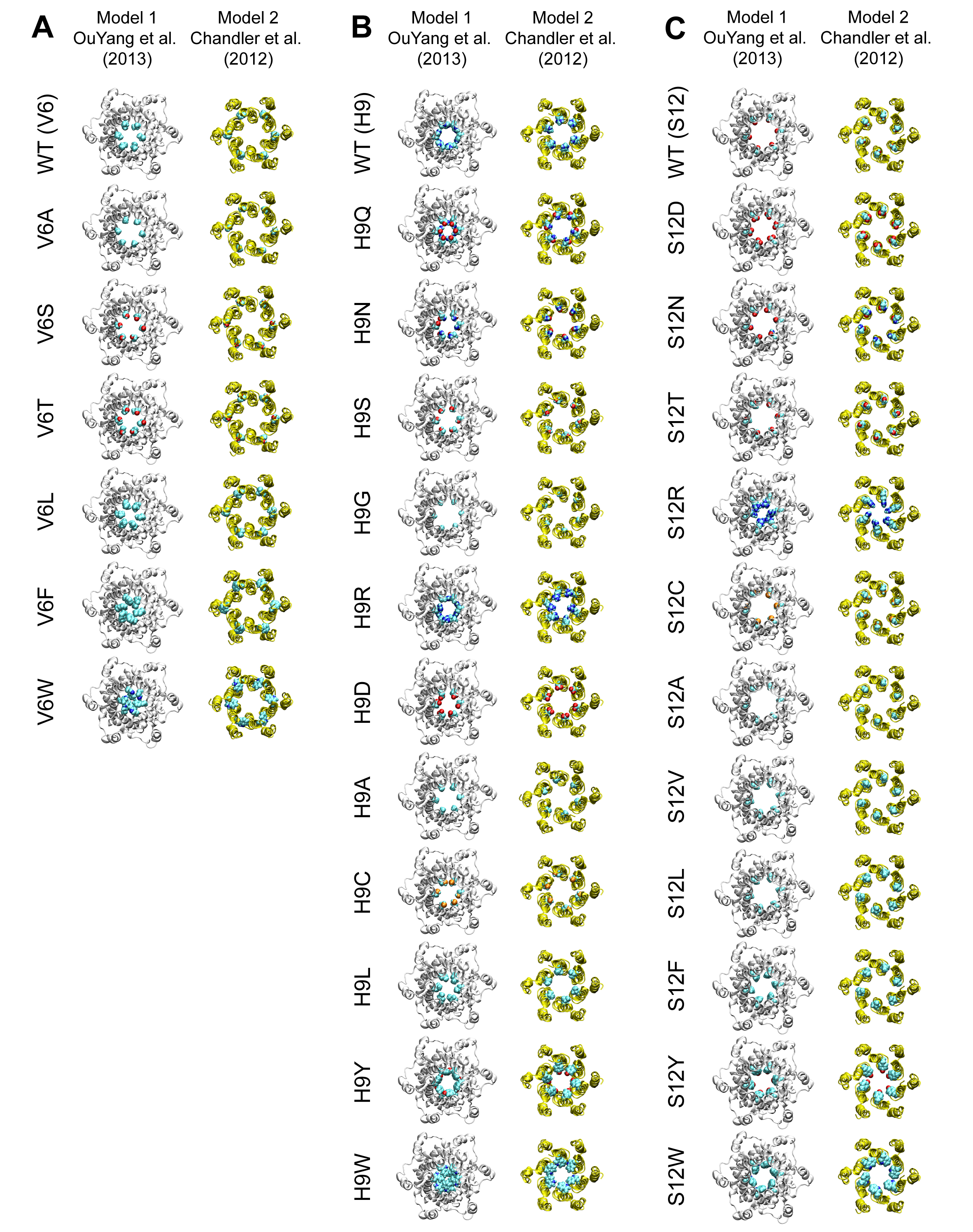

Supplement: S6 Fig — Top view (ER lumen side view) of p7 models 1 and 2 are shown in ribbon representations for all amino acids tested at positions A) 6, B) 9 and C) 12. The side-chain atoms of residues are represented as spheres of the corresponding van der Waals radius. Carbon and hydrogen atoms are in cyan, oxygen atoms in red, nitrogen atoms in blue, and sulfur atoms in yellow. (TIF) [file ppat.1005297.s006.tif]
